# Design, Synthesis, and Biological Evaluation of Novel *N*‐Acyl Sulfonohydrazides Derived From β‐Hydroxy Esters: Anticancer, Antioxidant, and Antimicrobial Activities

**DOI:** 10.1111/cbdd.70344

**Published:** 2026-06-19

**Authors:** Belma Hasdemir, Tülay Yıldız, Hasniye Yaşa, Hatice Başpınar Küçük, Emel Mataracı Kara, Sümbül Yıldırım, Fatih Kocabaş, Ziya Can

**Affiliations:** ^1^ Department of Chemistry, Organic Chemistry Division, Faculty of Engineering Istanbul University‐Cerrahpaşa Istanbul Turkey; ^2^ Department of Pharmaceutical Microbiology, Faculty of Pharmacy Istanbul University Istanbul Turkey; ^3^ Department of Genetics and Bioengineering, Faculty of Engineering Yeditepe University Istanbul Turkey; ^4^ Department of Molecular Biology and Genetics, Faculty of Engineering and Natural Sciences Istanbul Atlas University Istanbul Turkey; ^5^ Department of Chemistry, Analytical Chemistry Division, Faculty of Engineering Istanbul University‐Cerrahpaşa Istanbul Turkey

**Keywords:** anticancer, antimicrobial, antioxidant, apoptosis assay, sulfonohydrazide

## Abstract

In this study, a series of novel *N*‐acyl sulfonohydrazides **6a–r** derived from β‐hydroxy esters was synthesized, and their structures were confirmed through spectroscopic characterization, including FT‐IR, ^1^H NMR, ^13^C NMR, and HRMS analyses. The anticancer properties of the synthesized compounds were investigated, along with a complementary study evaluating their antioxidant and antimicrobial properties. The cytotoxicity screening against HCT116 and PANC1 cancer cell lines revealed generally low antiproliferative effects; however, compounds **6c** and **6e** demonstrated relatively higher pro‐apoptotic potential in HCT116 cells compared to the other compounds. In assays evaluating antioxidant capacity, utilizing both CUPRAC and DPPH methods, all synthesized compounds exhibited activity ranging from moderate to good. Notably, compound **6l** demonstrated the strongest antioxidant performance with TEAC = 1.34 and IC_50_ = 0.084 ± 0.003 mmol L^−1^. Furthermore, among the tested compounds, **6a**, **6b**, and **6d** displayed good antibacterial activity against *Staphylococcus aureus* with a minimum inhibitory concentration (MIC) of 19.53 μg/mL.

## Graphical abstract

Eighteen novel *N*‐acyl sulfonohydrazide derivatives **6a–s** were designed and synthesized. All compounds were evaluated in vitro for their antibacterial, antifungal, anticancer, and antioxidant activities. Molecular docking studies were also performed. Compounds **6c** and **6e** emerged as the most promising anticancer agents, while **6l** showed the highest antioxidant capacity.

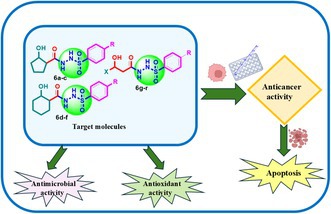

## 1 Introduction

**FIGURE 1:**
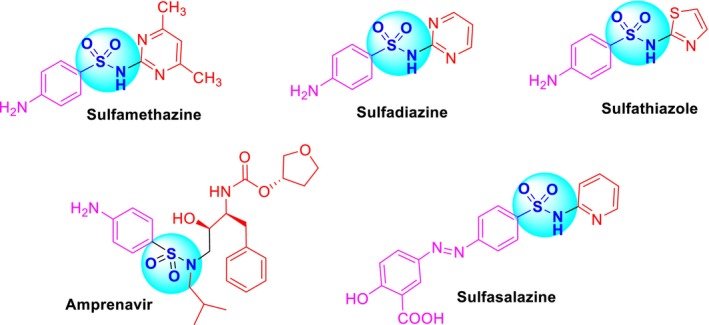
Some clinical drugs bearing the sulfonamide moiety.

Sulfonohydrazides, bearing the –SO_2_NHNH_2_ and/or –SO_2_NHNNHR– functional motifs, represent a versatile class of organosulfur compounds with significant potential in drug discovery (Figure 1) (Li et al. 2020; Macara et al. 2023; Omar et al. 2025). In particular, *N*‐acyl sulfonohydrazides have attracted increasing attention due to their diverse biological activities, including anti‐inflammatory (Hussein et al. 2025; Rahman et al. 2025), antimicrobial (Doğan et al. 2023; Hadi et al. 2021; Palathoti et al. 2026), antiviral (Khan et al. 2025), antidiabetic (Abdelazeem et al. 2024; Ayoup et al. 2024), diuretic (Temperini et al. 2008), antioxidant (Bozkurt et al. 2020), carbonic anhydrase, and α‐glucosidase inhibitors (Bozkurt et al. 2020; Sun et al. 2025; Ullah et al. 2023) effects. *N*‐acyl sulfonohydrazide derivatives have emerged as promising scaffolds due to their structural flexibility and ability to engage in multiple hydrogen‐bonding and electrostatic interactions with biological targets. Together with their reported biological activities, these properties make them attractive scaffolds for anticancer drug development. Cancer remains a major global health issue, and therefore, there is a constant need to develop new therapeutic agents that target key molecular pathways. Previous studies have demonstrated that sulfonohydrazide derivatives can exert cytotoxic effects across various cancer cell lines, supporting their potential as lead compounds in anticancer drug development (El‐Gaby et al. 2020; Elsayad et al. 2024; Gaur et al. 2022; Kannigadu et al. 2022; Wan et al. 2021; Sabbah et al. 2021). In addition to cytotoxicity, oxidative stress is closely associated with cancer progression, as well as other pathological conditions. Reactive oxygen species (ROS) contribute to DNA damage, genomic instability, and tumor progression, while modulation of oxidative balance can influence cancer cell survival and therapeutic response (Nakamura and Takada 2021). Evaluating antioxidant activity alongside anticancer potential can provide supportive insights into the possible mechanisms of action of newly synthesized compounds.

Accordingly, the primary focus of the present study is the design and synthesis of novel *N*‐acyl sulfonohydrazide derivatives that may exhibit potential anticancer properties. For this purpose, a series of *N*‐acyl sulfonohydrazide compounds was synthesized from β‐hydroxy ester precursors, and their structures were characterized using various spectroscopic methods. The anticancer activities of these compounds were evaluated in vitro. Additionally, binding interactions with relevant targets were investigated (see the Supporting Information (SI) for molecular docking studies). Moreover, their antioxidant and antimicrobial activities were examined to screen for other potential pharmacological properties.

## 2 Design of the Target Molecules

In this study, β‐hydroxy esters, recognized as biologically active scaffolds, were selected as precursor compounds. A literature survey reveals that β‐hydroxy esters and derivatives are important synthetic intermediates for numerous biologically active compounds exhibiting antioxidant, antimicrobial, anti‐inflammatory, and anticancer properties (Benlebna et al. 2021; Ritchie et al. 2011; Trujillo et al. 2006; Wu et al. 2014). For example, β‐hydroxy‐β‐aryl propionate and its derivatives have been reported as valuable compounds for the synthesis of various drugs, such as fluoxetine and β‐lactam antibiotics (Kumar et al. 1991; Swarén et al. 1999). In our previous studies, several hydroxy ester derivatives were synthesized and evaluated for their biological activities (Başpınar Küçük et al. 2017; Hasdemir 2015; Hasdemir et al. 2012, 2019; Sokmen et al. 2014).

**FIGURE 2:**
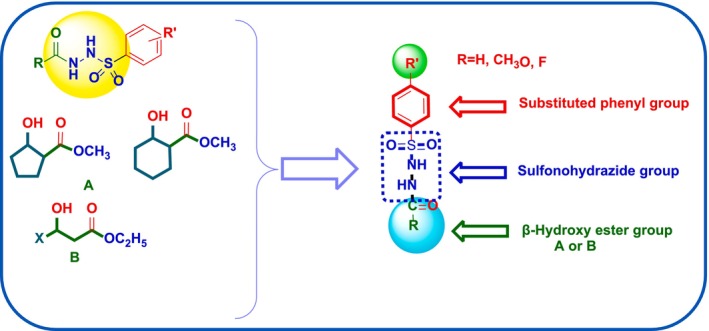
Design strategy of target compounds.

In the present work, a rational design strategy was developed to repurpose β‐hydroxy ester derivatives toward anticancer applications by incorporating the sulfonohydrazide moiety, a well‐established pharmacophore associated with anticancer activity. Accordingly, β‐hydroxy ester derivatives were transformed into their corresponding sulfonohydrazide analogs to generate a new series of compounds with potential anticancer properties (Figure 2). This design aimed to investigate systematically anticancer activity through structural variation. For this aim, a series of *N*‐acyl sulfonohydrazide compounds containing alicyclic, aromatic, and heteroaromatic groups were synthesized. This approach was designed to investigate the influence of structural modifications on biological activity and to establish preliminary structure–activity relationships (SAR) within the context of anticancer activity.

Overall, this study focuses on the rational modification of β‐hydroxy ester‐sulfonohydrazide hybrid derivatives to explore their potential as anticancer agents. Moreover, antioxidant and antimicrobial activities are included to support a broader understanding of their pharmacological profile.

## 3 Results and Discussion

### 3.1 Chemistry

**SCHEME 1:**
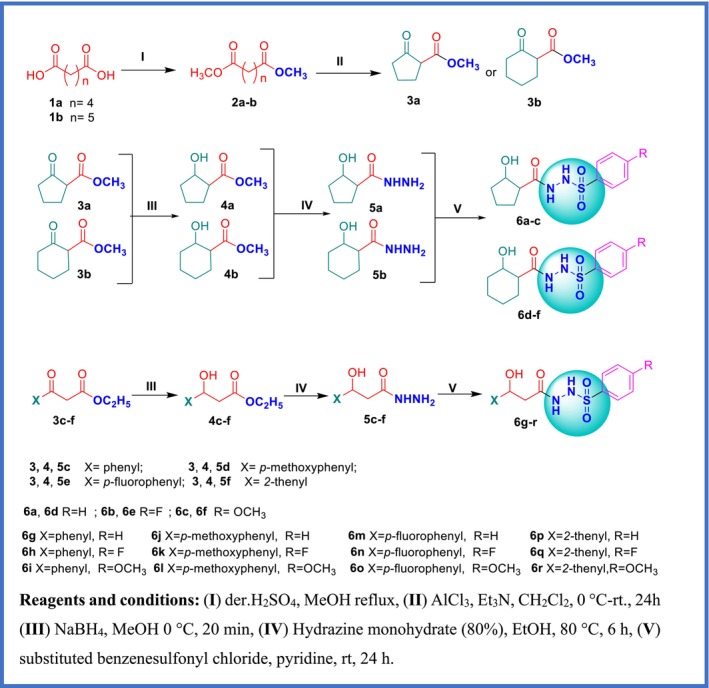
Synthetic route for the synthesis of new *N*‐acyl sulfonohydrazides **6a–r**.

Scheme 1 Illustrates the synthetic pathways employed for the preparation of precursor compounds **3a‐b** and the subsequent synthesis of novel sulfonohydrazides **6a–r**.

**FIGURE 3:**
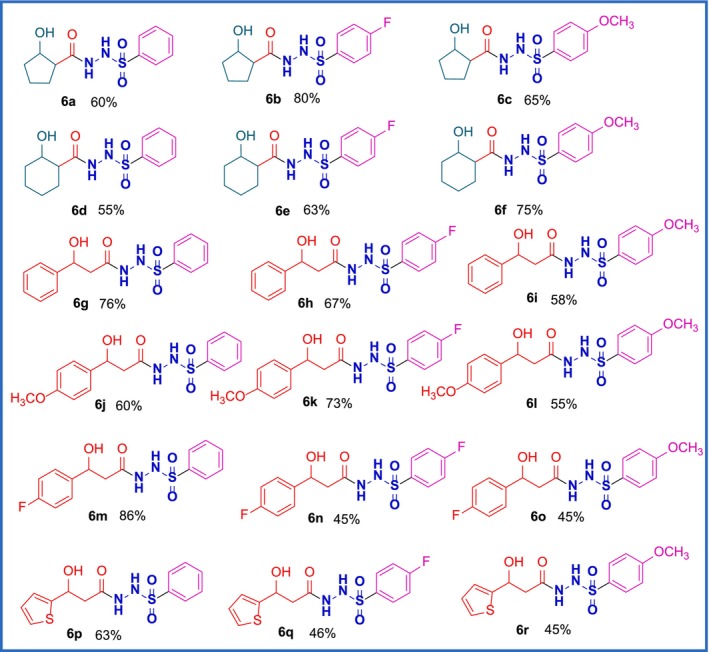
Structure of the target molecules.

Initially, compounds **1a‐b** were esterified to give dimethyl esters **2a‐b** in 95%–99% yield. Subsequently, Dieckmann cyclization of **2a‐b** afforded β‐keto esters **3a‐b** in 70%–75% yield. The Dieckmann reaction occurred with aluminum trichloride (AlCl_3_) and triethylamine (Et_3_N) as catalysts. In this reaction, triethylamine acts as a base to form the reactive enolate intermediate, while aluminum chloride acts as a Lewis acid to activate the ester carbonyl group. Thus, intramolecular nucleophilic attack facilitates efficient cyclization and β‐keto ester formation (Fraga et al. 2004). On the other hand, **3c–f** were purchased from commercial suppliers and used without further purification. Reduction of **3a–f** with NaBH₄ in methanol gave β‐hydroxy esters **4a–f** quantitatively (Hasdemir 2015; Hasdemir et al. 2012; Hasdemir and Yusufoğlu 2004). Following this, compounds **4a–f** were subjected to reflux conditions with 80% hydrazine monohydrate in ethanol at 80°C for 6 h, thereby preparing the hydrazide derivatives (**5a–f**) in moderate yields ranging from 50% to 70% (Scheme S1). Finally, the target *N*‐acyl sulfonohydrazides **6a–r** were obtained by *N*‐sulfonylation of hydrazide intermediates **5a–f** with various aromatic substituted (phenyl, *p*‐methoxyphenyl, and *p*‐fluorophenyl) sulfonyl chlorides. This transformation was carried out at room temperature for 24 h in the presence of pyridine, yielding an isolated yield of 45%–86% (Scheme S2). The structures of all target compounds **6a–r** are depicted in Figure 3.

Spectral data of the synthesized hydrazide compounds **5a–d** and **5f** are available in the literature (Bergmann and Goldschmidt 1968; Fülöp et al. 1990; Mamedov et al. 2005; Patrick et al. 1972), conversely, spectral data for compound **5e** are given for the first time in this study. IR spectra and LC–MS chromatograms of all hydrazide compounds **5a–f** were examined. In the IR spectra, characteristic peaks belonging to the –NHNH_2_ group were detected between 3300–3340 cm^−1^ (‐NH_2_) and 3278–3307 cm^−1^ (‐NH). In addition, the IR spectra of compounds showed the presence of ‐OH, C=O, C–N, and C–O stretching around 3136–3307, 1625–1647, 1207–1265, and 1074–1170 cm^−1^, respectively. [M^+^‐1] peaks were detected in the LC–MS chromatograms of the compounds. The spectrum results were found to agree with the literature data. In the ^1^H NMR spectrum of compound **5e**, peaks belonging to the –NH and –NH_2_ groups were observed as singlets at 8.94 and 4.15 ppm, respectively. The ^13^C NMR spectrum results also confirmed the structure of the compound.

The structures of the target compounds **6a–r** were characterized by IR, NMR, and HRMS analyses. In the IR spectra, characteristic absorption bands corresponding to N‐H stretching vibrations were observed at 3599–3307 cm^−1^, while O‐H stretching vibrations appeared at 3348–3167 cm^−1^. The C=O stretching bands were detected in the range of 1685–1645 cm^−1^. In addition, the asymmetric and symmetric stretching vibrations of the O=S=O group were seen at 1386–1301 and 1172–1149 cm^−1^, respectively.

In ^1^H NMR spectra, NH peaks were observed in the range of 10.02–9.87 and 9.90–9.56 ppm as a singlet. Aromatic CH peaks gave doublet or multiplet signals in the range of 8.91–6.82 ppm. The –CH proton to which the hydroxyl (‐OH) group was bonded gave chemical shift values in the forms of singlet (s), doublet of doublet (dd), and multiplet (m) around 5.05–3.81 ppm. The peaks for aliphatic methylene protons of cyclopentane and cyclohexane rings were observed at δ 2.47–2.37, 2.21–2.18, and 1.70–1.06 ppm, and methoxy protons at 3.89–3.15 ppm. The hydroxyl (OH) proton was seen as a singlet in the range of 5.30–5.67 ppm only in the spectra of compounds **6g**, **6k**, and **6l**.

In the ^13^C NMR spectra, the carbonyl group (C=O) peak is seen in the range of 173.38–168.76 ppm, and the peak belonging to the carbon atom (–CH–OH) to which the hydroxyl group is bonded is seen in the range of 73.60–65.76 ppm. The signals corresponding to the –OCH_3_ groups in the synthesized compounds were observed in the range of 56.09–55.49 ppm. The other peaks observed in the spectrum were consistent with their compound structures.

In their HRMS, the apparent absorption signals of [M]^+^, [M + H]^+^, and [M + Na]^+^ were in accordance with the corresponding molecular weights of compounds **6a–r**.

Details of the spectral data for hydrazides **5a–f** and new sulfonohydrazides **6a–r** are presented in the supporting information (Figures S1–56).

### 3.2 Biological Activity Studies

#### 3.2.1 Anticancer Activity

**FIGURE 4:**
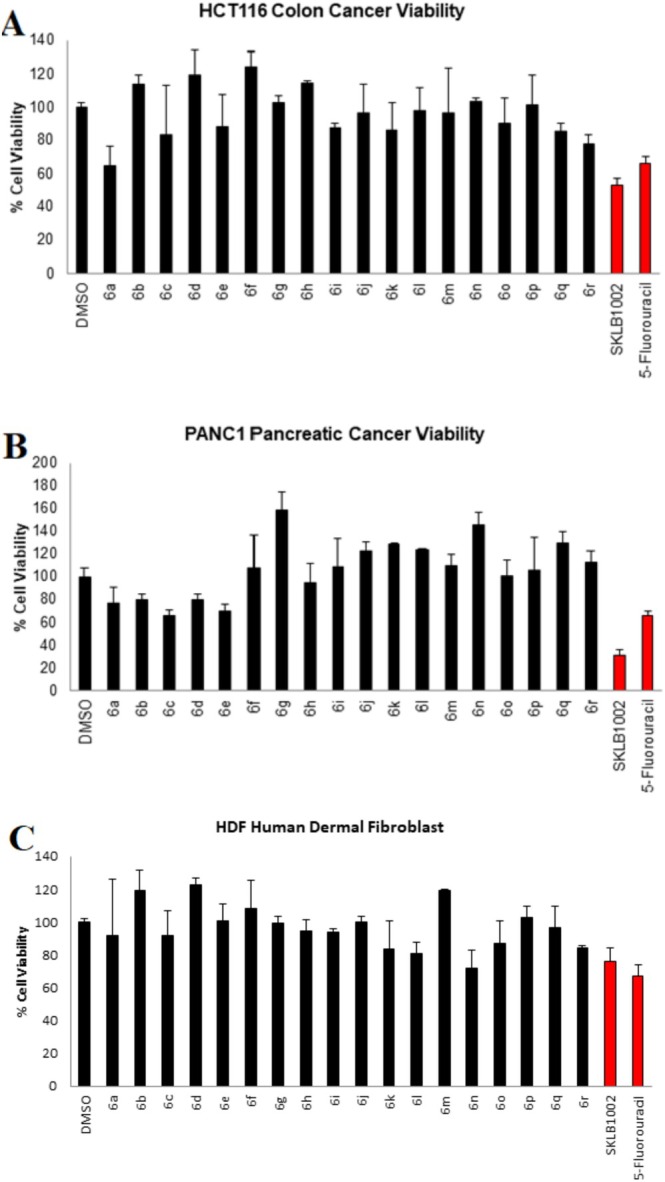
Assessment of cell viability after treatment with 20 μM doses (*p < 0.05).

In this study, the anticancer potential of *N*‐acyl sulfonohydrazide compounds **6a–r**, synthesized by the rational modification of β‐hydroxy ester skeletons, was investigated. Synthesized compounds **6a–r** were evaluated for their cytotoxicity activity against a range of cell lines, including HCT116 (colon carcinoma) (Figure 4A), PANC1 (pancreatic cancer) (Figure 4B), and HDF (human dermal fibroblast) (Figure 4C). These compounds **6a–r** were applied at a fixed concentration of 20 μM, with cellular viability subsequently quantified via the MTS assay. Among the tested compounds, compounds **6a–e** exhibited modest anti‐proliferative effects against HCT116 and PANC1 cancer cell lines, while no significant cytotoxicity was observed in normal HDF cells (Figure 4C), suggesting a degree of selectivity. In contrast, reference compounds SKLB1002 and 5‐Fluorouracil exerted substantial suppressive effects on colon and pancreatic cancers. In the original preclinical characterization by Zhang et al., SKLB1002 showed markedly lower cytotoxicity toward the normal human hepatocyte cell line L‐02, with a reported IC_50_ of 152.4 μM (Zhang et al. 2011). This value is orders of magnitude higher than the concentrations required for anti‐angiogenic activity, indicating a favorable selectivity for tumor‐associated endothelial cells over normal tissue. Our data in HDFs extend this safety profile to dermal fibroblasts, demonstrating that the low toxicity observed in L‐02 cells is also present in primary human skin fibroblasts. 5‐Fluorouracil (5‐FU) has been extensively evaluated in human dermal fibroblasts. However, 5‐Fluorouracil exhibited a degree of cytotoxicity against normal dermal fibroblasts, as evidenced by 67% viability, although this observation did not attain statistical significance. In the literature, multiple studies report that 5‐Fluorouracil exerts minimal cytotoxic effects on HDFs at clinically relevant concentrations (Alkış et al. 2021; Ell et al. 2024). These findings are consistent with the broader literature showing that 5‐FU exhibits selective toxicity toward rapidly dividing tumor cells while sparing normal fibroblasts, a property that underlies its use in both oncologic and dermatologic applications.

To comprehensively characterize their therapeutic potential, the half‐maximal inhibitory concentration (IC_50_) values for compounds **6a–e** were determined over a concentration range spanning from 0.01 to 100 μM. These compounds demonstrated cytotoxic properties against cancer cells, yet their IC_50_ values consistently exceeded 100 μM. This finding indicates markedly lower potency compared to the established benchmarks, SKLB1002 (10 μM) and 5‐Fluorouracil (42 μM), when assessed in PANC1 cells (see SI, Table S1). Given that the evaluated compounds failed to induce cytotoxicity against either HDF cells or HCT116 colon cancer cells at the concentrations employed, IC_50_ values could not be established for these specific cell lines.

##### 3.2.1.1 Apoptotic Effects of Compounds 6a–e

**FIGURE 5:**
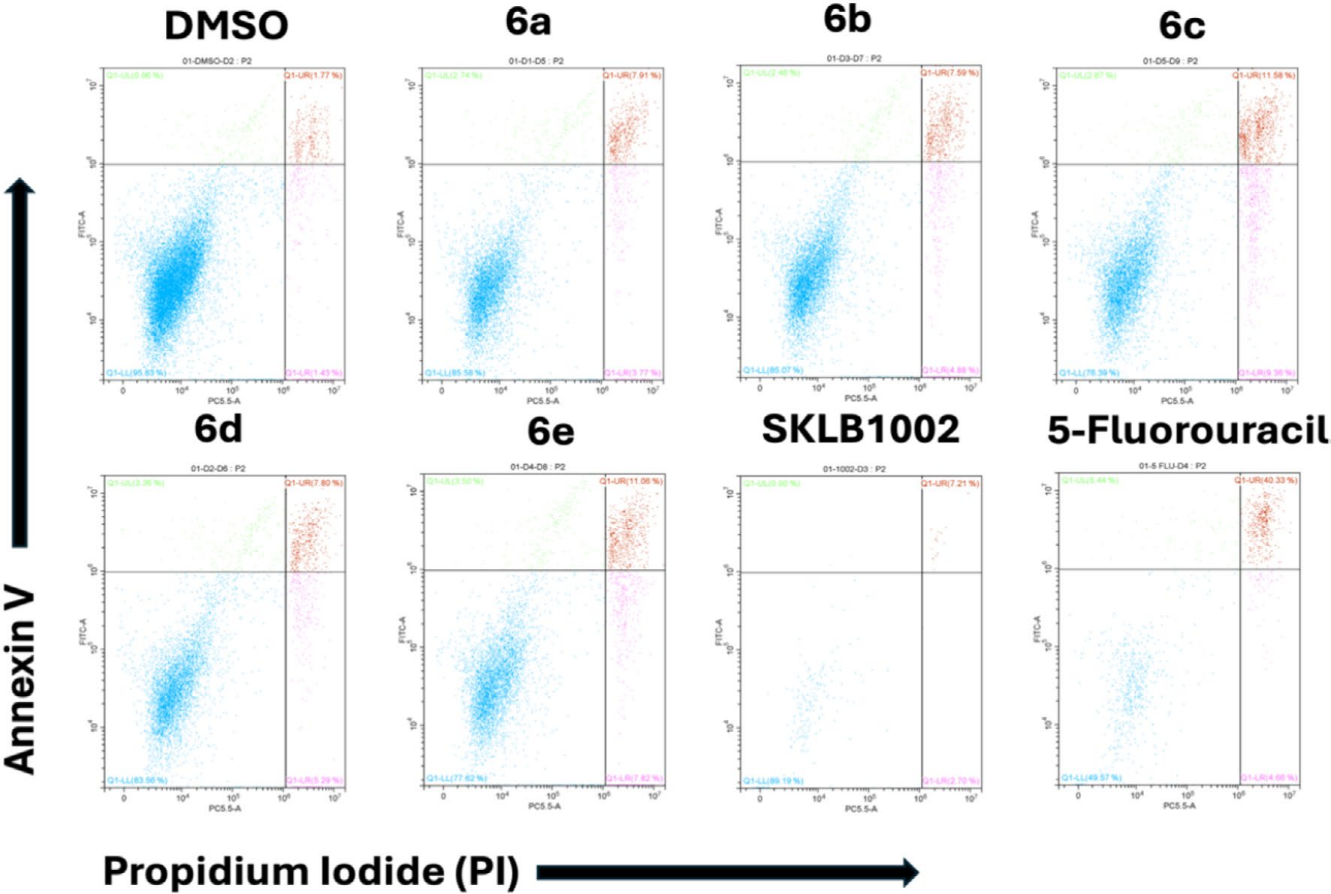
Representative gates of apoptosis for control as well as **6a–e** treatments (at 50 μM).

The pro‐apoptotic potential of compounds **6a–e** was further examined in HCT116 cellular models (Table 1 and Figure 5). Treatment at 50 μM resulted in varying levels of apoptosis compared to the vehicle control (DMSO). Among the tested compounds, derivatives 6c and 6e exhibited relatively higher apoptotic responses, with total apoptosis rates of 22.2% and 21.9%, respectively. These figures were comparable to the established reference angiogenesis inhibitor, SKLB1002 (21.7%). In contrast, compounds **6a**, **6b**, and **6d** showed lower activity, with apoptosis levels ranging between 12.8% and 15.1%. Notably, compounds 6c and 6e induced comparatively higher levels of late‐stage apoptosis (11.0% and 10.9%, respectively) as well as necrotic cell death when compared with the other analogs. Despite these observations, the overall apoptosis‐inducing effects of the tested compounds can be considered moderate. This is consistent with their relatively weak antiproliferative activity observed in cytotoxicity assays, indicating that apoptosis induction alone does not translate into strong anticancer efficacy in this series. In comparison, 5‐Fluorouracil exhibited the most pronounced effect, inducing 47.3% total apoptosis, predominantly through late‐stage apoptosis (38.6%). These observations, while compounds **6c** and **6e** demonstrate a measurable ability to induce apoptosis, should be interpreted as preliminary and supportive rather than definitive. The findings suggest that these derivatives may partially activate apoptotic pathways, but further structural optimization is required to enhance their overall cytotoxic and anticancer potential.

**TABLE 1:** Analysis of apoptosis for **6a–e** compounds^a^.

Treatment	Late apoptosis	Early apoptosis	Necrotic	Total apoptotic
DMSO	1.9 ± 0.2	1.0 ± 0.1	1.5 ± 0.1	4.4 ± 0.3
**6a**	6.9 ± 1.4	2.4 ± 0.5	3.5 ± 0.3	12.8 ± 2.2*
**6b**	7.1 ± 0.7*	2.6 ± 0.1*	4.6 ± 0.4*	14.3 ± 0.9
**6c**	11.0 ± 0.8*	2.6 ± 0.1*	8.6 ± 1.1	22.2 ± 2.0
**6d**	7.2 ± 0.8	3.1 ± 0.4	4.8 ± 0.7	15.1 ± 1.9
**6e**	10.9 ± 0.2*	3.4 ± 0.1*	7.5 ± 0.4*	21.9 ± 0.7
SKLB1002	14.8 ± 10.8	3.5 ± 3.7	3.4 ± 3.8	21.7 ± 15.4
5‐Fluorouracil	38.6 ± 2.5*	5.0 ± 0.7	3.7 ± 1.3	47.3 ± 4.5

^a^ Results were provided as Mean ± SDev.

* *p* < 0.01 in comparison to the DMSO control group.

#### 3.2.2 Antioxidant Activity

The antioxidant capacities of the synthesized *N*‐acyl sulfonohydrazides **6a–r** were evaluated to provide complementary insight into their overall biological profile, particularly in relation to oxidative stress processes associated with cancer. Oxidative stress, mediated by reactive oxygen species (ROS), is known to contribute to cancer development and progression, and modulation of redox balance may influence cellular responses to anticancer agents. Therefore, antioxidant activity was assessed as a supporting parameter rather than a primary therapeutic objective.

The antioxidant capacities of the synthesized compounds **6a–r** were assessed using the CUPRAC spectrophotometric method, with trolox serving as a reference standard, at ambient temperature (Apak et al. 2004). The molar absorption coefficient (ε) for each compound was determined from the slope of its linear calibration curve, which plots absorbance against molar concentration in a 1‐cm cuvette. The Trolox Equivalent Antioxidant Capacity (TEAC), or TEAC coefficient, reflecting the antioxidant potency, was subsequently calculated by dividing the CUPRAC molar absorption coefficient of each test compound by that of trolox, under identical experimental parameters. As shown in Table 2, all compounds exhibited moderate to considerable antioxidant capacity, with TEAC values ranging from 0.68 to 1.34. Among them, compound **6l** displayed the highest antioxidant capacity, while compound **6h** showed the lowest activity. The relative antioxidant ranking based on CUPRAC‐TEAC values followed the order: **6l** > **6c** > **6a** > **6i** > **6j** > **6k** > **6d** > **6e** > **6g** > **6m** > **6f** > **6o** > **6q** > **6n** > **6r** > **6p** > **6b** > **6h**.

**TABLE 2:** Calibration equations, linear ranges, and TEAC coefficients of the synthesized compounds according to the CUPRAC method^a^ and IC_50_ values according to the DPPH method^b^.

CUPRAC assay					DPPH assay
Compounds	Linear range (mol L^−1^)	Calibration equation	*r*	TEAC	IC_50_ (mmoL L^−1^)
**6a**	6.01 × 10^−6^—6.01 × 10^−5^	*y* = 17,867 *c* + 0.0494	0.9974	1.17	2.575 ± 0.226
**6b**		*y* = 12,870 *c* + 0.0145	0.9997	0.85	3.107 ± 0.038
**6c**		*y* = 18,075 *c* + 0.0097	0.9985	1.19	7.096 ± 0.045
**6d**		*y* = 16,619 *c* + 0.0428	0.9979	1.09	2.311 ± 0.052
**6e**		*y* = 16,511 *c* + 0.0285	0.9997	1.08	6.560 ± 0.097
**6f**		*y* = 15,604 *c* + 0.0302	0.9991	1.03	2.826 ± 0.063
**6g**		*y* = 16,317 *c* + 0.0375	0.9990	1.07	4.399 ± 0.168
**6h**		*y* = 10,360 *c* + 0.0136	0.9995	0.68	3.589 ± 0.014
**6i**		*y* = 17,424 *c* + 0.0149	0.9992	1.14	9.249 ± 0.182
**6j**		*y* = 17,005 *c* + 0.0466	0.9987	1.12	9.639 ± 0.097
**6k**		*y* = 16,934 *c* + 0.0172	0.9992	1.11	7.812 ± 0.090
**6l**		*y* = 20,448 *c* + 0.0002	0.9999	**1.34**	**0.084 ± 0.003**
**6m**		*y* = 15,859 *c* + 0.0447	0.9980	1.04	1.815 ± 0.012
**6n**		*y* = 13,913 *c* + 0.0198	0.9993	0.91	4.662 ± 0.041
**6o**		*y* = 15,533 *c* + 0.0298	0.9987	1.02	3.683 ± 0.003
**6p**		*y* = 13,037 *c* + 0.0235	0.9994	0.86	5.365 ± 0.087
**6q**		*y* = 14,329 *c* + 0.0174	0.9974	0.94	3.982 ± 0.053
**6r**		*y* = 13,673 *c* + 0.0222	0.9982	0.90	2.046 ± 0.011
TR^c^					0.002 ± 0.000011
AA^c^					0.002 ± 0.000091
BHA^c^					3.029 ± 0.013
BHT^c^					6.894 ± 0.133

^a^ TEAC_AOX_ = ε_AOX_/ε_TR_ (AOX antioxidant, TR Trolox); ε_TR_ = 1.52 × 10^4^ L^−1^ mol^−1^ cm^−1^ (DMSO).

^b^ Results are shown as mean ± standard deviation (*n* = 3).

^c^ Reference compounds: AA = ascorbic acid, BHA = butylated hydroxyanisole, BHT = butylated hydroxytoluene, TR = trolox.

In addition, the free radical scavenging activity of the compounds was evaluated using the DPPH assay, which measures the ability of compounds to neutralize stable free radicals via hydrogen atom donation (Sztanke and Sztanke 2017). The results were expressed as IC_50_ values (Table 2). The IC_50_ value denotes the concentration required to achieve 50% inhibition of DPPH radicals. Ascorbic acid (AA), BHA, and BHT were employed as reference standards. Compound **6l** exhibited the most potent activity (IC_50_ = 0.084 ± 0.003 mmol L^−1^), indicating strong radical scavenging ability, although still lower than that of ascorbic acid (IC_50_ = 0.002 ± 0.000091 mmol L^−1^). Compounds **6a**, **6d**, **6f**, **6m**, and **6r** also demonstrated notable antioxidant activity, exceeding that of BHA and BHT under the tested conditions. Other derivatives showed moderate to low activity. The overall activity trend was observed as follows:

**6l** > **6m** > **6r** > **6d** > **6a** > **6f** > **6b** > **6h** > **6o** > **6q** > **6g** > **6n** > **6p** > **6e** > **6c** > **6k** > **6i** > **6j**.

Overall, these findings suggest that the synthesized sulfonohydrazide derivatives possess varying degrees of antioxidant capacity. While not the primary focus of the study, these properties may contribute to the modulation of oxidative stress‐related pathways and support the broader biological evaluation of the compounds in the context of anticancer research.

#### 3.2.3 Antimicrobial Activity

The synthesized *N*‐acyl sulfonohydrazide derivatives **6a–r** were evaluated in vitro for their antibacterial and antifungal activities against a panel of Gram‐negative bacteria ( *Pseudomonas aeruginosa* ATCC 27853, *Escherichia coli* ATCC 25922, *Klebsiella pneumoniae* ATCC 4352, and *Proteus mirabilis* ATCC 14153), Gram‐positive bacteria ( *Staphylococcus aureus* ATCC 29213, *Staphylococcus epidermidis* ATCC 12228, *Enterococcus faecalis* ATCC 29212, and Methicillin‐resistant *Staphylococcus aureus* [MRSA] ATCC 43300), and *Candida* species ( *Candida albicans* ATCC 10231, *Candida parapsilosis* ATCC 22019, and *Candida tropicalis* ATCC 750). According to accepted antimicrobial activity criteria, compounds exhibiting MIC values > 32 μg/mL are generally considered inactive. Overall, the majority of the synthesized compounds demonstrated limited or no clinically significant antimicrobial activity, as most MIC values exceeded this threshold (see SI, Tables S2 and S3). Against Gram‐negative bacteria, including *E. coli* , *K. pneumoniae* , and *P. mirabilis* , all compounds were considered inactive (MIC > 32 μg/mL). Similarly, activity against *P. aeruginosa* was weak, with compounds **6h**, **6n**, and **6o** showing only low‐level inhibition (MIC = 312.5 μg/mL), which remains well above the threshold for meaningful antibacterial potency. For Gram‐positive organisms, selected compounds displayed comparatively improved but still moderate activity. Compounds **6a**, **6b**, and **6d** exhibited the most notable antibacterial effects against *S. aureus* , with MIC values of 19.53 μg/mL, indicating measurable but modest antibacterial potential. Several additional compounds showed weaker inhibitory effects, while the overall activity against methicillin‐resistant *S. aureus* (MRSA) and *E. faecalis* remained limited or inactive.

Antifungal testing against *C. albicans* , *C. parapsilosis* , and *C. tropicalis* similarly revealed generally weak activity. Although some derivatives demonstrated low to moderate inhibition, particularly against *C. tropicalis* , the majority of MIC values remained above the accepted threshold for significant antifungal activity. Therefore, these compounds cannot be considered potent antifungal agents under the tested conditions.

Importantly, in this study, these sulfonohydrazide derivatives were not specifically designed as classical sulfonamide‐based antimicrobial agents targeting dihydropteroate synthase. Rather, antimicrobial screening was included as part of a broader biological profiling strategy to assess potential auxiliary pharmacological properties. Consequently, the observed antimicrobial findings should be interpreted as exploratory rather than mechanism‐driven. Furthermore, no mechanistic conclusions regarding bacterial resistance or sulfonamide‐like activity can be drawn from the current dataset. Overall, while certain derivatives displayed modest activity against selected Gram‐positive bacteria, the antibacterial and antifungal profiles of the synthesized compounds were generally limited. These findings suggest that the primary therapeutic potential of this compound series may lie outside antimicrobial applications, reinforcing the study's principal emphasis on anticancer evaluation.

#### 3.2.4 Structure–Activity Relationship (SAR)

**FIGURE 6:**
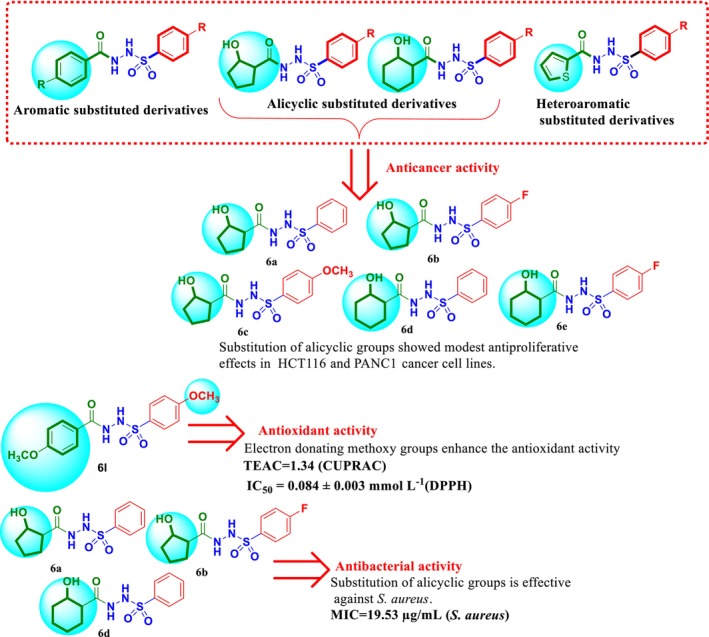
Structure–activity relationship overview of sulfonohydrazides **6a–e** and **6l**.

To investigate the effect of structural diversity on the biological activities of the synthesized *N*‐acyl sulfonohydrazide derivatives, a structure–activity relationship (SAR) analysis was performed, referencing the structural features and corresponding activity data shown in Figure 6. As shown in Figure 6, the synthesized compounds can be generally classified as (i) alicyclic substituted derivatives (**6a–f**), (ii) aromatic substituted derivatives (**6g–o**), and (iii) heteroaromatic substituted derivatives (**6p–r**) according to the nature of the *N*‐acyl substituent. This structural classification provides a useful framework for relating molecular properties to biological activity.

In terms of anticancer activity, compounds **6a–e**, containing alicyclic moieties as shown in Figure 6, demonstrated measurable cytotoxic effects compared to other derivatives. However, relatively high IC_50_ values indicate that these effects remain modest. In particular, compounds **6c** and **6e**, although belonging to the same structural class but differing in their substituent group, exhibited relatively higher apoptotic activity, further highlighting that subtle structural differences can significantly influence the biological response. These findings underscore the limitations of drawing definitive SAR conclusions from a relatively small and structurally diverse set of compounds.

When the antioxidant activity results are analyzed together with the structures in Figure 6, compound **6l**, which contains methoxy substituents in aromatic rings, shows the highest activity in both CUPRAC and DPPH tests. The presence of these methoxy groups appears to increase electron‐donating capacity and facilitate radical stabilization. However, when compared with other structurally related compounds, it shows that the antioxidant activity is influenced not only by electronic effects but also by factors such as conjugation length, substituent orientation, and steric accessibility.

With respect to their antibacterial properties, compounds containing alicyclic *N*‐acyl groups, such as **6a**, **6b**, and **6d**, showed relatively better activity against *S. aureus* . As shown in Figure 6, these compounds possess compact, alicyclic moieties that may facilitate better interaction with bacterial membranes or improve cellular uptake. Conversely, derivatives incorporating aromatic or heteroaromatic moieties demonstrated diminished efficacy. Nonetheless, this trend was not uniformly observed across all derivatives, suggesting that additional parameters, such as substituent location, steric bulk, and molecular hydrophobicity, contributed to the observed divergences in activity.

Overall, SAR analysis indicates that the biological activity in this series is governed by a complex interplay of structural and physicochemical factors, including substituent type, location, molecular size, and flexibility.

## 4 Conclusion

In summary, 18 novel *N*‐acyl sulfonohydrazide derivatives **6a–r** were designed and synthesized from β‐hydroxy ester precursors to develop new compounds with potential anticancer activity. The biological evaluation was primarily focused on anticancer properties, while antioxidant and antimicrobial activities were assessed as complementary parameters to provide a broader understanding of the compounds' biological profiles. In the cytotoxicity studies, compounds **6a–e** demonstrated selective anti‐proliferative effects against HCT116 and PANC1 cancer cell lines, with minimal toxicity toward normal HDF cells. Although their IC_50_ values indicate moderate potency, apoptosis analyses revealed that compounds **6c** and **6e** induced higher levels of late‐stage apoptosis and necrosis compared to other derivatives. In this context, these compounds may serve as promising lead structures for further optimization aimed at improving potency and selectivity. Antioxidant tests (CUPRAC and DPPH) revealed that many compounds, especially **6l**, possessed significant antioxidant capacity. In contrast, antimicrobial evaluation showed that the tested compounds exhibited moderate and selective activity against Gram‐positive bacteria.

Overall, this work highlights the transformation of β‐hydroxy ester scaffolds into sulfonohydrazide derivatives with measurable anticancer potential. The findings may provide a basis for future structural optimization and mechanistic studies directed toward the development of more potent anticancer agents.

## 5 Experimental Section

### 5.1 Chemistry

Detailed experimental procedures for the synthesis of intermediate and target compounds, as well as data on molecular docking studies (Tables S4–S6 and Figures S57–S59), are provided in the SI.

### 5.2 Analytical Data for the Hydrazide Compounds 5a–f

#### 5.2.1 2‐Hydroxycyclopentanecarbohydrazide 5a

White solid, Yield 50%, mp: 156.4°C–157.7°C (152°C–154°C (Fülöp et al. 1990)), **IR (ATR) υ**_**max**_ **/cm** ^**−1**^: 3330, 3300, 3136, 2931, 2864, 1635, 1261, 1101. **LC–MS (m/z):** Calc. for C_6_H_12_N_2_O_2_: 144, Found 143 (M^+^‐1).

#### 5.2.2 2‐Hydroxycyclohexanecarbohydrazide 5b

White solid, Yield 60%, mp: 175°C–176.5°C (207°C–208°C (Fülöp et al. 1990)), **IR (ATR) υ**_**max**_ **/cm** ^**−1**^: 3330, 3288, 3196, 2926, 2850, 1625, 1265, 1078. **LC–MS (m/z):** Calc. for C_7_H_14_N_2_O_2_; 158. Found 157 (M^+^‐1).

#### 5.2.3 3‐Hydroxy‐3‐Phenylpropanehydrazide 5c

White solid, Yield 65%, mp: 160.2°C–160.8°C (160°C–162°C (Mamedov et al. 2005)), **IR (ATR) υ**_**max**_ **/cm** ^**−1**^: 3320, 3278, 3203, 3030, 2856, 1647, 1618, 1539, 1211, 1085. **LC–MS (m/z):** Calc. for C_9_H_12_N_2_O_2_; 180. Found 179 (M^+^‐1).

#### 5.2.4 3‐Hydroxy‐3‐(4‐Methoxyphenyl)Propanehydrazide 5d

White solid, Yield 50%, mp: 174°C–174.2°C (177°C (Bergmann and Sulzbacher 1951)), **IR (ATR) υ**_**max**_ **/cm** ^**−1**^: 3300, 3196, 3059, 2835, 1641, 1612, 1508, 1240, 1170. **LC–MS (m/z):** Calc. for C_10_H_14_N_2_O_3_; 210. Found 209 (M^+^‐1).

#### 5.2.5 3‐(4‐Fluorophenyl)‐3‐Hydroxypropanehydrazide 5e

White solid, Yield 70%, mp: 157.3°C–158.3°C (180°C (Bergmann and Goldschmidt 1968)), **IR (ATR) υ**_**max**_ **/cm** ^**−1**^: 3340, 3302, 3155, 3050, 2902, 1635, 1604, 1508, 1207, 1155. ^**1**^ **H NMR (400 MHz, DMSO)** δ: 8.94 (s, 1H, NH), 7.36–7.33 (m, 2H, Ar‐H), 7.14–7.10 (m, 2H, Ar‐H), 5.40 (d, *J* = 4.3 Hz, 1H), 4.95 (br s, 1H, OH), 4.15 (s, 2H, NH_2_), 2.37 (dd, *J*_*1*_ = 14.0, *J*_*2*_ = 8.5 Hz, 1H), 2.28 (dd, *J*_*1*_ = 14.0, *J*_*2*_ = 5.1 Hz, 1H). ^**13**^ **C NMR (101 MHz, DMSO)** δ: 169.72, 162.82 (d, ^*1*^ *J*_*CF*_ = 243.1 Hz), 160.41 (d, ^*1*^ *J*_*CF*_ = 243.1 Hz), 141.94, 128.09 21 (d, ^*3*^ *J*_*CF*_ = 8.0 Hz), 128.01 21 (d, ^*3*^ *J*_*CF*_ = 8.0 Hz), 115.26 (d, ^*2*^ *J*_*CF*_ = 21.0 Hz), 115.05 (d, ^*2*^ *J*_*CF*_ = 21.0 Hz), 69.36, 44.33. **LC–MS (m/z):** Calc. for C_9_H_11_FN_2_O_2_; 198. Found 197 (M^+^‐1).

#### 5.2.6 3‐Hydroxy‐3‐(Thiophen‐2‐Yl)Propanehydrazide 5f

White solid, Yield 55%, mp: 138.5°C–139°C (139°C–140°C (Patrick et al. 1972)), **IR (ATR) υ**_**max**_ **/cm** ^**−1**^: 3307, 3209, 3064, 2904, 2837, 1635, 1614, 1531, 1249, 1074. **LC–MS (m/z):** Calc. for C_7_H_10_N_2_O_2_S; 186. Found 185 (M^+^‐1).

### 5.3 Analytical Data for the Target Compounds 6a‐r

#### 5.3.1 N′‐2‐Hydroxycyclopentanoylbenzenesulfonohydrazide 6a

White solid, Yield 60%, mp: 130.8°C–131.8°C, **IR (ATR) υ**_**max**_ **/cm** ^**−1**^: 3317 (NH), 3191 (OH), 3066 (aromatic CH), 2812 (aliphatic CH), 1660 (C=O), 1589 (aromatic C=C), 1334, 1162 (SO_2_ , asymmetric and symmetric vibrations), 1087 (C‐O). ^**1**^ **H** **NMR (400 MHz, DMSO)** δ: 9.87 (s, 1H, NH), 9.77 (s, 1H, NH), 7.82 (d, *J* = 6.4 Hz, 2H, Ar‐H), 7.62 (d, *J* = 6.6 Hz, 1H, Ar‐H), 7.54 (d, *J* = 6.9 Hz, 2H, Ar‐H), 4.35 (s, 1H), 2.47–2.37 (m, 1H), 1.67–1.43 (m, 6H). ^**13**^ **C** **NMR (101 MHz, DMSO)** δ: 172.24 (C=O), 139.34, 133.34, 129.18, 128.16, 73.60 (C‐OH), 48.06, 34.69, 25.97, 21.67. **HRMS (ESI) m/z:** Calc. for C_12_H_16_N_2_O_4_S 284.3314, Found 284.0799.

#### 5.3.2 4‐Fluoro‐N′‐2‐Hydroxycyclopentanoylbenzenesulfonohydrazide 6b

White solid, Yield 80%, mp: 149.6°C–151°C, **IR (ATR) υ**_**max**_ **/cm** ^**−1**^: 3380 (NH), 3190 (OH), 3012 (aromatic CH), 2941, 2816 (aliphatic CH), 1685 (C=O), 1587 (aromatic C=C), 1344, 1168 (SO_2_, asymmetric and symmetric vibrations), 1087 (C–O). ^**1**^ **H NMR (400 MHz, DMSO)** δ: 9.93 (s, 1H, NH), 9.84 (s, 1H, NH), 7.87–7.84 (m, 2H, Ar‐H), 7.38–7.34 (m, 2H, Ar‐H), 4.37 (s, 1H), 2.42–2.37 (m, 1H), 1.70–1.42 (m, 6H). ^**13**^ **C NMR (101 MHz, DMSO)** δ: 172.02 (C=O), 165.00 (d, ^1^ *J*_CF_ = 250.8 Hz), 135.70, 131.33 (d, ^3^ *J*_CF_ = 9.7 Hz), 116.29 (d, ^2^ *J*_CF_ = 22.7 Hz), 73.61 (C‐OH), 48.13, 34.77, 25.65, 21.51. **HRMS (ESI) m/z**: Calc. for C_12_H_15_FN_2_O_4_S 302.3219, Found 303.0814 (M + H)^+^, 325.0565 (M + Na)^+^.

#### 5.3.3 4‐Methoxy‐N′‐2‐Hydroxycyclopentanoylbenzenesulfonohydrazide 6c

White solid, Yield 65%, mp: 151.8°C–152.7°C, **IR (ATR) υ**_**max**_ **/cm** ^**−1**^: 3489 (NH), 3190 (OH), 3053 (aromatic CH), 2889 (aliphatic CH), 1670 (C=O), 1593 (aromatic C=C), 1338, 1161 (SO_2_, asymmetric and symmetric vibrations), 1089 (C–O). ^**1**^ **H NMR (400 MHz, DMSO)** δ: 9.87 (s, 1H, NH), 9.56 (s, 1H, NH), 7.73 (d, *J* = 8.1 Hz, 2H, Ar‐H), 7.05 (d, *J* = 8.0 Hz, 2H, Ar‐H), 4.34 (s, 1H), 3.89 (s, 3H), 2.39 (s, 1H), 1.68–1.44 (m, 6H).^**13**^ **C NMR (101 MHz, DMSO)** δ: 172.05 (C=O), 163.02, 130.78, 130.45, 114.38, 73.60 (C‐OH), 56.09 (OCH_3_), 48.11, 34.73, 26.01, 21.75. **HRMS (ESI) m/z**: Calc. for C_13_H_18_N_2_O_5_S 314.3574, Found 314.6855, 315.1017 (M + H)^+^, 337.0850 (M + Na)^+^.

#### 5.3.4 N′‐2‐Hydroxycyclohexanoylbenzenesulfonohydrazide 6d

White solid, Yield 55%, mp: 154.6.8°C–155.6°C, **IR (ATR) υ**_**max**_ **/cm** ^**−1**^: 3329 (NH), 3205 (OH), 3020 (aromatic CH), 2927 (aliphatic CH), 1668 (C=O), 1539 (aromatic C=C), 1348, 1172 (SO_2_, asymmetric and symmetric vibrations), 1055 (C–O). ^**1**^ **H NMR (400 MHz, DMSO)** δ: 9.88 (s, 1H, NH), 9.71 (s, 1H, NH), 7.80 (d, *J* = 7.4 Hz, 2H, Ar‐H), 7.63–7.59 (m, 1H, Ar‐H), 7.53–7.49 (m, 2H, Ar‐H), 4.47 (s, 1H), 1.93 (s, 1H), 1.60–1.48 (m, 4H), 1.10–1.01 (m, 4H).^**13**^ **C NMR (101 MHz, DMSO)** δ: 172.24 (C=O), 139.34, 133.34, 129.18, 128.16, 73.60 (C‐OH), 48.06, 34.69, 25.97, 21.67. **HRMS (ESI) m/z:** Calc. for C_13_H_18_N_2_O_4_S 298.358, Found 298.0935.

#### 5.3.5 4‐Fluoro‐N′‐2‐Hydroxycyclohexanoylbenzenesulfonohydrazide 6e

White solid, Yield 63%, mp: 156.2°C–157.8°C, **IR (ATR) υ**_**max**_ **/cm** ^**−1**^: 3433 (NH), 3169 (OH), 3045 (aromatic CH), 2931, 2854 (aliphatic CH), 1645 (C=O), 1591 (aromatic C=C), 1323, 1172 (SO_2_, asymmetric and symmetric vibrations), 1085 (C–O). ^**1**^ **H NMR (400 MHz, DMSO)** δ: 9.95 (s, 1H), 9.87 (s, 1H), 7.86–7.83 (m, 2H), 7.39–7.35 (m, 2H), 3.81 (s, 1H), 2.21–2.17 (m, 1H), 1.61–1.48 (m, 4H), 1.35–1.23 (m, 4H). ^**13**^ **C NMR (101 MHz, DMSO)** δ: 173.38 (C=O), 165.04 (d, ^1^ *J*_*CF*_ = 251.0 Hz), 135.48, 131.36 (d, ^3^ *J*_*CF*_ = 9.7 Hz), 116.34 (d, ^2^ *J*_CF_ = 22.8 Hz), 66.82 (C‐OH), 45.18, 32.12, 24.04, 24.00, 20.50. **HRMS (ESI) m/z**: Calc. for C_13_H_17_FN_2_O_4_S 316.3485, Found 317.0972 (M + H)^**+**^, 339.0789 (M + Na)^+^.

#### 5.3.6 4‐Methoxy‐N′‐2‐Hydroxycyclohexanoylbenzenesulfonohydrazide 6f

White solid, Yield 75%, mp: 142.5°C–144.8°C, **IR (ATR) υ**_**max**_ **/cm** ^**−1**^: 3307 (NH), 3176 (OH), 3043 (aromatic CH), 2927 (aliphatic CH), 1654 (C=O), 1595 (aromatic C=C), 1340, 1155 (SO_2_, asymmetric and symmetric vibrations), 1089 (C–O). ^**1**^ **H NMR (400 MHz, DMSO)** δ: 9.87 (s, 1H, NH), 9.56 (s, 1H, NH), 7.73 (d, *J* = 8.1 Hz, 2H, Ar‐H), 7.05 (d, *J* = 8.0 Hz, 2H, AR‐H), 4.34 (s, 1H), 3.89 (s, 3H), 2.21–2.18 (m, 1H), 1.68–1.44 (m, 6H).^**13**^ **C NMR (101 MHz, DMSO)** δ: 172.05 (C=O), 163.02, 130.78, 130.45, 114.38, 73.60 (C‐OH), 56.09 (OCH_3_), 48.11, 34.73, 26.01, 21.75. **HRMS (ESI) m/z:** Calc. for C_14_H_20_N_2_O_5_S 328.384, Found 329.1171 (M + H)^+^, 351.0995 (M + Na)^+^.

#### 5.3.7 N′‐3‐Hydroxy‐3‐Phenylpropanoylbenzenesulfonohydrazide 6g

Light brown solid, Yield 76%, mp: 114°C–115°C, **IR (ATR) υ**_**max**_ **/cm** ^**−1**^: 3599 (NH), 3327 (OH), 3082 (aromatic CH), 2933, 2814 (aliphatic CH), 1658 (C=O), 1537 (aromatic C=C), 1332, 1155 (SO_2_, asymmetric and symmetric vibrations), 1087 (C–O). ^**1**^ **H NMR (400 MHz, DMSO)** δ: 9.98 (s, 1H, NH), 9.84 (s, 1H, NH), 7.77–7.27 (m, 10H, Ar‐H), 5.30 (s, 1H), 4.80 (s, 1H), 2.44–2.34 (m, 1H), 2.29–2.16 (m, 1H). ^**13**^ **C NMR (101 MHz, DMSO)** δ: 169.16 (C=O), 145.46, 139.62, 133.28, 129.22, 128.50, 128.00, 127.40, 126.18, 69.70 (C‐OH), 43.73 (CH_2_). **HRMS (ESI) m/z:** Calc. for C_15_H_16_N_2_O_4_S 320.3635, Found: 321.0911 (M + H)^+^.

#### 5.3.8 4‐Fluoro‐N′‐3‐Hydroxy‐3‐Phenylpropanoyl Benzenesulfonohydrazide 6h

Beige solid, Yield 67%, mp: 110.8°C–111.2°C, **IR (ATR) υ**_**max**_ **/cm** ^**−1**^: 3500 (NH), 3342 (OH), 3068 (aromatic CH), 2810 (aliphatic CH), 1660 (C=O), 1591 (aromatic C=C), 1332, 1153 (SO_2_ , asymmetric and symmetric vibrations), 1087 (C–O). ^**1**^ **H** **NMR (400 MHz, DMSO)** δ: 10.03 (s, 1H, NH), 9.89 (s, 1H, NH), 8.91 (d, *J* = 5.0 Hz, 1H, Ar‐H), 8.04 (t, *J* = 6.9 Hz, 1H, Ar‐H), 7.81 (dd, *J* = 8.5, 5.2 Hz, 2H, Ar‐H), 7.37–7.23 (m, 5H), 4.79 (dd, *J*_*1*_ = 7.9, *J*_*2*_ = 5.1 Hz, 1H), 2.39 (dd, *J*_*1*_ = 14.2, *J*_*2*_ = 8.5 Hz, 1H), 2.27 (dd, *J*_*1*_ = 14.3, *J*_*2*_ = 5.0 Hz, 1H). ^**13**^ **C** **NMR (101 MHz, DMSO)** δ: 169.21 (C=O), 164.296 (d, ^*1*^ *J*_*CF*_ = 250.9 Hz), 145.95, 145.42, 142.95, 135.93, 1315.10 (d, ^*3*^ *J*_*CF*_ = 9.7 Hz), 128.50, 127.47, 127.42, 126.20, 116.38 (d, ^*2*^ *J*_*CF*_ = 22.6 Hz), 69.75 (C‐OH), 43.71 (CH_2_ ). **HRMS (ESI) m/z:** Calc. for C_15_H_15_FN_2_O_4_S 338.0737, Found 338.0716.

#### 5.3.9 4‐Methoxy‐N′‐3‐Hydroxy‐3‐Phenylpropanoyl Benzenesulfonohydrazide 6i

Beige solid, Yield 58%, mp: 127.8°C–130.7°C, **IR (ATR) υ**_**max**_ **/cm** ^**−1**^: 3322 (NH), 3191 (OH), 3065 (aromatic CH), 2923, 2853 (aliphatic CH), 1641 (C=O), 1596 (aromatic C=C), 1386, 1167 (SO_2_ , asymmetric and symmetric vibrations), 1092 (C–O). ^**1**^ **H** **NMR (400 MHz, DMSO)** δ: 9.93 (s, 1H, NH), 9.61 (s, 1H, NH), 7.67 (d, *J* = 8.8 Hz, 2H, Ar‐H), 7.31–7.20 (m, 5H, Ar‐H), 7.03 (d, *J* = 8.8 Hz, 2H, Ar‐H), 4.79 (dd, *J*_*1*_ = 7.8, *J*_*2*_ = 5.3 Hz, 1H), 3.15 (s, 3H), 2.38 (dd, *J*_*1*_ = 14.2, *J*_*2*_ = 8.4 Hz, 1H), 2.25 (dd, *J*_*1*_ = 14.3, *J*_*2*_ = 5.1 Hz, 1H). ^**13**^ **C** **NMR (101 MHz, DMSO)** δ: 169.07 (C=O), 163.00, 145.42, 131.01, 130.29, 128.49, 127.41, 126.19, 114.41, 69.72 (C‐OH), 56.05 (OCH_3_ ), 43.71 (CH_2_ ). **HRMS (ESI) m/z:** Calc. for C_16_H_18_N_2_O_5_S 350.3895, Found 351.1015 (M + H)^+^.

#### 5.3.10 N′‐3‐Hydroxy‐3‐(4‐Methoxyphenyl)Propanoyl Benzenesulfonohydrazide 6j

White solid, Yield 60%, mp: 124.3°C–126.2°C, **IR (ATR) υ**_**max**_ **/cm** ^**−1**^: 3307 (NH), 3167 (OH), 3080 (aromatic CH), 2850 (aliphatic CH), 1681 (C=O), 1550 (aromatic C=C), 1348, 1165 (SO_2_, asymmetric and symmetric vibrations), 1087 (C–O). ^**1**^ **H NMR (400 MHz, DMSO)** δ: 9.95 (s, 1H, NH), 9.81 (s, 1H, NH), 7.75 (d, *J* = 7.5 Hz, 2H, Ar‐H), 7.63–7.61 (m, 1H, Ar‐H), 7.54–7.52 (m, 2H, Ar‐H), 7.17 (d, *J* = 8.1 Hz, 2H, Ar‐H), 6.85 (d, *J* = 8.1 Hz, 2H, Ar‐H), 4.74 (s, 1H), 3.73 (s, 3H, –OCH_3_), 2.39–2.34 (m, 1H), 2.25–2.21 (m, 1H).^**13**^ **C NMR (101 MHz, DMSO)** δ: 169.22 (C=O), 158.72, 139.58, 137.36, 133.30, 129.21, 127.99, 127.40, 113.85, 69.31 (C‐OH), 55.49 (OCH_3_), 43.74 (CH_2_). **HRMS (ESI) m/z:** Calc. for C_16_H_18_N_2_O_5_S 350.0936, Found 350.0919.

#### 5.3.11 4‐Fluoro‐N′‐3‐Hydroxy‐3‐(4‐Methoxyphenyl)Propanoyl Benzenesulfonohydrazide 6k

White solid, Yield 73%, mp: 128.0°C–129.0°C, **IR (ATR) υ**_**max**_ **/cm** ^**−1**^: 3568 (NH), 3348 (OH), 3068 (aromatic CH), 2816 (aliphatic CH), 1658 (C=O), 1589 (aromatic C=C), 1336, 1149 (SO_2_, asymmetric and symmetric vibrations), 1087 (C–O). ^**1**^ **H NMR (400 MHz, DMSO)** δ: 10.06 (s, 1H, NH), 9.89 (s, 1H, NH), 7.83–7.82 (m, 2H), 7.38–7.33 (m, 4H), 6.90 (d, *J* = 37.3 Hz, 2H), 5.67 (br s, 1H, OH), 5.03 (s, 1H), 3.44 (s, 3H, OCH_3_), 2.46–2.36 (m, 2H). ^**13**^ **C NMR (101 MHz, DMSO)** δ: 169.22 (C=O), 163.70, 158.74, 137.42, 135.96, 131.14 (d, ^3^ *J*_*CF*_ = 9.9 Hz), 127.40, 116.33 (d, ^2^ *J*_*CF*_ = 22.7 Hz), 113.85, 69.34 (CH‐OH), 55.50 (OCH_3_), 43.74 (CH_2_). **HRMS (ESI) m/z**: Calc. for C_16_H_17_FN_2_O_5_S 368.38, Found 368.0794.

#### 5.3.12 4‐Methoxy‐N′‐3‐Hydroxy‐3‐(4‐Methoxyphenyl)Propanoyl Benzenesulfonohydrazide 6l

White solid, Yield 55%, mp: 161.2°C–162.5°C, **IR (ATR) υ**_**max**_ **/cm** ^**−1**^: 3288 (NH), 3172 (OH), 3005 (aromatic CH), 2820 (aliphatic CH), 1693 (C=O), 1610 (aromatic C=C), 1301, 1151 (SO_2_, asymmetric and symmetric vibrations), 1087 (C–O). ^**1**^ **H NMR (400 MHz, DMSO)** δ: 10.77 (s, 1H, NH), 10.16 (br s, 1H, NH), 7.51 (d, *J* = 8.7 Hz, 2H), 7.27 (d, *J* = 8.6 Hz, 2H), 6.89 (d, *J* = 8.7 Hz, 2H), 6.85 (d, *J* = 8.8 Hz, 2H), 5.44 (br s, 1H, OH), 4.93 (dd, *J* = 8.7, 4.9 Hz, 1H), 3.74 (s, 3H, OCH_3_), 3.72 (s, 3H, OCH_3_), 2.48–2.44 (m, 2H, CH_2_). ^**13**^ **C NMR (101 MHz, DMSO)** δ: 169.91 (C=O), 159.73, 158.86, 141.18, 137.14, 127.52, 127.40, 113.97, 113.22, 69.28 (CH‐OH), 55.60 (OCH_3_), 55.52 (OCH_3_), 43.78 (CH_2_). **HRMS (ESI) m/z**: Calc. for C_17_H_20_N_2_O_6_S 380.4155, Found 380.1024.

#### 5.3.13 N′‐3‐Hydroxy‐3‐(4‐Fluorophenyl)Propanoylbenzenesulfonohydrazide 6m

Beige solid, Yield 86%, mp: 72.3°C–73.0°C, **IR (ATR) υ**_**max**_ **/cm** ^**−1**^: 3590 (NH), 3331 (OH), 3061 (aromatic CH), 2930, 2810 (aliphatic CH), 1654 (C=O), 1535 (aromatic C=C), 1332, 1157 (SO_2_, asymmetric and symmetric vibrations), 1089 (C–O). ^**1**^ **H NMR (400 MHz, DMSO)** δ: 9.98 (d, *J* = 3.0 Hz, 1H, NH), 9.84 (d, *J* = 2.8 Hz, 1H, NH), 7.73–7.75 (m, 2H, Ar‐H), 7.68–7.50 (m, 3H, Ar‐H), 7.29–7.24 (m, 2H, Ar‐H), 7.13–7.09 (m, 2H, Ar‐H), 4.78 (dd, *J* = 7.6, 5.9 Hz, 1H), 2.37 (dd, *J*_*1*_ = 14.3, *J*_*2*_ = 8.1 Hz, 1H), 2.25 (dd, *J*_*1*_ = 14.3, *J*_*2*_ = 5.5 Hz, 1H). ^**13**^ **C NMR (101 MHz, DMSO)** δ: 169.05 (C=O), 161.66 (d, ^*1*^ *J*_*CF*_ = 242.3 Hz), 144.05, 141.49, 139.57, 133.32, 129.23, 128.16 (d, ^*3*^ *J*_*CF*_ = 8.3 Hz), 127.98, 127.04, 115.16 (d, ^*2*^ *J*_*CF*_ = 21.4 Hz), 69.12 (C‐OH), 43.69 (CH_2_). **HRMS (ESI) m/z:** Calc. for C_15_H_15_FN_2_O_4_S 338.354, Found 338.3443.

#### 5.3.14 4‐Fluoro‐N′‐3‐Hydroxy‐3‐(4‐Fluorophenyl)Propanoyl Benzenesulfonohydrazide 6n

Beige solid, Yield 45%, mp: 132.1°C–134.1°C, **IR (ATR) υ**_**max**_ **/cm** ^**−1**^: 3566 (NH), 3334 (OH), 3068 (aromatic CH), 2810 (aliphatic CH), 1680 (C=O), 1589 (aromatic C=C), 1334, 1149 (SO_2_ , asymmetric and symmetric vibrations), 1087 (C–O). ^**1**^ **H** **NMR (400 MHz, DMSO)** δ: 10.02 (s, 1H, NH), 9.90 (s, 1H, NH), 7.84 (d, *J* = 46.7 Hz, 3H, Ar‐H), 7.32 (d, *J* = 41.4 Hz, 4H, Ar‐H), 7.11 (d, *J* = 16.9 Hz, 2H, Ar‐H), 4.87–4.70 (m, 1H), 2.37 (dd, *J*_*1*_ = 13.0, *J*_*2*_ = 9.6 Hz, 1H), 2.25 (dd, *J*_*1*_ = 14.3, *J*_*2*_ = 3.2 Hz, 1H). ^**13**^ **C** **NMR (101 MHz, DMSO)** δ: 169.06 (C=O), 164.95 (d, ^*1*^ *J*_*CF*_ = 226.3 Hz), 161.69 (d, ^*1*^ *J*_*CF*_ _*’*_ = 242.9 Hz), 144.48, 141.41, 135.79, 131.13 (d, ^*3*^ *J*_*CF*_ = 9.7 Hz), 128.16 (d, ^*3*^ *J*_*CF*_ _*’*_ = 8.1 Hz), 126.87, 116.36 (d, ^*2*^ *J*_*CF*_ = 22.5 Hz), 115.17 (d, ^*2*^ *J*_*CF*_ _*’*_ = 21.3 Hz), 69.12 (C‐OH), 43.63 (CH_2_ ). **HRMS (ESI) m/z:** Calc. for C_15_H_14_F_2_N_2_O_4_S 356.3445, Found 357.0744 (M + H)^+^, 379.0539 (M + Na)^+^.

#### 5.3.15 4‐Methoxy‐N′‐3‐Hydroxy‐3‐(4‐Fluorophenyl)Propanoyl Benzenesulfonohydrazide 6o

Light brown solid, Yield 45%, mp: 88.1°C–89.3°C, **IR (ATR) υ**_**max**_ **/cm** ^**−1**^: 3566 (NH), 3325 (OH), 3074 (aromatic CH), 2923 (aliphatic CH), 1666 (C=O), 1597 (aromatic C=C), 1332, 1151 (SO_2_ , asymmetric and symmetric vibrations), 1091 (C‐O). ^**1**^ **H** **NMR (400 MHz, DMSO)** δ: 9.94 (d, *J* = 2.8 Hz, 1H, NH), 9.64 (d, *J* = 3.1 Hz, 1H, NH), 7.67 (d, *J* = 8.9 Hz, 2H, Ar‐H), 7.28 (dd, *J*_*1*_ = 8.6, *J*_*2*_ = 5.7 Hz, 2H, Ar‐H), 7.13–7.02 (m, 4H, Ar‐H), 4.79 (dd, *J*_*1*_ = 8.0, *J*_*2*_ = 5.6 Hz, 1H), 3.26 (s, 3H), 2.37 (dd, *J*_*1*_ = 14.3, *J*_*2*_ = 8.2 Hz, 1H), 2.25 (dd, *J*_*1*_ = 14.3, *J*_*2*_ = 5.5 Hz, 1H). ^**13**^ **C** **NMR (101 MHz, DMSO)** δ: 168.92 (C=O), 163.01, 145.57, 143.51, 141.57, 131.00, 130.29, 128.17 (d, ^*3*^ *J*_*CF*_ = 8.2 Hz), 127.51, 127.30, 115.16 (d, ^*2*^ *J*_*CF*_ = 21.1 Hz), 114.40, 113.19, 69.12 (C‐OH), 56.05 (OCH_3_ ), 43.69 (CH_2_ ). **HRMS (ESI) m/z:** Calc. for C_16_H_17_FN_2_O_5_S 368.38, Found 368.1526, 369.0920 (M + H)^+^, 391.0754 (M + Na)^+^.

#### 5.3.16 N′‐3‐Hydroxy‐3‐(2‐Thienyl)Propanoyl Benzenesulfonohydrazide 6p

White solid, Yield 63%, mp: 110°C–111°C, **IR (ATR) υ**_**max**_ **/cm** ^**−1**^: 3599 (NH), 3327 (OH), 3070 (aromatic CH), 2933, 2814 (aliphatic CH), 1658 (C=O), 1539 (aromatic C=C), 1322, 1157 (SO_2_, asymmetric and symmetric vibrations), 1087 (C–O). ^**1**^ **H NMR (400 MHz, DMSO)** δ: 10.06 (s, 1H, NH), 9.88 (s, 1H, NH), 7.75 (d, *J* = 8.0 Hz, 2H, Ar‐H), 7.64–7.61 (m, 1H, Ar‐H), 7.54–7.52 (m, 2H, Ar‐H), 7.37 (d, *J* = 5.0 Hz, 1H, Ar‐H), 6.95–6.91 (m, 1H, Ar‐H), 6.82 (d, *J* = 3.3 Hz, 1H, Ar‐H), 5.05–5.00 (m, 1H), 2.46 (d, *J* = 7.9 Hz, 1H), 2.39 (d, *J* = 5.7 Hz, 1H). ^**13**^ **C NMR (101 MHz, DMSO)** δ: 168.79 (C=O), 142.85, 139.42, 133.39, 129.29, 127.98, 127.12, 124.77, 123.45, 65.77 (C–OH), 43.80 (CH_2_). **HRMS (ESI) m/z:** Calc. for C_13_H_14_N_2_O_4_S_2_ 326.3913, Found 349.0293 (M + Na)^+^.

#### 5.3.17 4‐Fluoro‐N′‐3‐Hydroxy‐3‐(2‐Thienyl)Propanoyl Benzenesulfonohydrazide 6q

White solid, Yield 46%, mp: 115.5°C–116.8°C, **IR (ATR) υ**_**max**_ **/cm** ^**−1**^: 3566 (NH), 3350 (OH), 3068 (aromatic CH), 2980 (aliphatic CH), 1668 (C=O), 1587 (aromatic C=C), 1336, 1149 (SO_2_, asymmetric and symmetric vibrations), 1087 (C–O). ^**1**^ **H NMR (400 MHz, DMSO)** δ 9.96 (br s, 1H, NH), 9.81 (br s, 1H, NH), 7.82–7.79 (m, 2H), 7.34 (t, *J* = 8.6 Hz, 1H), 7.18 (d, *J* = 8.1 Hz, 2H), 6.85 (d, *J* = 7.7 Hz, 2H), 4.75–4.72 (m, 1H), 2.41–2.35 (m, 1H), 2.26–2.23 (m, 1H). ^**13**^ **C NMR (101 MHz, DMSO)** δ 168.76 (C=O), 164.98 (d, ^1^ *J*_*CF*_ = 251.0 Hz), 149.69, 135.72, 131.15 (d, ^3^ *J*_*CF*_ = 9.7 Hz), 127.08, 124.81, 123.46, 116.41 (d, ^2^ *J*_*CF*_ = 22.7 Hz), 65.76 (CH‐OH), 43.78 (CH_2_). **HRMS (ESI) m/z:** Calc. for C_16_H_17_FN_2_O_5_S 344.3817, Found 344.0229.

#### 5.3.18 4‐Methoxy‐N′‐3‐Hydroxy‐3‐(2‐Thienyl)Propanoyl Benzenesulfonohydrazide 6r

White solid, Yield 45%, mp: 136.0°C–137.0°C, **IR (ATR) υ**_**max**_ **/cm** ^**−1**^: 3560 (NH), 3320 (OH), 3064 (aromatic CH), 2850 (aliphatic CH), 1691 (C=O), 1591 (aromatic C=C), 1344, 1151 (SO_2_, asymmetric and symmetric vibrations), 1089 (C–O). ^**1**^ **H NMR (400 MHz, DMSO)** δ: 10.02 (s, 1H, NH), 9.68 (s, 1H, NH), 8.91 (d, J = 5.5 Hz, 2H), 8.55 (t, J = 7.8 Hz, 1H), 7.67 (d, J = 7.0 Hz, 2H), 7.03 (d, J = 8.9 Hz, 2H), 5.05–5.01 (m, 1H), 3.82 (s, 3H), 2.46–2.34 (m, 2H). ^**13**^ **C NMR (101 MHz, DMSO)** δ: 168.67 (C=O), 163.02, 149.74, 146.12, 142.86, 130.31, 127.50, 127.10, 124.77, 123.43, 114.44, 65.76 (CH‐OH), 56.05 (OCH_3_), 43.79 (CH_2_). **HRMS (ESI) m/z:** Calc. for C_17_H_20_N_2_O_6_S 356.4172, Found 356.0466.

### 5.4 Biological Evaluation

#### 5.4.1 Anticancer Activity

##### 5.4.1.1 MTS Assay

PANC1 (pancreatic cancer), HCT116 (colon carcinoma), and HDF (human dermal fibroblast) cells were taken out from liquid nitrogen and grown in high‐glucose DMEM (Dulbecco's Modified Eagle Medium) (Gibco 11965084) containing 10% Fetal Bovine Serum (FBS) and 1% PSA (Penicillin–Streptomycin‐Amphotericin B Solution). Cells were seeded in a 96‐well plate at 7500 cells per well and allowed to attach for 24 h. All molecules were dissolved in DMSO (dimethyl sulfoxide) as 100 mM master stocks. The molecules were then delivered to the cells at a final concentration of 20 μM per well and incubated at 37°C for 48 h. After incubation, MTS solution was added to the cells and incubated for an additional 2 h under standard culture conditions. Absorbance was measured at 490 nm using a microplate reader (Multimode Reader Varioskan Lux, ThermoFisher). The absorbance of the blank group was subtracted from the absorbance of the samples, as we have done previously (Biliz et al. 2024; Bülbül et al. 2024).

##### 5.4.1.2 IC_50_ Analysis

Cancer cells were plated in triplicate wells at a density of approximately 70% and incubated for 24 h at 37°C with 5% CO_2_. After the incubation, promising compounds **6a–e**, as well as SKLB1002 and 5‐Fluorouracil as reference compounds, were added to the wells. The compounds were dissolved in DMSO and tested across a range of concentrations from 0.01 to 100 μM. Cell viability was assessed 48 h post‐treatment using the MTS assay. Absorbance was measured at 490 nm, two hours after MTS was added, using a Varioskan LUX Multimode Microplate Reader (Thermo Fisher Scientific). Dose–response curves were generated from the raw absorbance data. The logistic regression model with four or three parameters was applied for analysis. IC_50_ values for the compounds were determined using the IC_50_ Calculation Tool from AATBioquest, as we have done previously (Yıldırım, Ayvaz, et al. 2024; Yıldırım, Kocabaş, et al. 2024).

##### 5.4.1.3 Apoptosis Analysis

‘FITC Annexin V Apoptosis Detection Kit with PI’ (BioLegend, #640914) was used for the apoptosis assay. HTC116 cells in logarithmic growth phase were seeded in six‐well plates at a density of 400,000 cells per well to evaluate the effects of chemicals on cell apoptosis. After 24 h of incubation at 37°C and 5% CO_2_, cells were treated with DMSO or small molecules at a dose of 50 μM. After 72 h of incubation, cells were harvested using trypsin and then centrifuged at 1500 rpm for 5 min. The pellet was resuspended in 400 μL of 1× Annexin V Binding Buffer, and apoptosis was assessed by adding 2 μL of Annexin V‐FITC to the cell suspension. After mixing, cells were incubated for 10 min at RT (25°C) in the dark. Apoptosis analysis was performed by analyzing 10,000 cells per event on the FITC‐A and PC5.5 channels. For final analysis, cells were resuspended in 200 μL of 1× Annexin V Binding Buffer, and 5 μL propidium iodide (PI) was added. Flow cytometry analysis was performed using a Cytoflex S flow cytometer (Beckman), as we have done previously (Meriç et al. 2024).

#### 5.4.2 Antioxidant Activity

The antioxidant capacities and radical scavenging activities of the synthesized *N*‐acyl sulfonohydrazide compounds were determined using CUPRAC (Cu (II) Ion Reducing Antioxidant Capacity) and DPPH (1,1‐Diphenyl‐1‐picrylhydrazyl Radical Quenching Capacity) analysis methods, respectively (Apak et al. 2004; Sztanke and Sztanke 2017). Trolox (TR), ascorbic acid (AA), butylated hydroxyanisole (BHA), and butylated hydroxytoluene (BHT) were employed as reference compounds in antioxidant activity assays.

The synthesized substances were prepared in DMSO at appropriate initial concentrations using the CUPRAC and DPPH methods and then diluted to the desired concentrations with DMSO for use. Copper (II) chloride was prepared at 10 mmol L^−1^ and ammonium acetate (pH = 7.0 buffer) at 1.0 mol L^−1^ in ultrapure water, neocuproine (Nc) at 7.5 mmol L^−1^ in ethyl alcohol, and the DPPH radical solution was prepared at a concentration of 0.1 mmol L^−1^ in methanol.

##### 5.4.2.1 CUPRAC Assay

Add 1.0 mL of 10 mmol L^−1^ Cu (II) solution, 1.0 mL of 7.5 mmol L^−1^ Nc solution, and 1.0 mL of 1.0 mol L^−1^ CH_3_COONH_4_ buffer to a test tube. (×) mL of sample solution and (1.1‐×) mL of solvent were added and vortexed. After preparing the solutions to a total volume of 4.1 mL, the tubes were left to stand at room temperature, closed, for 30 min. After this period, absorbance measurements of the samples were recorded against the reference solution at 450 nm. The method can be summarized as follows:

1.0 mL of 10 mmol L^−1^ Cu (II) solution + 1.0 mL of 7.5 mmol L^−1^ Nc + 1.0 mL of 1 mol L^−1^ CH_3_COONH_4_ + × mL of sample solution + (1.1‐×) mL of solvent. After 30 min, absorbance measurements are taken at 450 nm.

Calibration equations were obtained between the sample concentration and absorbance values, and molar absorption coefficients (ε) were calculated. These values were then compared to the ε_TR_ value obtained using the CUPRAC method for trolox (TR), which was selected as the reference compound, to calculate the trolox equivalent antioxidant capacity (TEAC) coefficients.

##### 5.4.2.2 DPPH Assay

1 mL of freshly prepared DPPH solution in methanol (0.1 mmol L^−1^) was mixed with 0.5 mL of the synthesis samples prepared in the appropriate concentration range and allowed to stand in the dark at room temperature for 30 min. The reference solution was prepared by mixing 0.5 mL of DMSO with 1 mL of DPPH solution. DPPH radical scavenging of the reference solution and each sample was measured after 30 min by taking readings against methanol at λ_max_ 520 nm. The DPPH radical scavenging activity of each synthesized compound is calculated using the following equation:

$$\text{Percent DPPH radical scavenging}=\left[\left(A_{\mathrm{Ref}}-A_{\text{sample}}\right)\times 100\right]/A_{\mathrm{Ref}}$$

Finally, a calibration equation was generated between the percent inhibition rate (y) and the concentration of synthesis products (C).

y=mC+n

where *m* and *n* are the slope and intercept, respectively.

The 50% inhibitory concentration, or IC_50_ (*y* = 50), can be calculated with the following formula:

$${\mathrm{IC}}_{50}=\left(50-n\right)/m$$

Experiments were performed in triplicate, and the results were averaged.

#### 5.4.3 Antimicrobial Activity

##### 5.4.3.1 Determination of Minimum Inhibitory Concentrations (MIC)

The in vitro antibacterial activities against four Gram‐negative bacteria ( *Pseudomonas aeruginosa* ATCC 27853, *Escherichia coli* ATCC 25922, *Klebsiella pneumoniae* ATCC 4352, and *Proteus mirabilis* ATCC 14153) and four Gram‐positive bacteria ( *Staphylococcus aureus* ATCC 29213, *Staphylococcus epidermidis* ATCC 12228, *Enterococcus faecalis* ATCC 29212, and MRSA ATCC 43300) and the in vitro antifungal activities against three Candida species ( *Candida albicans* ATCC 10231, *Candida parapsilosis* ATCC 22019, and *Candida tropicalis* ATCC 750) were determined by the microbe dilution technique using the Clinical Laboratory Standards Institute (CLSI) recommendations (Pierce et al. 2023; Wayne 2002).

For antibacterial assays, bacterial inocula were prepared from 4 to 6 h cultures in Mueller–Hinton broth (Difco, Detroit, USA) and adjusted spectrophotometrically to 5 × 10^5^ cfu/mL. In a U‐bottom 96‐well polystyrene microplate, 50 μL of Mueller–Hinton broth was added to all wells except those in column 1, where 10 mg/mL solutions of the test compounds were applied. Serial two‐fold dilutions were then performed from columns 2–11. Subsequently, 50 μL of the standardized bacterial suspension was added to each well, except well 12 of row A, which served as a sterility control. Plates were incubated at 35°C for 18–24 h, and the minimum inhibitory concentration (MIC) was defined as the lowest concentration of the compound at which no visible bacterial growth occurred. For each bacterial strain, a clinically used standard antibiotic recommended by CLSI was included as a reference.

For antifungal assays, 3–4 colonies from 24 h cultures grown on Sabouraud Dextrose agar (Difco, Detroit, USA) were used to prepare yeast suspensions of 5 × 10^3^ cfu/mL in RPMI‐1640 medium (Sigma). Similar to the antibacterial assay, 50 μL of RPMI‐1640 broth was added to all wells except those in column 1 of the U‐bottom 96‐well microplate, where 10 mg/mL solutions of the test compounds were introduced. Serial dilutions were performed from columns 2–11, followed by the addition of 50 μL of the standardized yeast suspension to all wells except well 12 of row A. The microplates were sealed with sterile lids, placed in nylon sleeves to prevent evaporation, and incubated at 37°C for 48 h. The MIC was defined as the lowest concentration of the compound at which no visible fungal growth was observed. As in the antibacterial assay, reference antifungal agents recommended by CLSI and frequently used in clinical practice were employed as positive controls.

### 5.5 Statistical Analysis

Data are presented as mean ± standard deviation (SD). Unless otherwise stated, all experiments were performed with at least three independent biological replicates (*n* = 3). Statistical significance between the control (DMSO) and treatment groups was evaluated using Student's *t*‐test. A *p*‐value < 0.01 was considered statistically significant.

## Author Contributions

**Tülay Yıldız:** writing – original draft, formal analysis, writing – review and editing. **Belma Hasdemir:** methodology, conceptualization, writing – original draft, writing – review and editing, formal analysis, data curation, project administration, visualization. **Emel Mataracı Kara:** writing – original draft, writing – review and editing, formal analysis. **Hatice Başpınar Küçük:** writing – original draft, writing – review and editing, formal analysis. **Sümbül Yıldırım:** writing – original draft, writing – review and editing, formal analysis. **Hasniye Yaşa:** writing – original draft, writing – review and editing, formal analysis. **Fatih Kocabaş:** writing – original draft, writing – review and editing, visualization, formal analysis. **Ziya Can:** writing – original draft, writing – review and editing, formal analysis.

## Funding

This study was funded by the Health Institutes of Türkiye (TÜSEB). Project number: 33859.

## Conflicts of Interest

The authors declare no conflicts of interest.

## Supporting information


**Figure S1:** Experimental details: general procedures, ^1^H, ^13^C nuclear magnetic resonance spectra, and HRMS spectra of all target products, Tables and Figures in the biological activity and molecular docking studies. ^1^H‐NMR spectrum of **5e** (400 MHz, DMSO).
**Figure S2:** cbdd70344‐sup‐0001‐Supinfo.docx. ^13^C‐NMR spectrum of **5e** (101 MHz, DMSO).
**Figure S3:** cbdd70344‐sup‐0001‐Supinfo.docx. ^1^H‐NMR spectrum of **6a** (400 MHz, DMSO).
**Figure S4:** cbdd70344‐sup‐0001‐Supinfo.docx. ^13^C‐NMR spectrum of **6a** (101 MHz, DMSO).
**Figure S5:** HRMS spectrum of **6a**.
**Figure S6:** cbdd70344‐sup‐0001‐Supinfo.docx. ^1^H‐NMR spectrum of **6b** (400 MHz, DMSO).
**Figure S7:** cbdd70344‐sup‐0001‐Supinfo.docx. ^13^C‐NMR spectrum of **6b** (101 MHz, DMSO).
**Figure S8:** HRMS spectrum of **6b**.
**Figure S9:** cbdd70344‐sup‐0001‐Supinfo.docx. ^1^H‐NMR spectrum of **6c** (400 MHz, DMSO).
**Figure S10:** cbdd70344‐sup‐0001‐Supinfo.docx. ^13^C‐NMR spectrum of **6c** (101 MHz, DMSO).
**Figure S11:** HRMS spectrum of **6c**.
**Figure S12:** cbdd70344‐sup‐0001‐Supinfo.docx. ^1^H‐NMR spectrum of **6d** (400 MHz, DMSO).
**Figure S13:** cbdd70344‐sup‐0001‐Supinfo.docx. ^13^C‐NMR spectrum of **6d** (101 MHz, DMSO).
**Figure S14:** HRMS spectrum of **6d**.
**Figure S15:** cbdd70344‐sup‐0001‐Supinfo.docx. ^1^H‐NMR spectrum of **6e** (400 MHz, DMSO).
**Figure S16:** cbdd70344‐sup‐0001‐Supinfo.docx. ^13^C‐NMR spectrum of **6e** (101 MHz, DMSO).
**Figure S17:** HRMS spectrum of **6e**.
**Figure S18:** cbdd70344‐sup‐0001‐Supinfo.docx. ^1^H‐NMR spectrum of **6f** (400 MHz, DMSO).
**Figure S19:** cbdd70344‐sup‐0001‐Supinfo.docx. ^13^C‐NMR spectrum of **6f** (101 MHz, DMSO).
**Figure S20:** HRMS spectrum of **6f**.
**Figure S21:** cbdd70344‐sup‐0001‐Supinfo.docx. ^1^H‐NMR spectrum of **6g** (400 MHz, DMSO).
**Figure S22:** cbdd70344‐sup‐0001‐Supinfo.docx. ^13^C‐NMR spectrum of **6g** (101 MHz, DMSO).
**Figure S23:** HRMS spectrum of **6g**.
**Figure S24:** cbdd70344‐sup‐0001‐Supinfo.docx. ^1^H‐NMR spectrum of **6h** (400 MHz, DMSO).
**Figure S25:** cbdd70344‐sup‐0001‐Supinfo.docx. ^13^C‐NMR spectrum of **6h** (101 MHz, DMSO).
**Figure S26:** HRMS spectrum of **6h**.
**Figure S27:** cbdd70344‐sup‐0001‐Supinfo.docx. ^1^H‐NMR spectrum of **6i** (400 MHz, DMSO).
**Figure S28:** cbdd70344‐sup‐0001‐Supinfo.docx. ^13^C‐NMR spectrum of **6i** (101 MHz, DMSO).
**Figure S29:** HRMS spectrum of **6i**.
**Figure S30:** cbdd70344‐sup‐0001‐Supinfo.docx. ^1^H‐NMR spectrum of **6j** (400 MHz, DMSO).
**Figure S31:** cbdd70344‐sup‐0001‐Supinfo.docx. ^13^C‐NMR spectrum of **6j** (101 MHz, DMSO).
**Figure S32:** HRMS spectrum of **6j**.
**Figure S33:** cbdd70344‐sup‐0001‐Supinfo.docx. ^1^H‐NMR spectrum of **6k** (400 MHz, DMSO).
**Figure S34:** cbdd70344‐sup‐0001‐Supinfo.docx. ^13^C‐NMR spectrum of **6k** (101 MHz, DMSO).
**Figure S35:** HRMS spectrum of **6k**.
**Figure S36:** cbdd70344‐sup‐0001‐Supinfo.docx. ^1^H‐NMR spectrum of **6l** (400 MHz, DMSO).
**Figure S37:** cbdd70344‐sup‐0001‐Supinfo.docx. ^13^C‐NMR spectrum of **6l** (101 MHz, DMSO).
**Figure S38:** HRMS spectrum of **6l**.
**Figure S39:** cbdd70344‐sup‐0001‐Supinfo.docx. ^1^H‐NMR spectrum of **6m** (400 MHz, DMSO).
**Figure S40:** cbdd70344‐sup‐0001‐Supinfo.docx. ^13^C‐NMR spectrum of **6m** (101 MHz, DMSO).
**Figure S41:** HRMS spectrum of **6m**.
**Figure S42:** cbdd70344‐sup‐0001‐Supinfo.docx. ^1^H‐NMR spectrum of **6n** (400 MHz, DMSO).
**Figure S43:** cbdd70344‐sup‐0001‐Supinfo.docx. ^13^C‐NMR spectrum of **6n** (101 MHz, DMSO).
**Figure S44:** HRMS spectrum of **6n**.
**Figure S45:** cbdd70344‐sup‐0001‐Supinfo.docx. ^1^H‐NMR spectrum of **6o** (400 MHz, DMSO).
**Figure S46:** cbdd70344‐sup‐0001‐Supinfo.docx. ^13^C‐NMR spectrum of **6o** (101 MHz, DMSO).
**Figure S47:** HRMS spectrum of **6o**.
**Figure S48:** cbdd70344‐sup‐0001‐Supinfo.docx. ^1^H‐NMR spectrum of **6p** (400 MHz, DMSO).
**Figure S49:** cbdd70344‐sup‐0001‐Supinfo.docx. ^13^C‐NMR spectrum of **6p** (101 MHz, DMSO).
**Figure S50:** HRMS spectrum of **6p**.
**Figure S51:** cbdd70344‐sup‐0001‐Supinfo.docx. ^1^H‐NMR spectrum of **6q** (400 MHz, DMSO).
**Figure S52:** cbdd70344‐sup‐0001‐Supinfo.docx. ^13^C‐NMR spectrum of **6q** (101 MHz, DMSO).
**Figure S53:** HRMS spectrum of **6q**.
**Figure S54:** cbdd70344‐sup‐0001‐Supinfo.docx. ^1^H‐NMR spectrum of **6r** (400 MHz, DMSO).
**Figure S55:** cbdd70344‐sup‐0001‐Supinfo.docx. ^13^C‐NMR spectrum of **6r** (101 MHz, DMSO).
**Figure S56:** HRMS spectrum of **6r**.
**Table S1:** Assessment of cell viability after treatment in pancreatic cancer (PANC‐1 Cell).
**Table S2:** Antibacterial activity results of *N*‐acyl sulfonohydrazides **6a‐r** (MIC (μg/mL)).
**Table S3:** Antifungal activity results of hydrazide‐sulfonamide compounds **6a‐r**.
**Table S4:** SMILES codes of molecules used in the docking study and for comparisons.
**Table S5:** Molecular docking of compounds to VEGFR and other cancer drug targets.
**Figure S57:** Comparative binding affinity analysis of compounds **6a‐r** against multiple cancer‐related protein targets.
**Figure S58:** Docking poses of reference ligands and compounds **6a‐e** in the VEGFR2 active.
**Figure S59:** Two‐dimensional protein‐ligand interaction diagrams of docked compounds.
**Table S6:** Drug‐likeness analysis of studied compounds **6a‐r**.

## Data Availability

The data that support the findings of this study are available on request from the corresponding author. The data are not publicly available due to privacy or ethical restrictions.
