# Supplementary material for: Design, Synthesis, and Biological Evaluation of Novel N‐Acyl Sulfonohydrazides Derived From β‐Hydroxy Esters: Anticancer, Antioxidant, and Antimicrobial Activities
Source: Chem Biol Drug Des. 2026 Jun 19;107(6):e70344. doi: 10.1111/cbdd.70344 (PMC13282545; doi:10.1111/cbdd.70344)
Supplement: Supplementary file 1 — Figure S1: Experimental details: general procedures, 1H, 13C nuclear magnetic resonance spectra, and HRMS spectra of all target products, Tables and Figures in the biological activity and molecular docking studies. 1H‐NMR spectrum of 5e (400 MHz, DMSO). Figure S2: cbdd70344‐sup‐0001‐Supinfo.docx. 13C‐NMR spectrum of 5e (101 MHz, DMSO). Figure S3: cbdd70344‐sup‐0001‐Supinfo.docx. 1H‐NMR spectrum of 6a (400 MHz, DMSO). Figure S4: cbdd70344‐sup‐0001‐Supinfo.docx. 13C‐NMR spectrum of 6a (101 MHz, DMSO). Figure S5: HRMS spectrum of 6a. Figure S6: cbdd70344‐sup‐0001‐Supinfo.docx. 1H‐NMR spectrum of 6b (400 MHz, DMSO). Figure S7: cbdd70344‐sup‐0001‐Supinfo.docx. 13C‐NMR spectrum of 6b (101 MHz, DMSO). Figure S8: HRMS spectrum of 6b. Figure S9: cbdd70344‐sup‐0001‐Supinfo.docx. 1H‐NMR spectrum of 6c (400 MHz, DMSO). Figure S10: cbdd70344‐sup‐0001‐Supinfo.docx. 13C‐NMR spectrum of 6c (101 MHz, DMSO). Figure S11: HRMS spectrum of 6c. Figure S12: cbdd70344‐sup‐0001‐Supinfo.docx. 1H‐NMR spectrum of 6d (400 MHz, DMSO). Figure S13: cbdd70344‐sup‐0001‐Supinfo.docx. 13C‐NMR spectrum of 6d (101 MHz, DMSO). Figure S14: HRMS spectrum of 6d. Figure S15: cbdd70344‐sup‐0001‐Supinfo.docx. 1H‐NMR spectrum of 6e (400 MHz, DMSO). Figure S16: cbdd70344‐sup‐0001‐Supinfo.docx. 13C‐NMR spectrum of 6e (101 MHz, DMSO). Figure S17: HRMS spectrum of 6e. Figure S18: cbdd70344‐sup‐0001‐Supinfo.docx. 1H‐NMR spectrum of 6f (400 MHz, DMSO). Figure S19: cbdd70344‐sup‐0001‐Supinfo.docx. 13C‐NMR spectrum of 6f (101 MHz, DMSO). Figure S20: HRMS spectrum of 6f. Figure S21: cbdd70344‐sup‐0001‐Supinfo.docx. 1H‐NMR spectrum of 6g (400 MHz, DMSO). Figure S22: cbdd70344‐sup‐0001‐Supinfo.docx. 13C‐NMR spectrum of 6g (101 MHz, DMSO). Figure S23: HRMS spectrum of 6g. Figure S24: cbdd70344‐sup‐0001‐Supinfo.docx. 1H‐NMR spectrum of 6h (400 MHz, DMSO). Figure S25: cbdd70344‐sup‐0001‐Supinfo.docx. 13C‐NMR spectrum of 6h (101 MHz, DMSO). Figure S26: HRMS spectrum of 6h. Figure S27: cbdd70344‐sup‐0001‐Supinfo.docx. 1H‐NMR s [file CBDD-107-e70344-s001.docx]

**Supporting Information**

**Design, Synthesis, and Biological Evaluation of Novel N-Acyl Sulfonohydrazides Derived from β-Hydroxy Esters: Anticancer, Antioxidant, and Antimicrobial Activities**

Belma Hasdemir^1^*, Tülay Yıldız^1^, Hasniye Yaşa^1^, Hatice Başpınar Küçük^1^, Emel Mataracı Kara^2^, Sümbül Yıldırım^3^, Fatih Kocabaş^4^, Ziya Can^5^

*^1^Department of Chemistry, Organic Chemistry Division, Faculty of Engineering, Istanbul University-Cerrahpaşa, Avcılar, Istanbul, Turkey*

*^2^Department of Pharmaceutical Microbiology, Faculty of Pharmacy, Istanbul University, Beyazıt, Istanbul, Turkey*

*^3^Department of Genetics and Bioengineering, Faculty of Engineering, Yeditepe University, Istanbul, Turkey*

*^4^Department of Molecular Biology and Genetics, Faculty of Engineering and Natural Sciences, Istanbul Atlas University, Istanbul, Turkey*

*^5^Department of Chemistry, Analytical Chemistry Division, Faculty of Engineering, Istanbul University-Cerrahpaşa, Avcılar, Istanbul, Turkey*

*Corresponding authors Email: [b.hasdemir@iuc.edu.tr](mailto:b.hasdemir@iuc.edu.tr)

**Table of contents**

| \|  \| **Page** \| \| --- \| --- \| \| Materials and Apparatus………………………………………………. \| S2 \| \| General procedure for the synthesis of hydrazide compounds (**5a-f**)….. \| S2 \| \| Procedure for the synthesis of sulfonohydrazides (**6a-r**) …………….. \| S3 \| \| Copies of the ^1^H NMR, ^13^C NMR, and HRMS spectra of the synthesized compounds……………………………………………….. \| S4 \| \| Biological activity studies……………………………………………... \| S32 \| \| Molecular docking studies……………………………………………. \| S34 \| \| Analysis of compounds for their potent anticancer drug-like properties……………………………………………………………. \| S36 \| |  |
| --- | --- | --- | --- | --- | --- | --- | --- | --- | --- | --- | --- | --- | --- | --- | --- | --- | --- |

**1. Materials and Apparatus**

All reagents and solvents used in the synthesis process and biological activity studies were obtained from commercial suppliers and used without further purification. Analytical TLC of all reactions was performed on Merck prepared plates (silica gel 60 F-254 on aluminum). Melting points were determined with a Buchi B-540 melting point apparatus and were uncorrected. NMR spectra were obtained on a Bruker 400 spectrometer (^1^H at 400 MHz and ^13^C at 101 MHz) in DMSO-*d_6_* (3.4 ppm and 2.50 ppm for ^1^H nuclei and 39.6 ppm for ^13^C nuclei) using TMS as an internal standard. HRMS analysis was performed at the METU (Middle East Technical University) Central laboratory using the ESI technique. The infrared (IR) spectra were recorded on a Shimadzu IRTracer-100 FTIR spectrometer, using the single reflection diamond ATR module. In the antioxidant activity study, the Ohaus PA214C brand balance was used for weighing the chemical substances, the Daihan WiseClean brand ultrasonic bath was used for solubilization processes, and the Shimadzu UV-1800 spectrophotometer with 10 mm beam path cuvettes was used for spectrophotometric measurements. 1,1-diphenyl-2-picrylhydrazyl radical (DPPH) (Sigma-Aldrich) was used for the measurement of radical scavenging activity by the DPPH method. Trolox (TR) was studied for the calculation of trolox equivalent antioxidant capacities (TEAC). Additionally, butylated hydroxyanisole (BHA), butylated hydroxytoluene (BHT), ascorbic acid (AA), and TR were studied as standard antioxidants in the DPPH method. DMSO (Supelco), ethanol (Isolab), methanol (Merck), and ultrapure water were used as solvents.

Methyl 2-oxocyclopentanecarboxylate **3a** and methyl 2-oxocyclohexanecarboxylate **3b** were synthesized according to the reported procedures (Fraga et al. 2004). The reduction of the corresponding β-keto esters with NaBH_4_ prepared the β-hydroxy esters **4a-f** (Hasdemir 2015; Hasdemir et al. 2012; Hasdemir and Yusufoğlu 2004).

**2. Chemistry**

**2.1. General procedure for the synthesis of hydrazide compounds** **(5a-f)**

Hydrazine hydrate (80%) (8.32 mmol) was slowly added to the solution of β-hydroxy ester **4a-f (**5.2 mmol) in ethanol (5 mL), and the mixture was refluxed at 70 °C for 6 hours. The solvent was removed under vacuum. The crude product was recrystallized from ethanol. Hydrazide compounds **5a-f** were obtained in a 50-70% isolated yield.

**Scheme S1.** Synthesis of compounds **5a-f**

**2.2. General procedure for the synthesis of sulfonohydrazides** **(6a-r)**

Hydrazide compounds **5a-f** (1 mmol) and substituted benzenesulfonyl chlorides (1 mmol) were stirred in pyridine (10 mL) at room temperature for 24 hours. The solvent was removed under vacuum. The crude products were purified by recrystallization from ethanol, affording the corresponding sulfonohydrazide derivatives **6a-r** in isolated yields ranging from 45% to 86%.

**Scheme S2.** Synthesis of sulfonohydrazides **6a-r**

**Copies of the ^1^H NMR, ^13^C NMR, and HRMS spectra of the synthesized compounds**


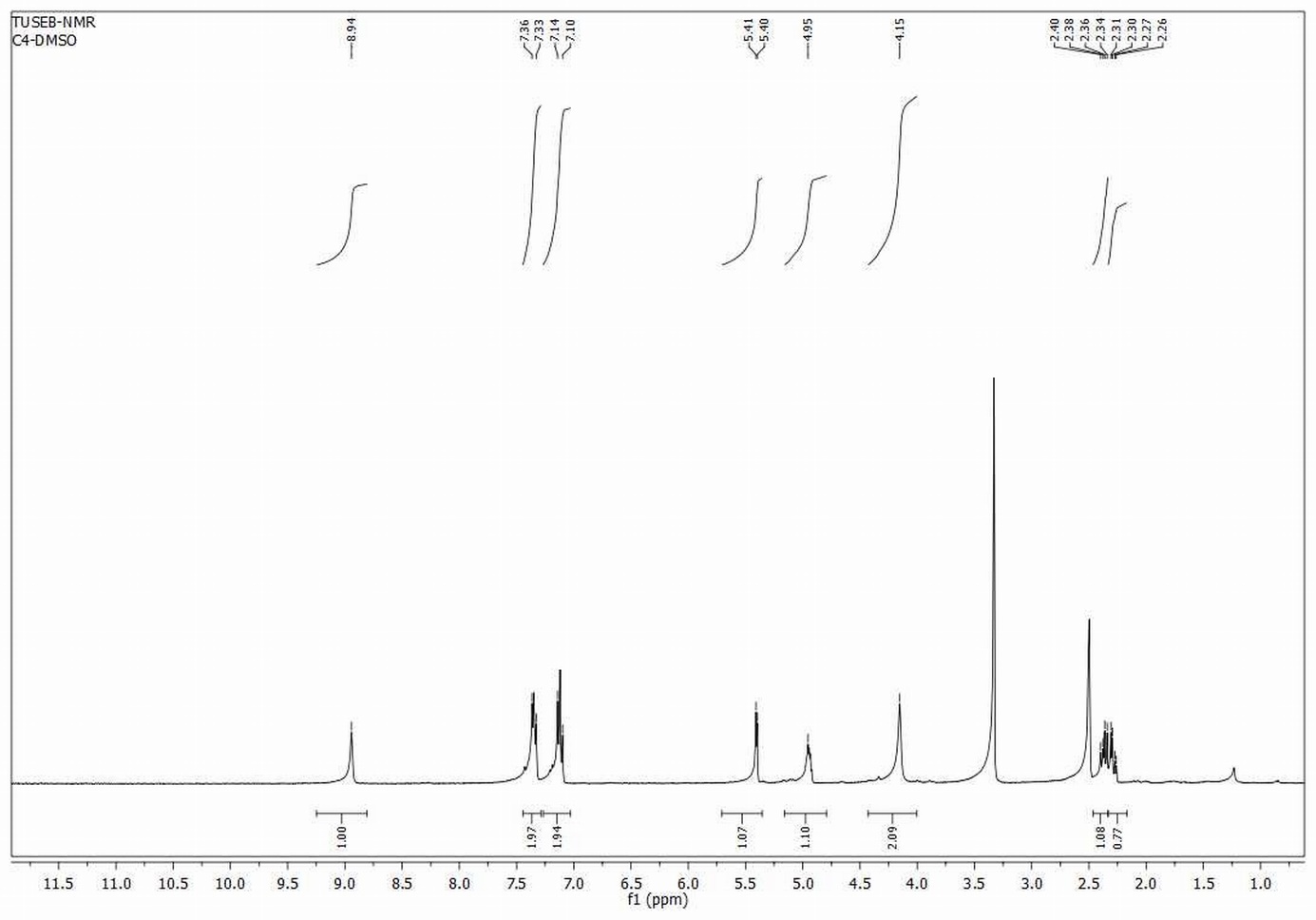


**Figure S1**. ^1^H-NMR spectrum of **5e** (400 MHz, DMSO)


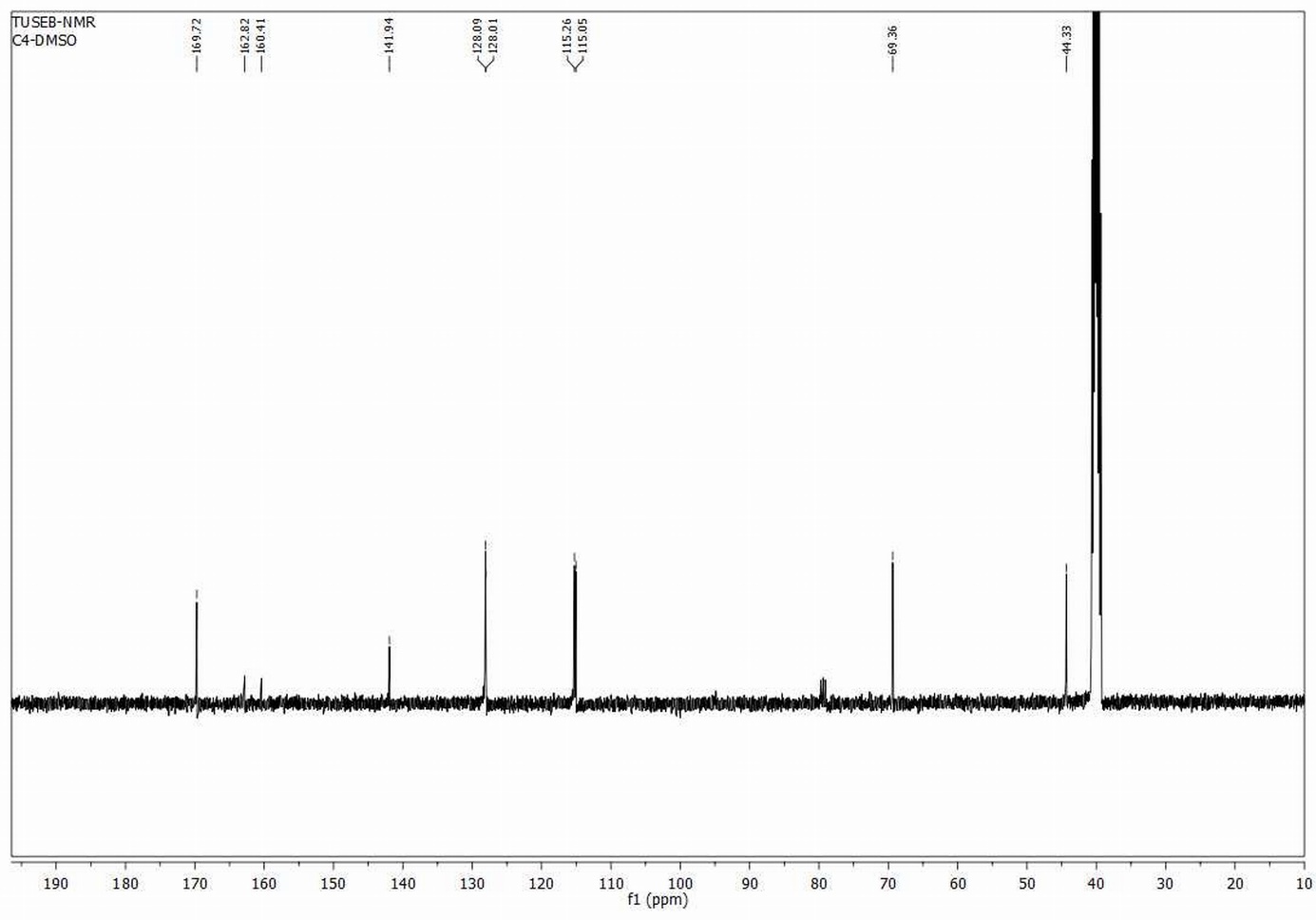


**Figure S2**. ^13^C-NMR spectrum of **5e** (101 MHz, DMSO)


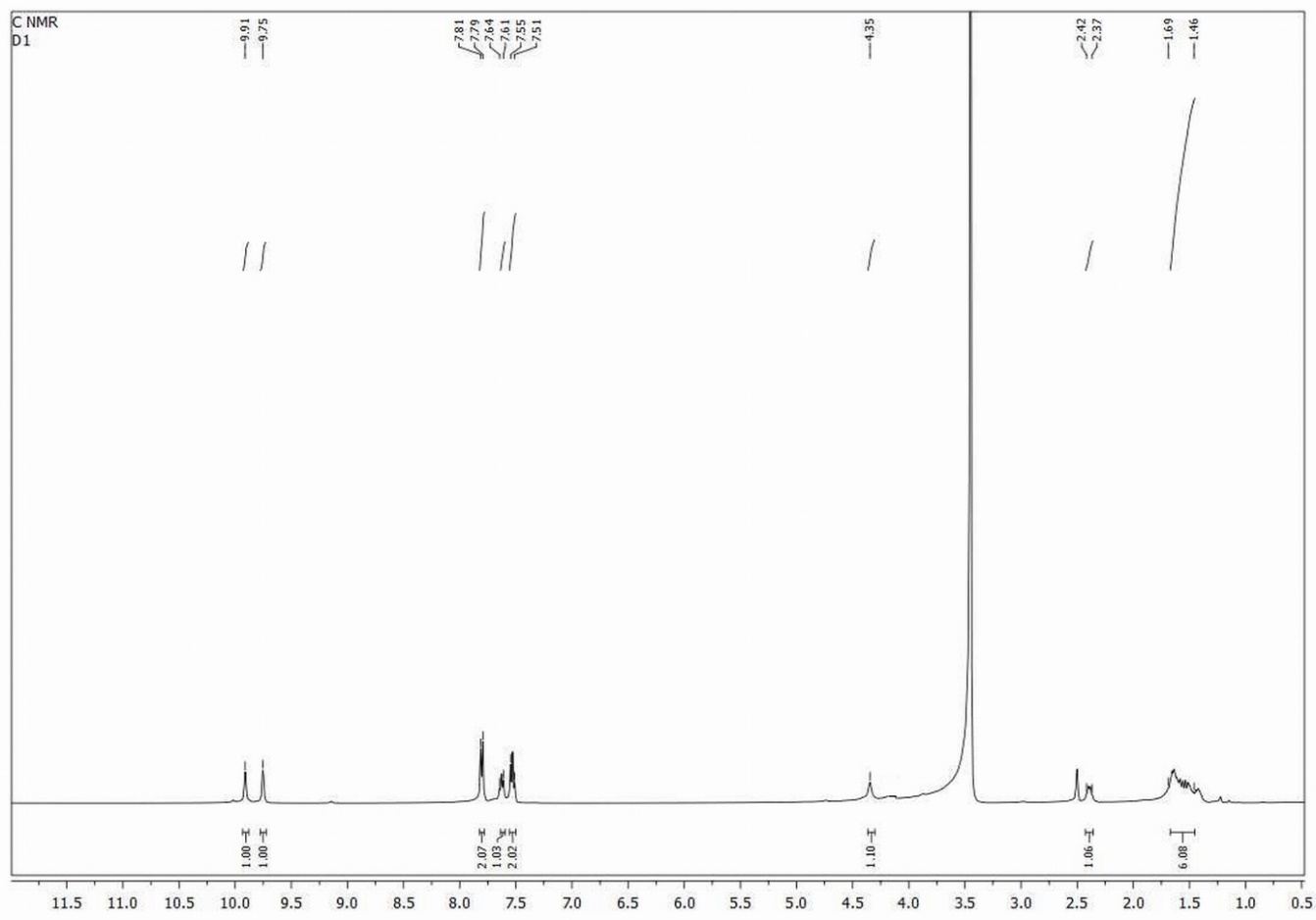


**Figure S3**. ^1^H-NMR spectrum of **6a** (400 MHz, DMSO)


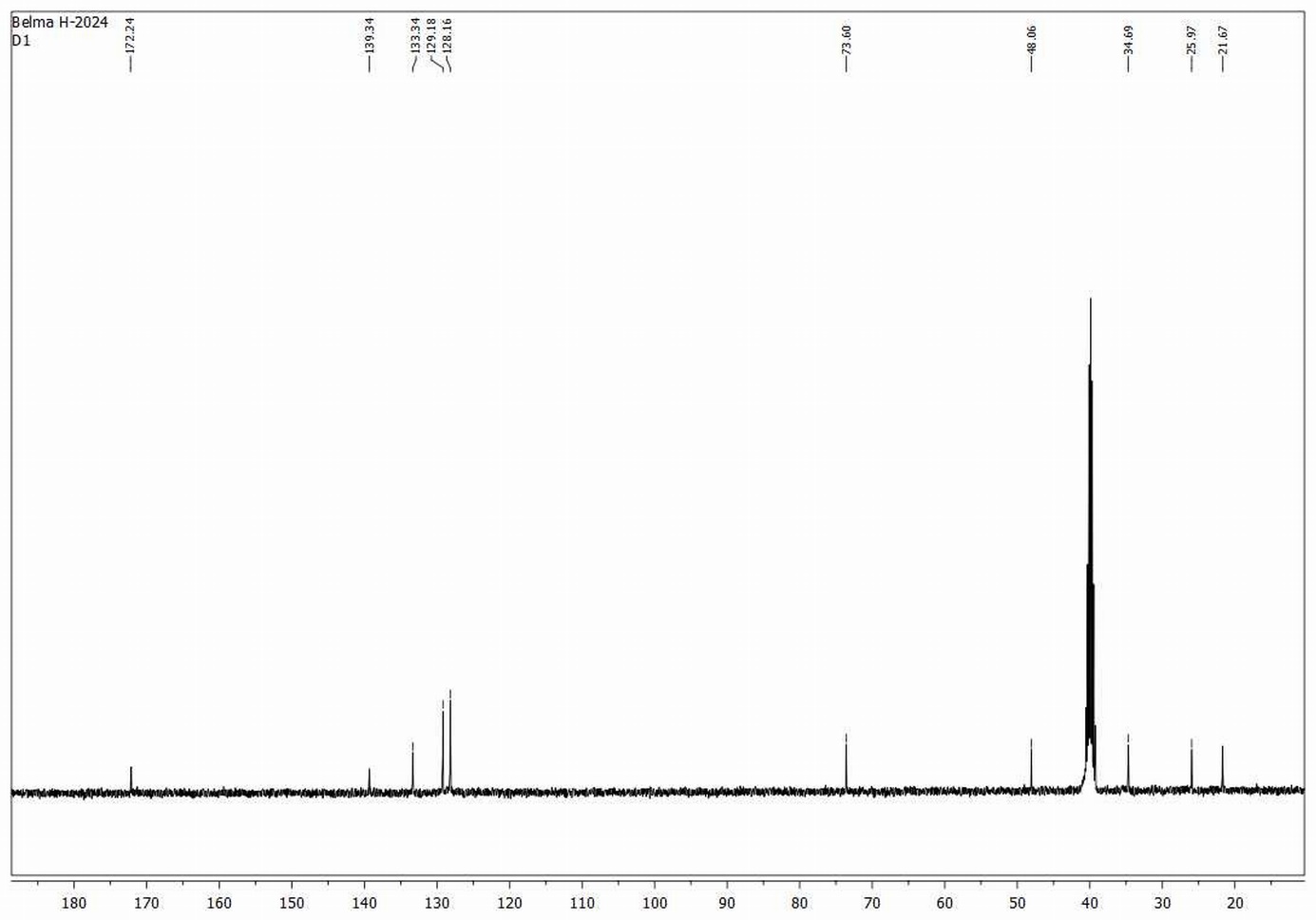


**Figure S4**. ^13^C-NMR spectrum of **6a** (101 MHz, DMSO)

**
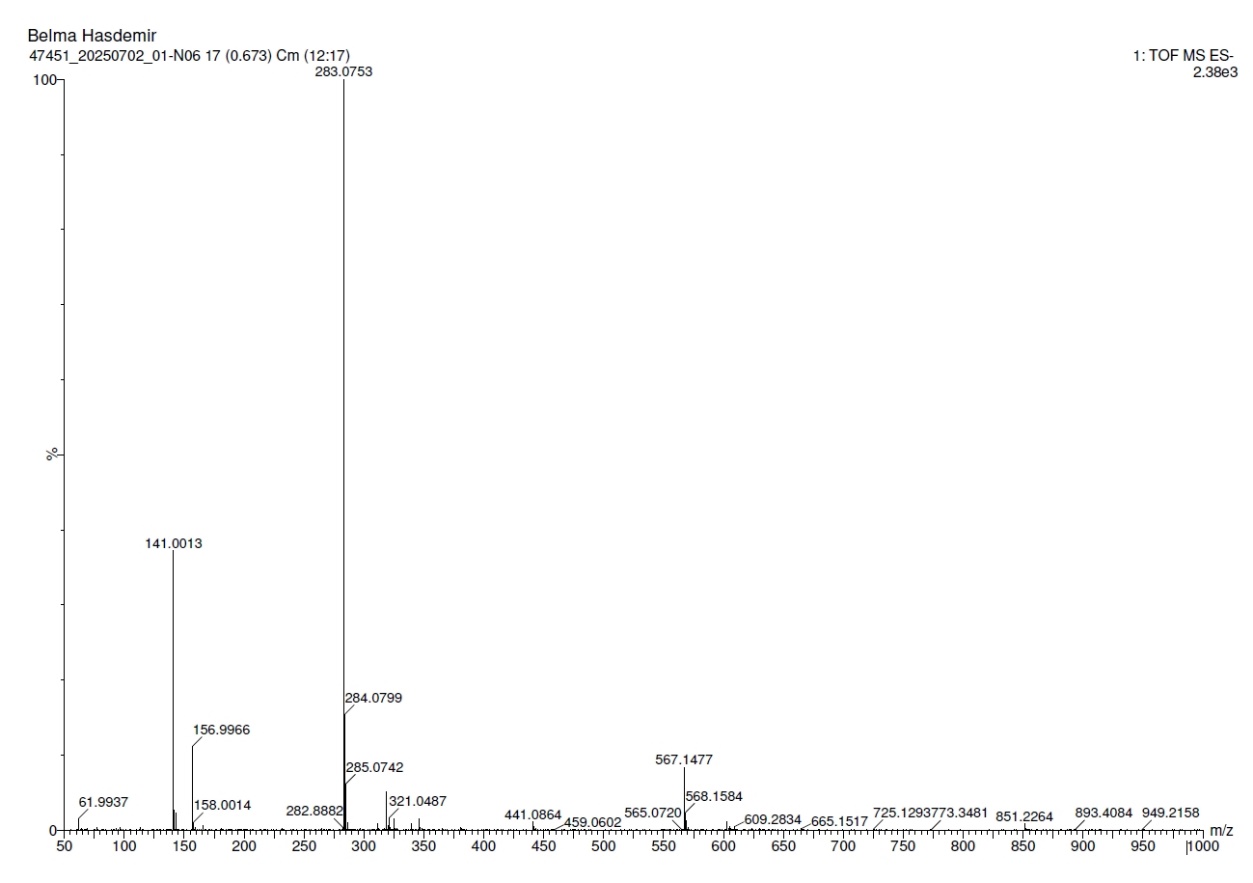
**

**Figure S5**. HRMS spectrum of **6a**


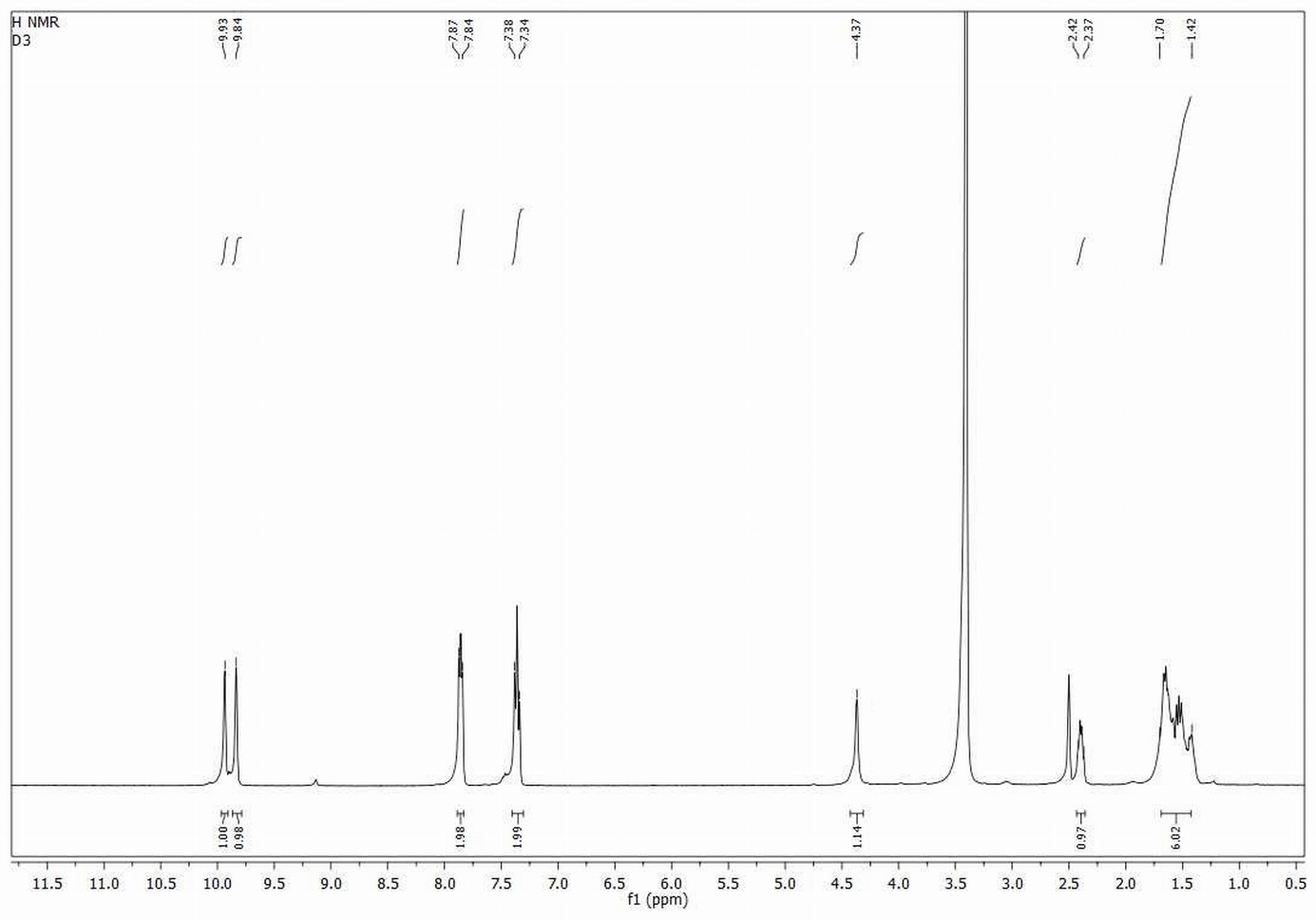


**Figure S6**. ^1^H-NMR spectrum of **6b** (400 MHz, DMSO)


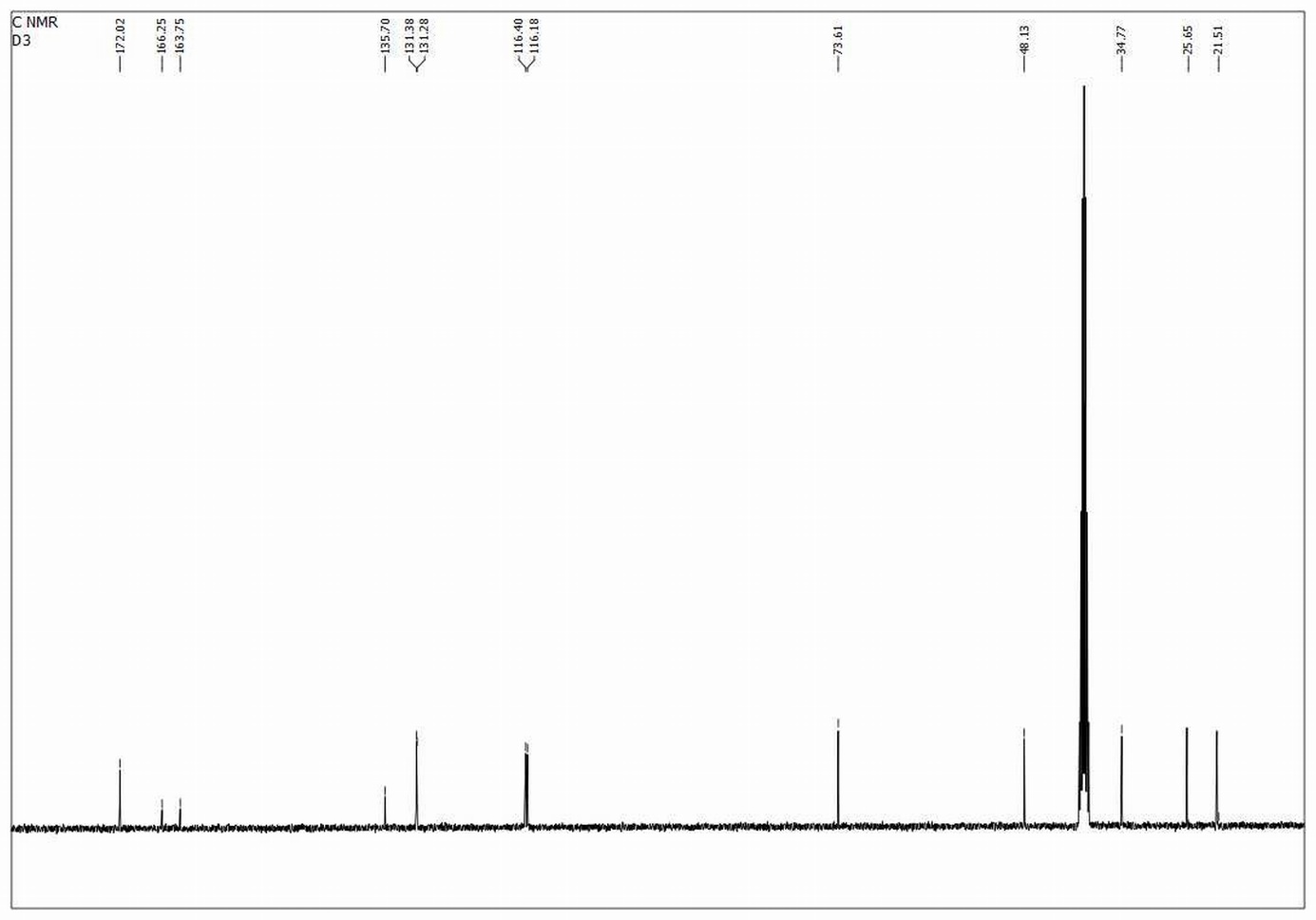


**Figure S7**. ^13^C-NMR spectrum of **6b** (101 MHz, DMSO)


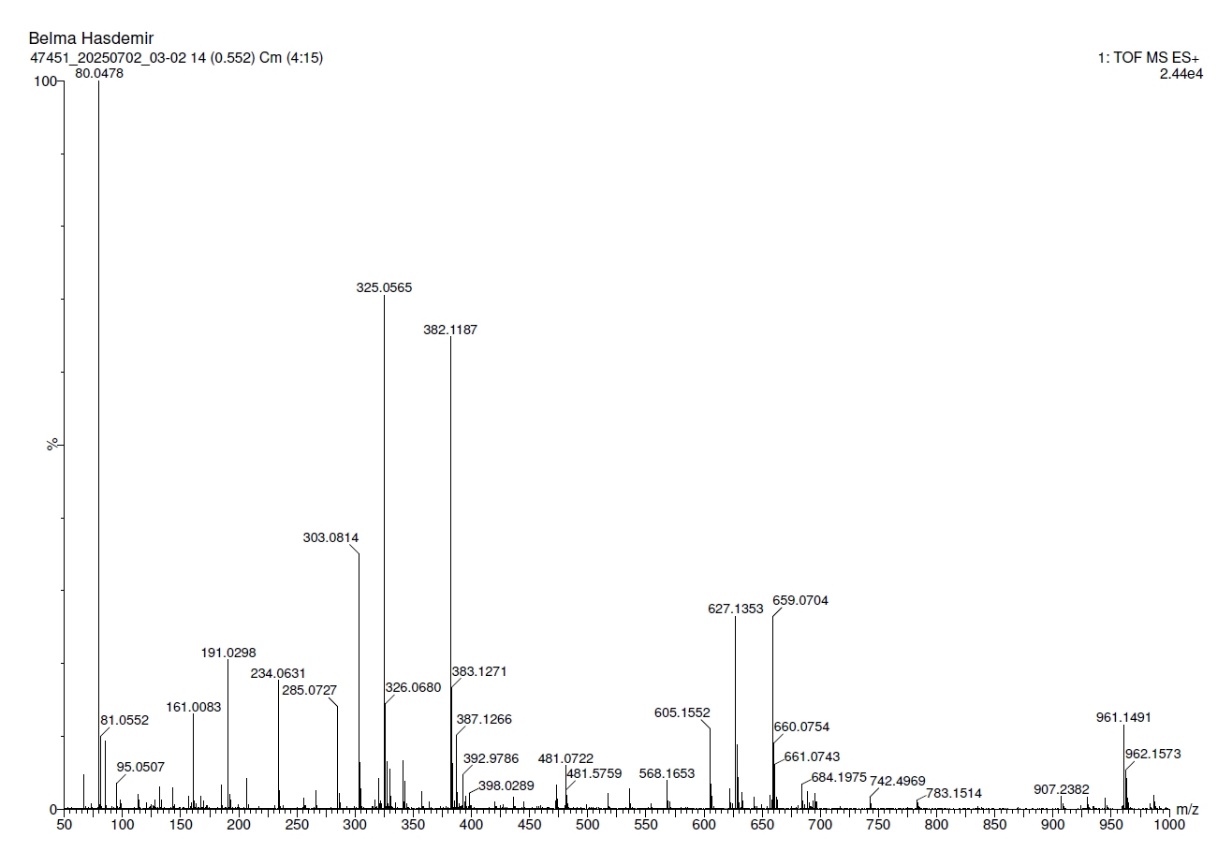


**Figure S8**. HRMS spectrum of **6b**

**
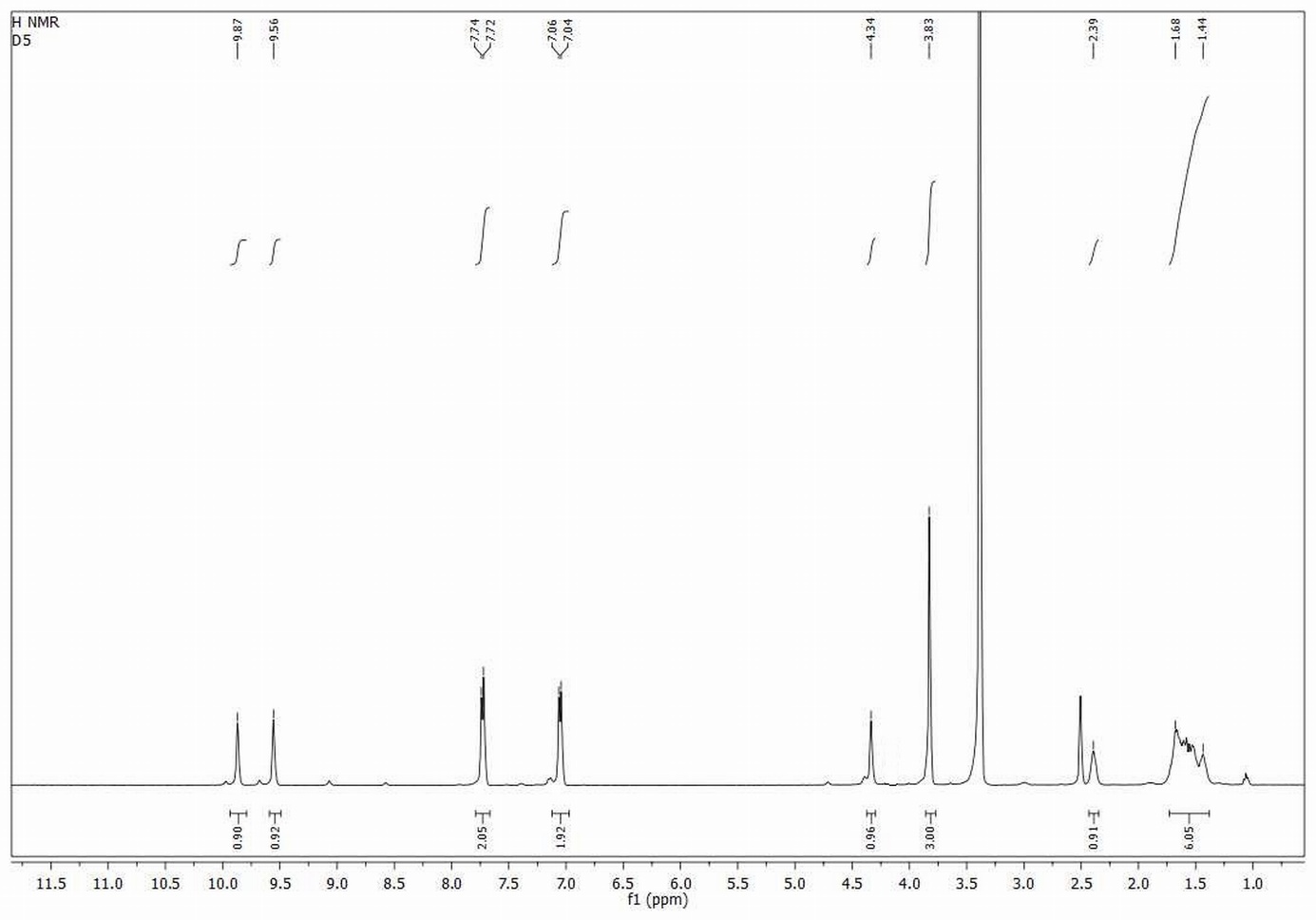
**

**Figure S9**. ^1^H-NMR spectrum of **6c** (400 MHz, DMSO)

**
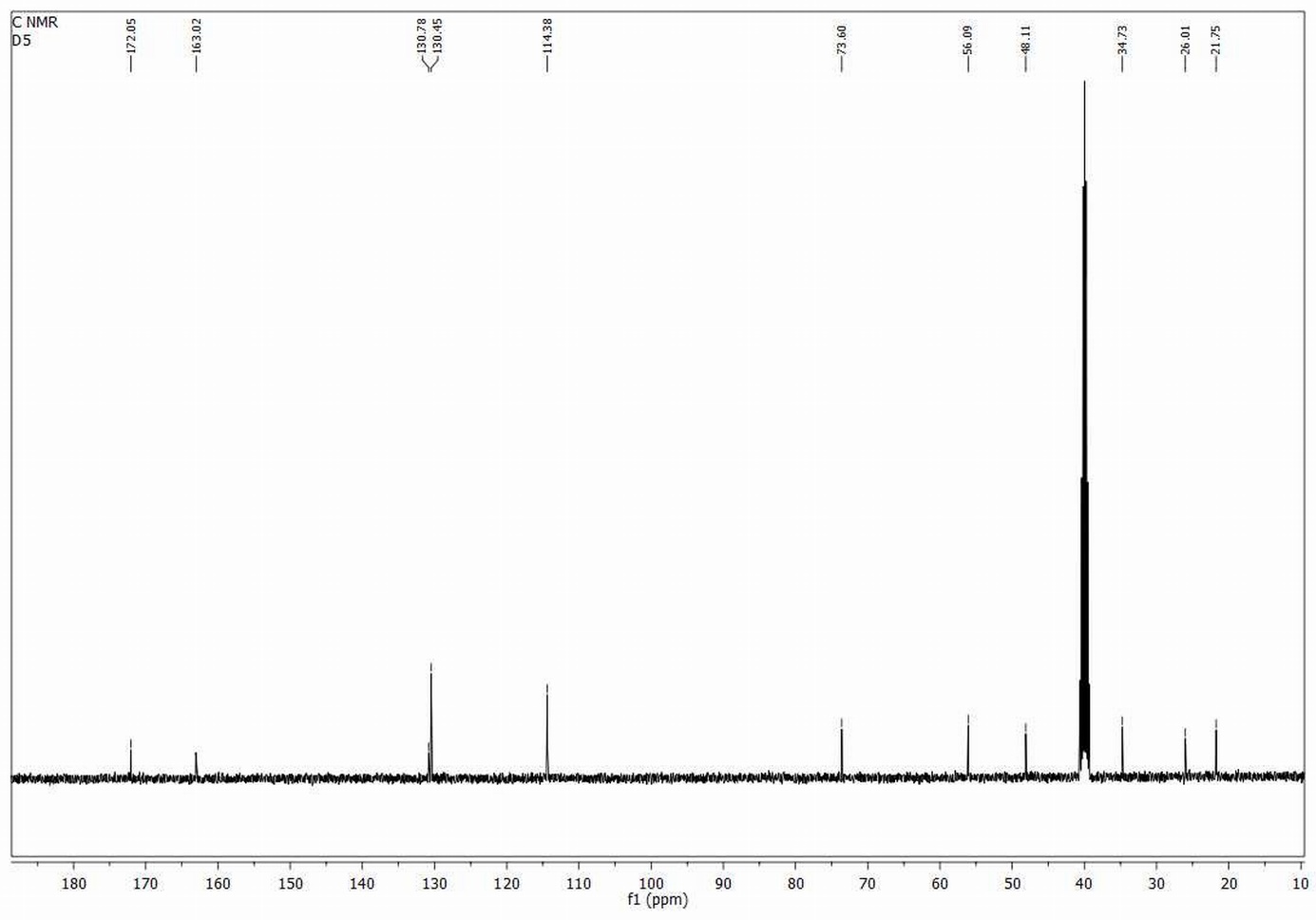
**

**Figure S10**. ^13^C-NMR spectrum of **6c** (101 MHz, DMSO)


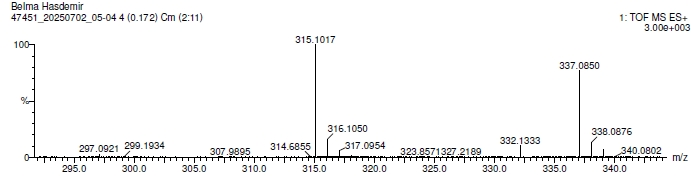


**Figure S11**. HRMS spectrum of **6c**


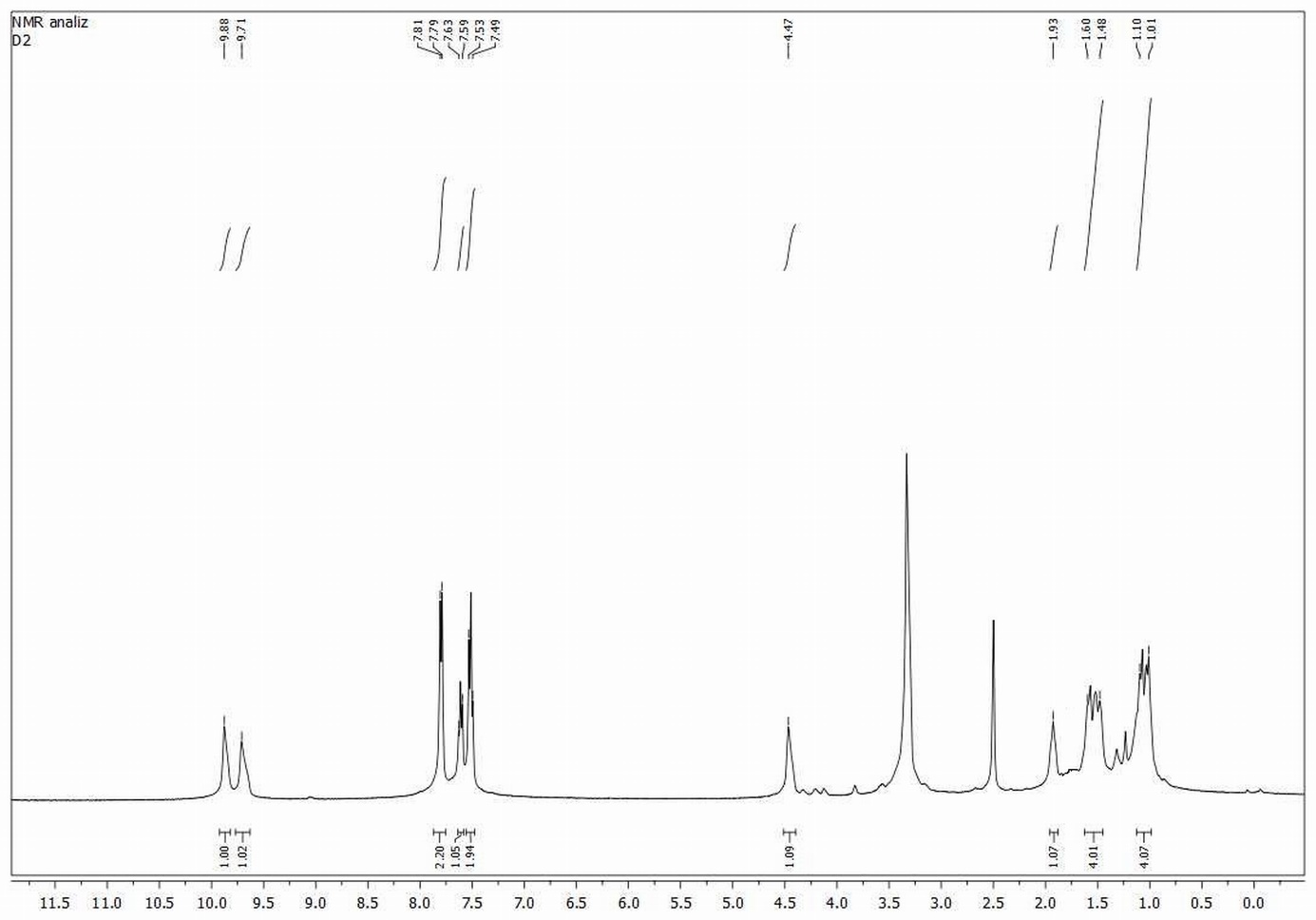


**Figure S12**. ^1^H-NMR spectrum of **6d** (400 MHz, DMSO)


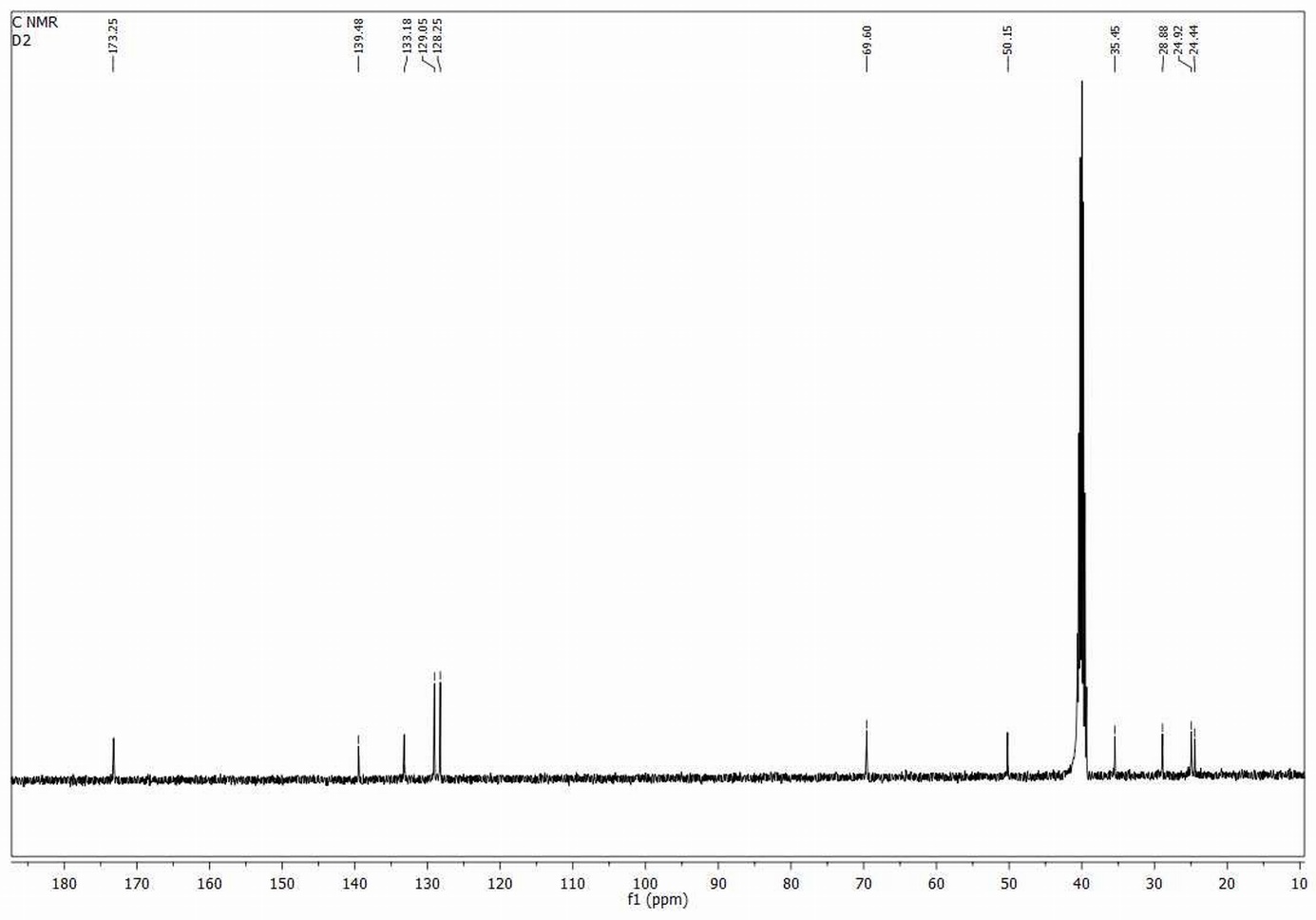


**Figure S13**. ^13^C-NMR spectrum of **6d** (101 MHz, DMSO)


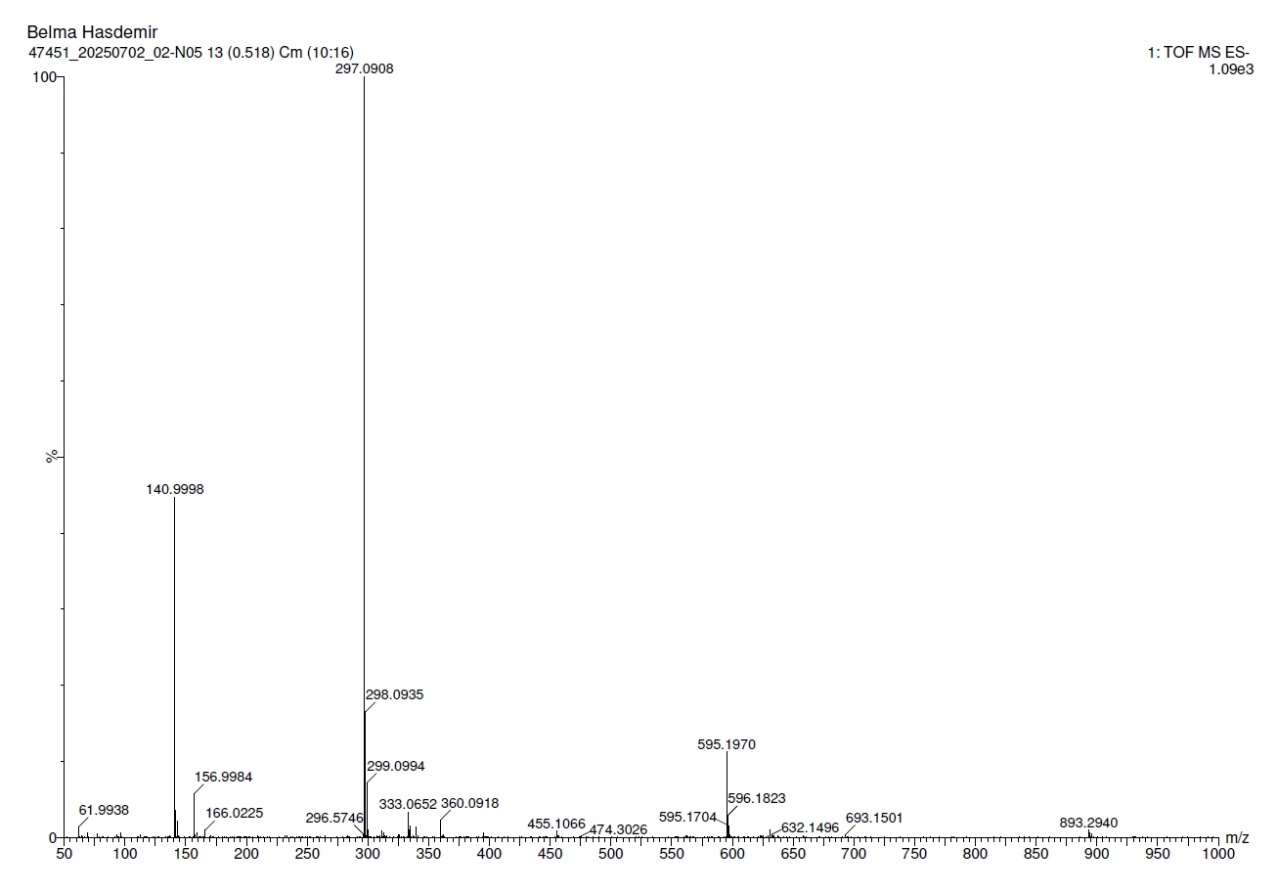


**Figure S14**. HRMS spectrum of **6d**


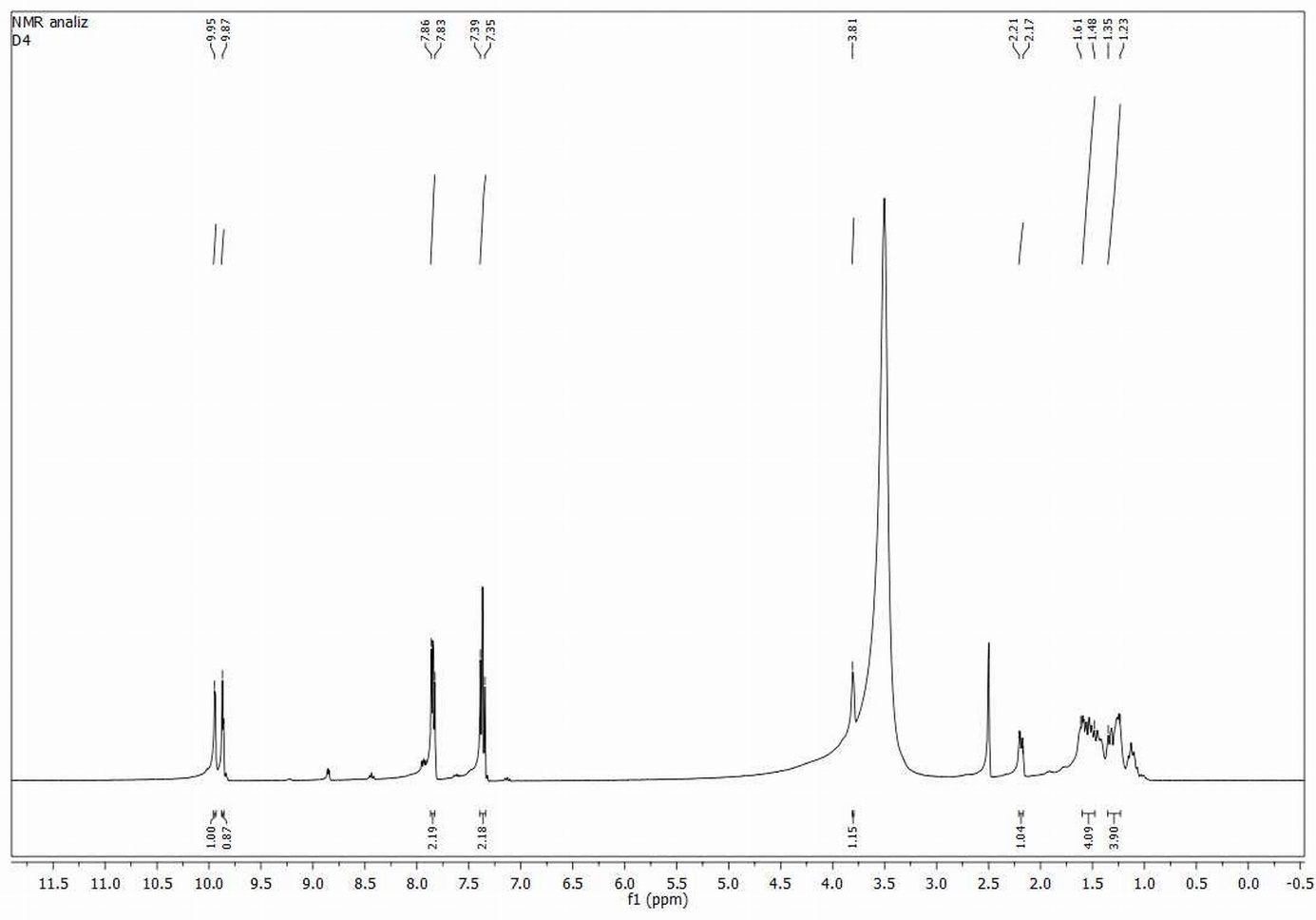


**Figure S15**. ^1^H-NMR spectrum of **6e** (400 MHz, DMSO)


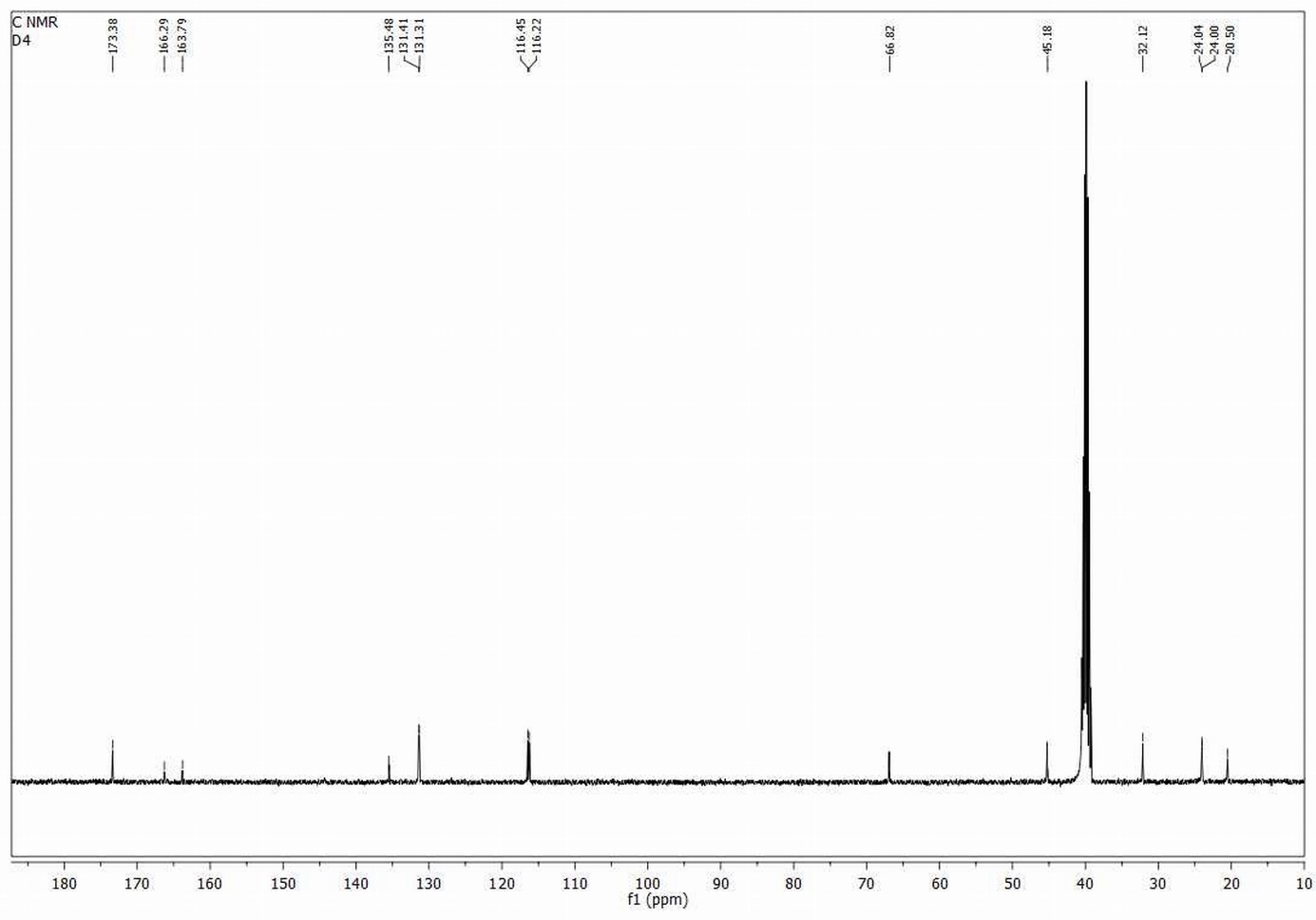


**Figure S16**. ^13^C-NMR spectrum of **6e** (101 MHz, DMSO)


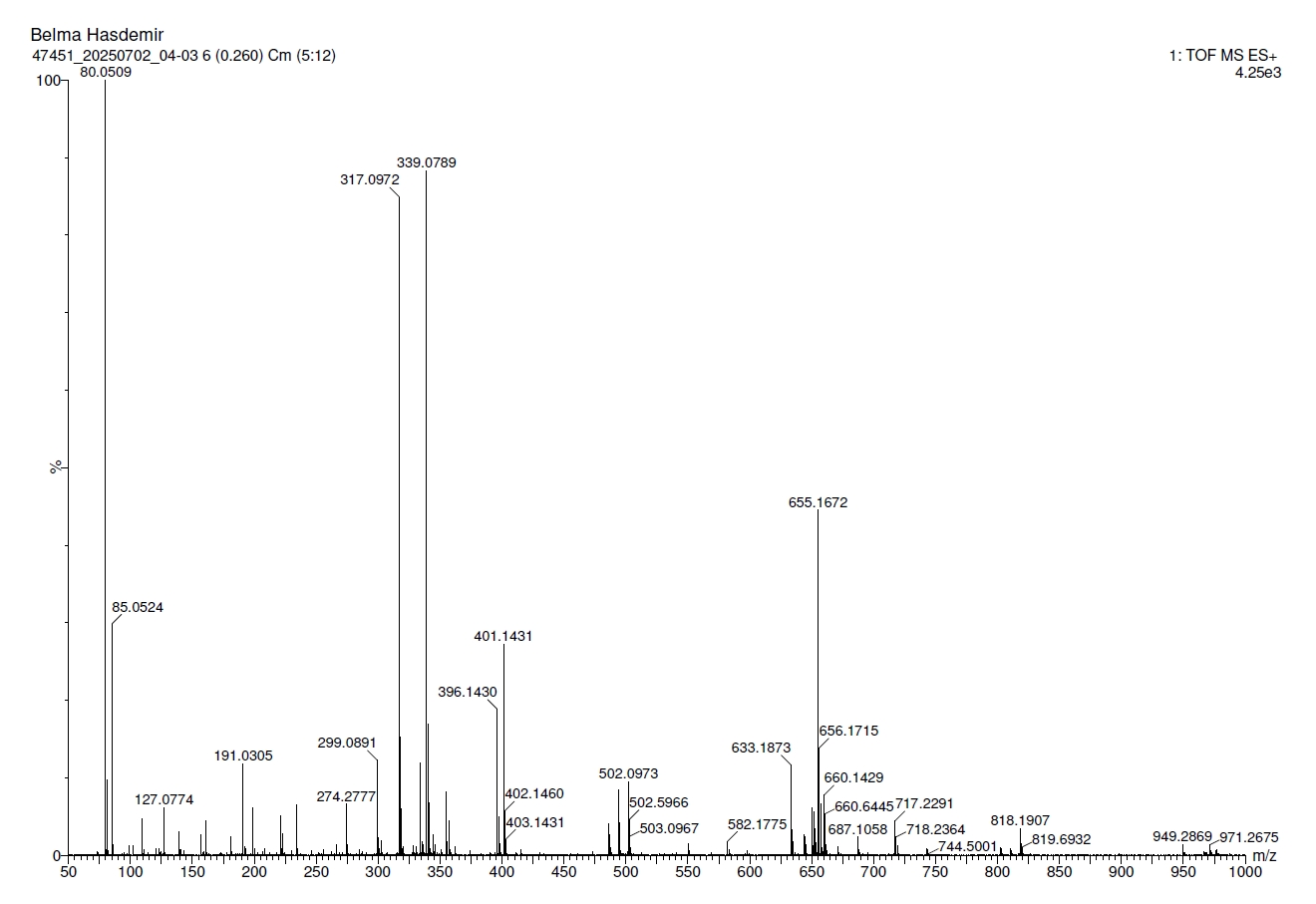


**Figure S17**. HRMS spectrum of **6e**


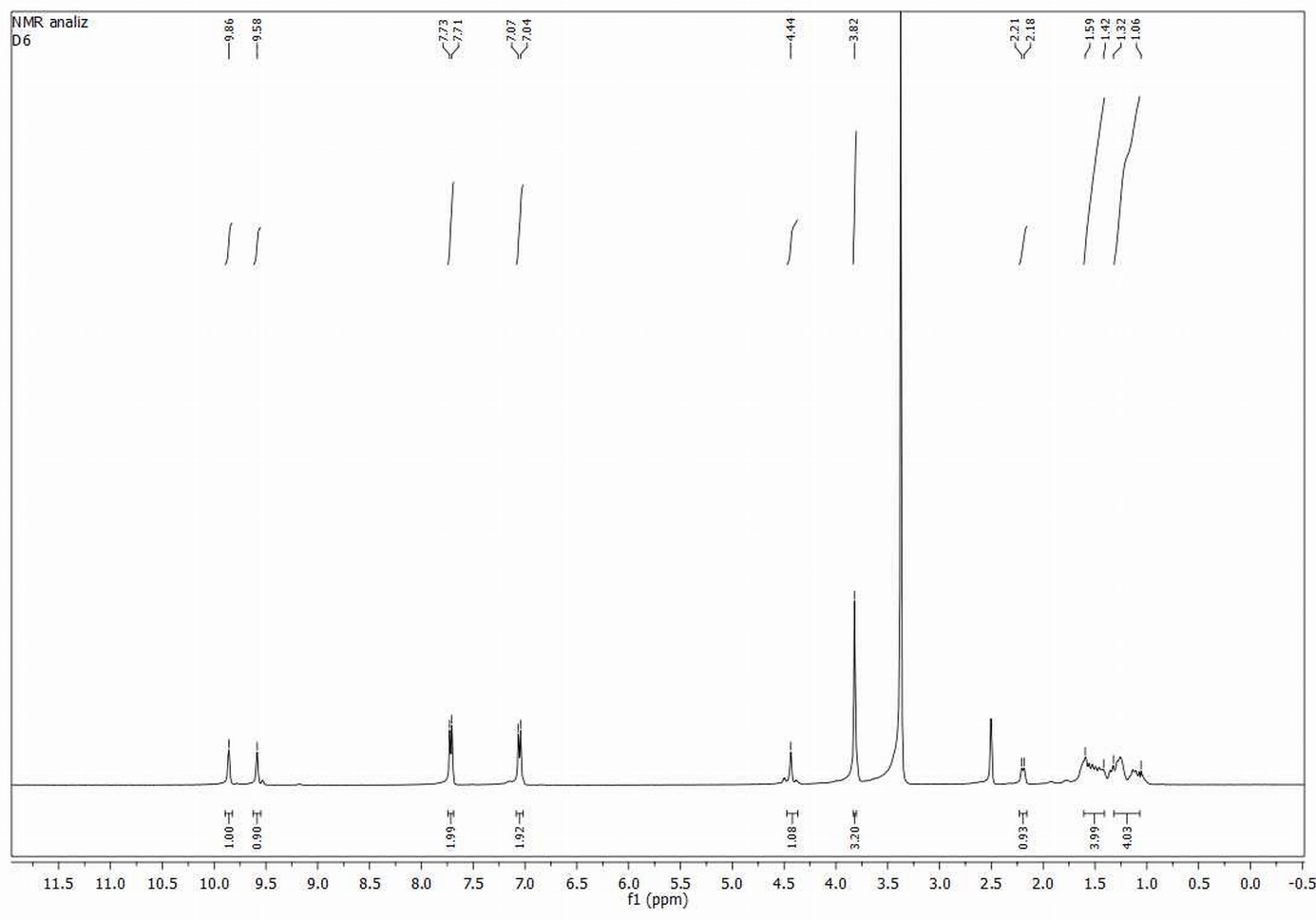


**Figure S18**. ^1^H-NMR spectrum of **6f** (400 MHz, DMSO)


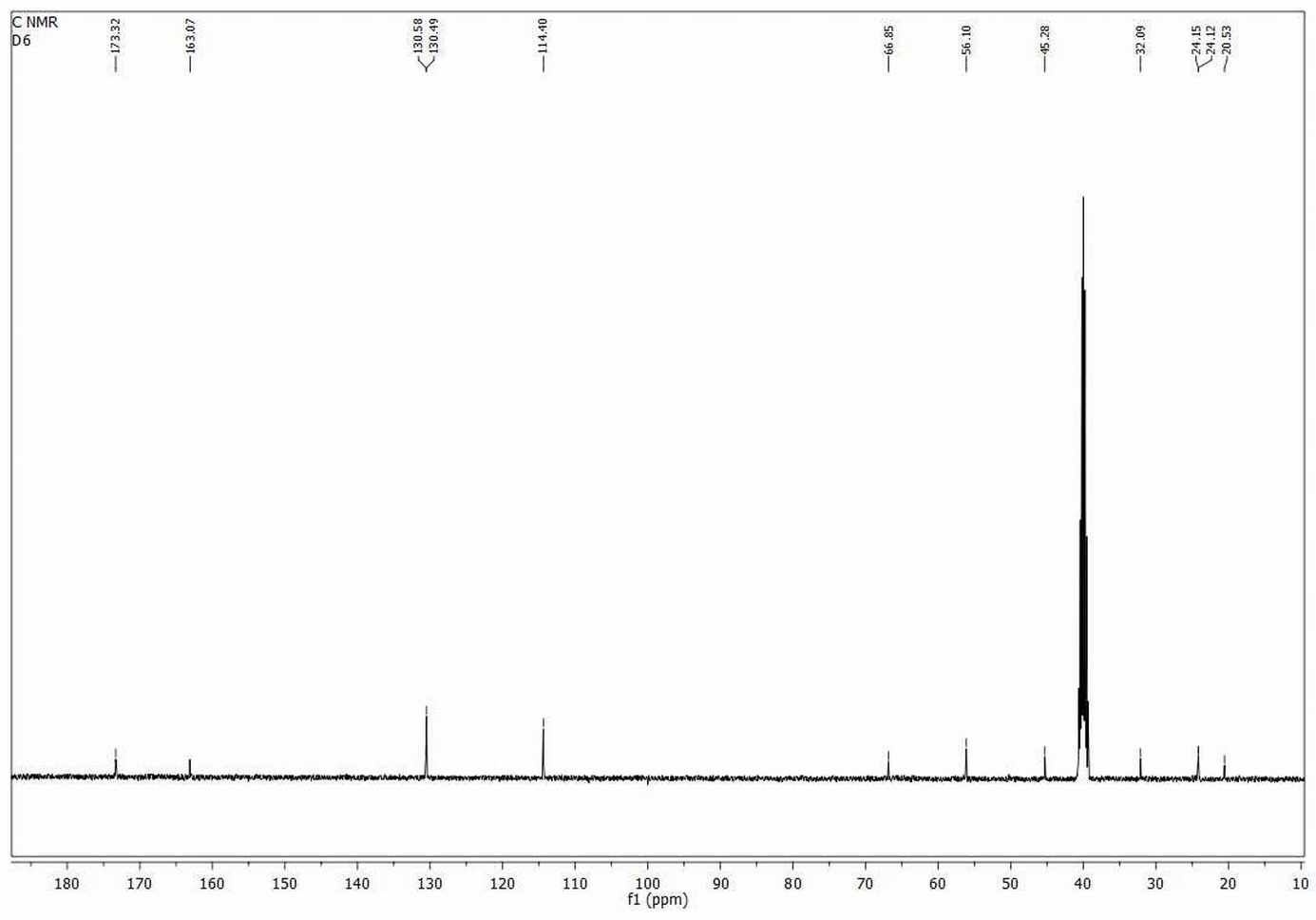


**Figure S19**. ^13^C-NMR spectrum of **6f** (101 MHz, DMSO)

**
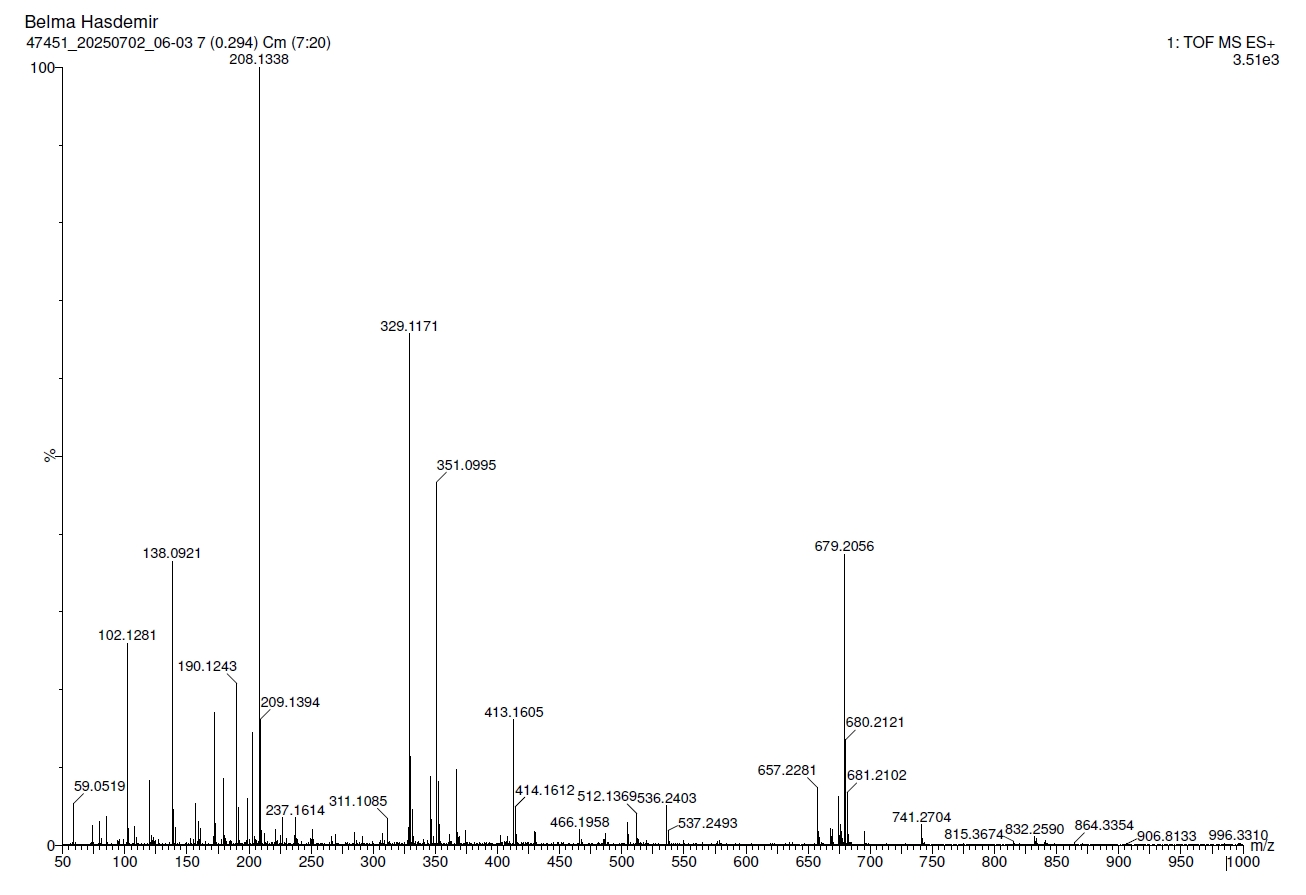
**

**Figure S20**. HRMS spectrum of **6f**


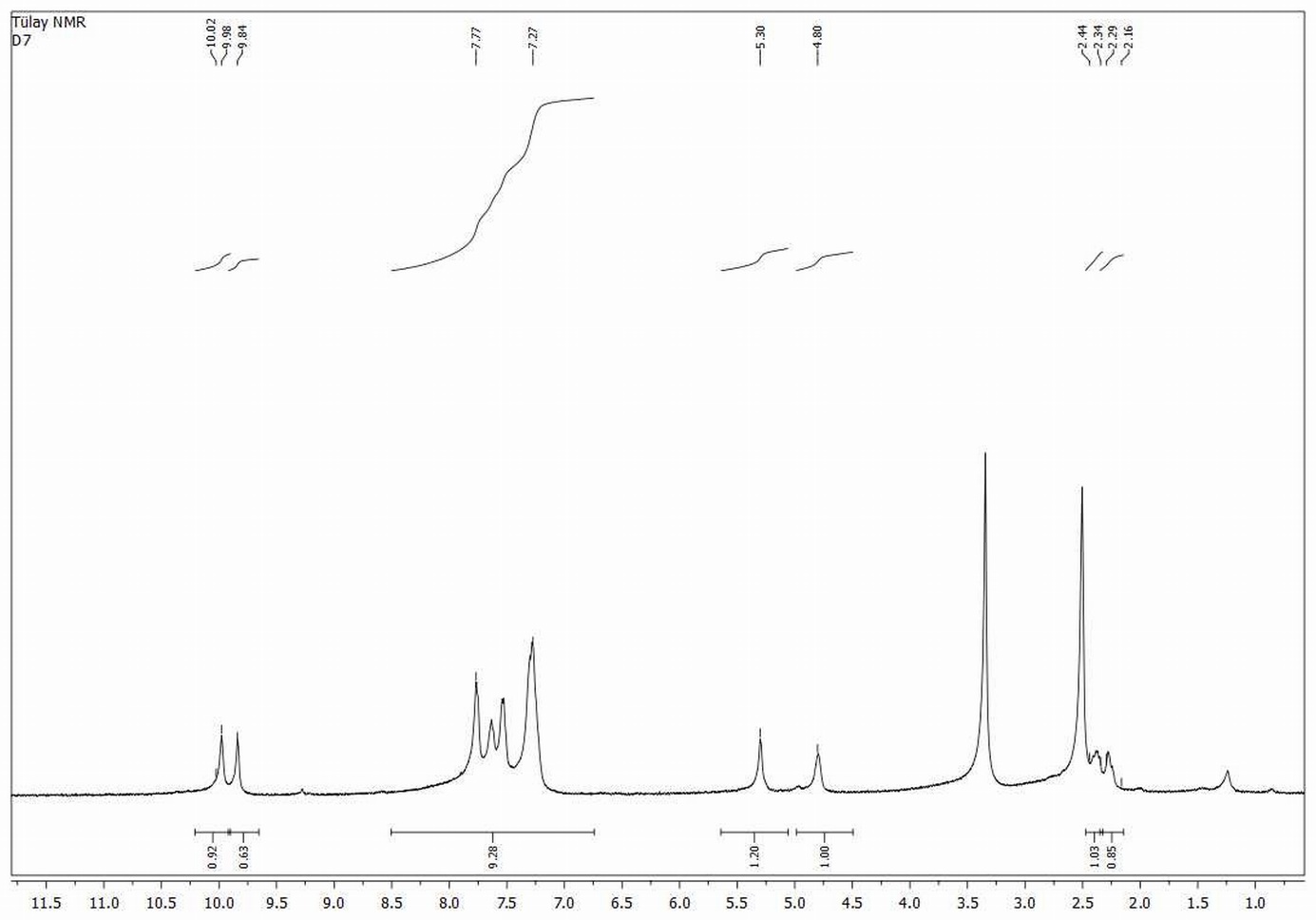


**Figure S21**. ^1^H-NMR spectrum of **6g** (400 MHz, DMSO)


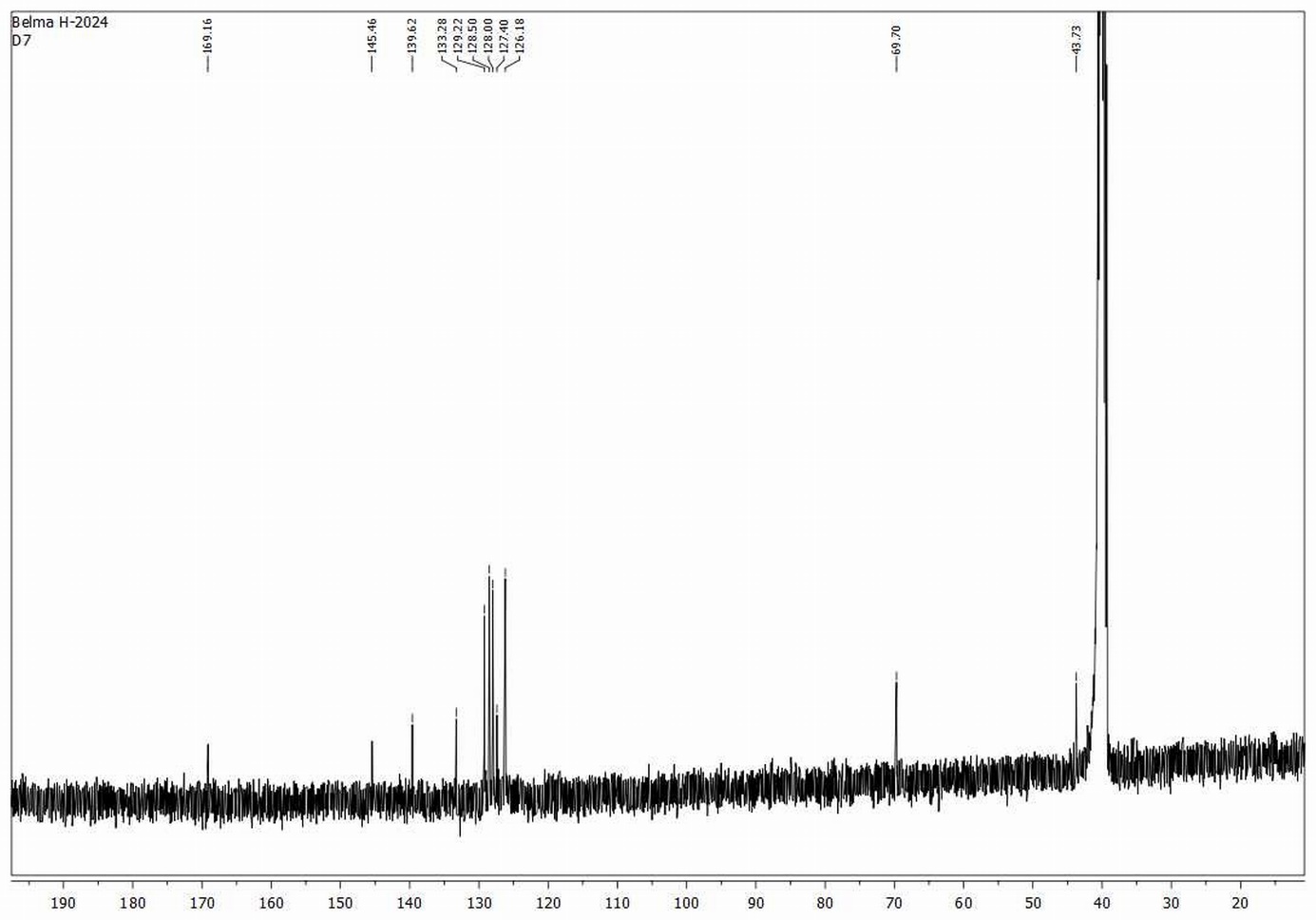


**Figure S22**. ^13^C-NMR spectrum of **6g** (101 MHz, DMSO)


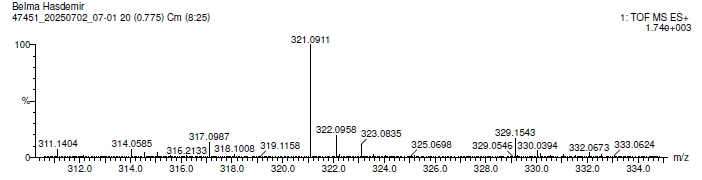


**Figure S23**. HRMS spectrum of **6g**


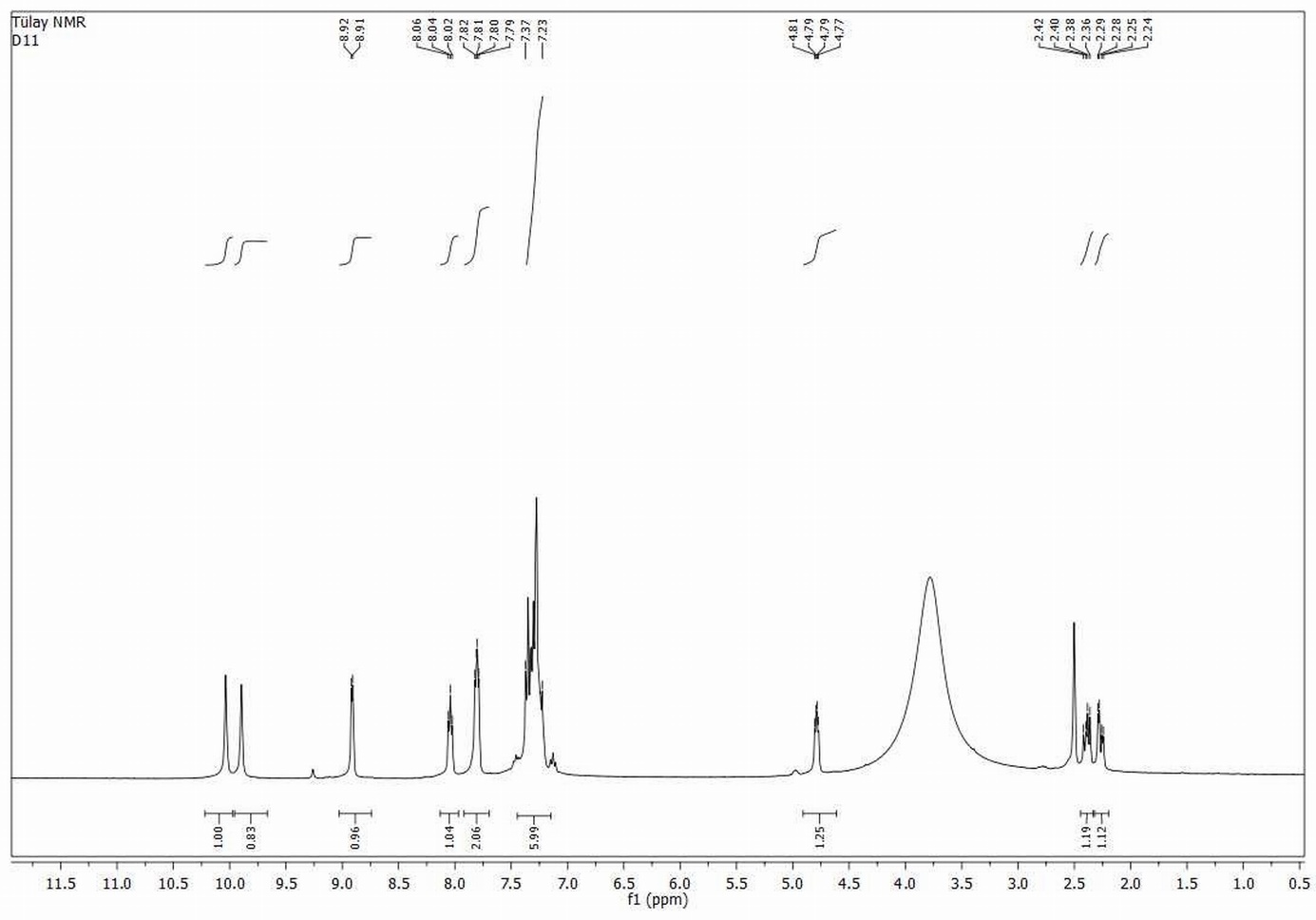


**Figure S24**. ^1^H-NMR spectrum of **6h** (400 MHz, DMSO)


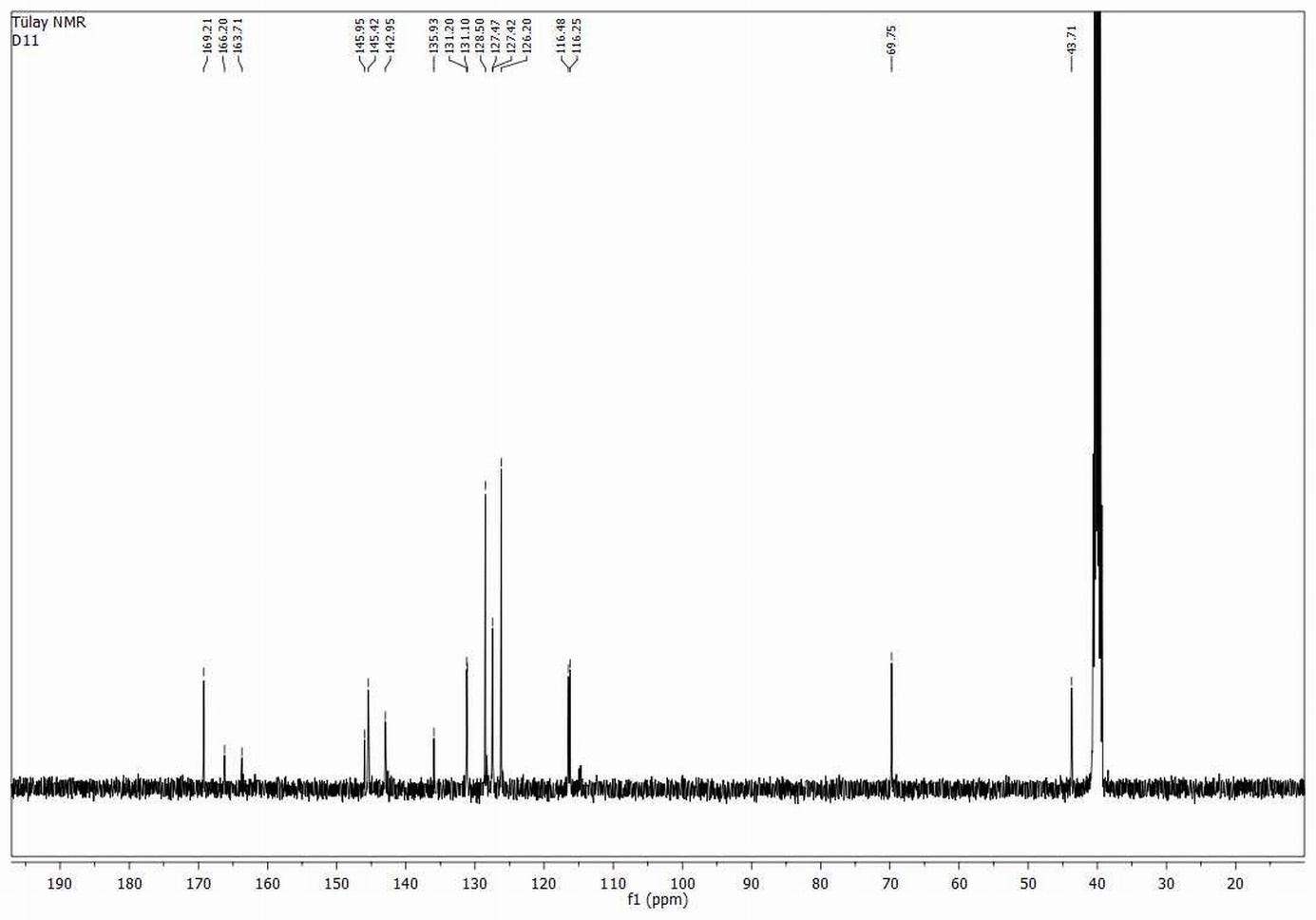


**Figure S25**. ^13^C-NMR spectrum of **6h** (101 MHz, DMSO)

**
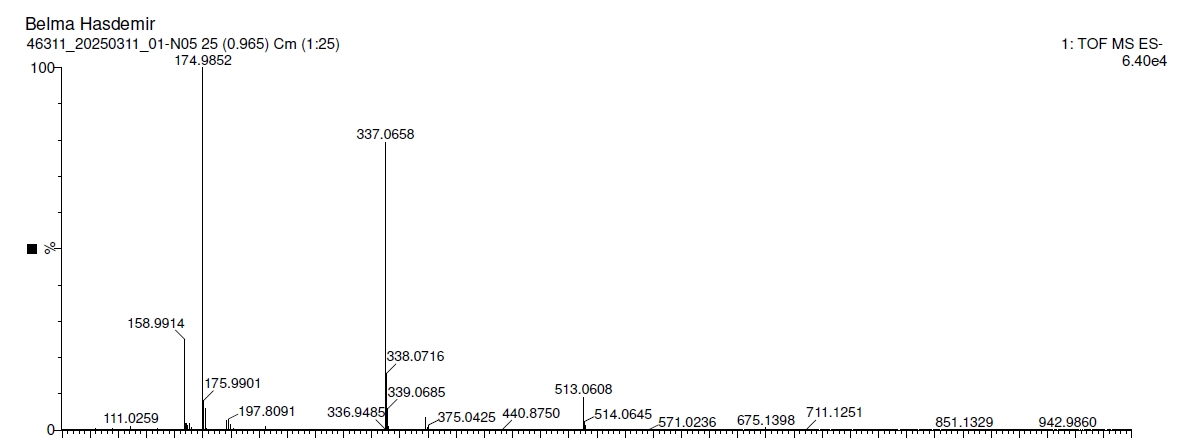
**

**Figure S26**. HRMS spectrum of **6h**


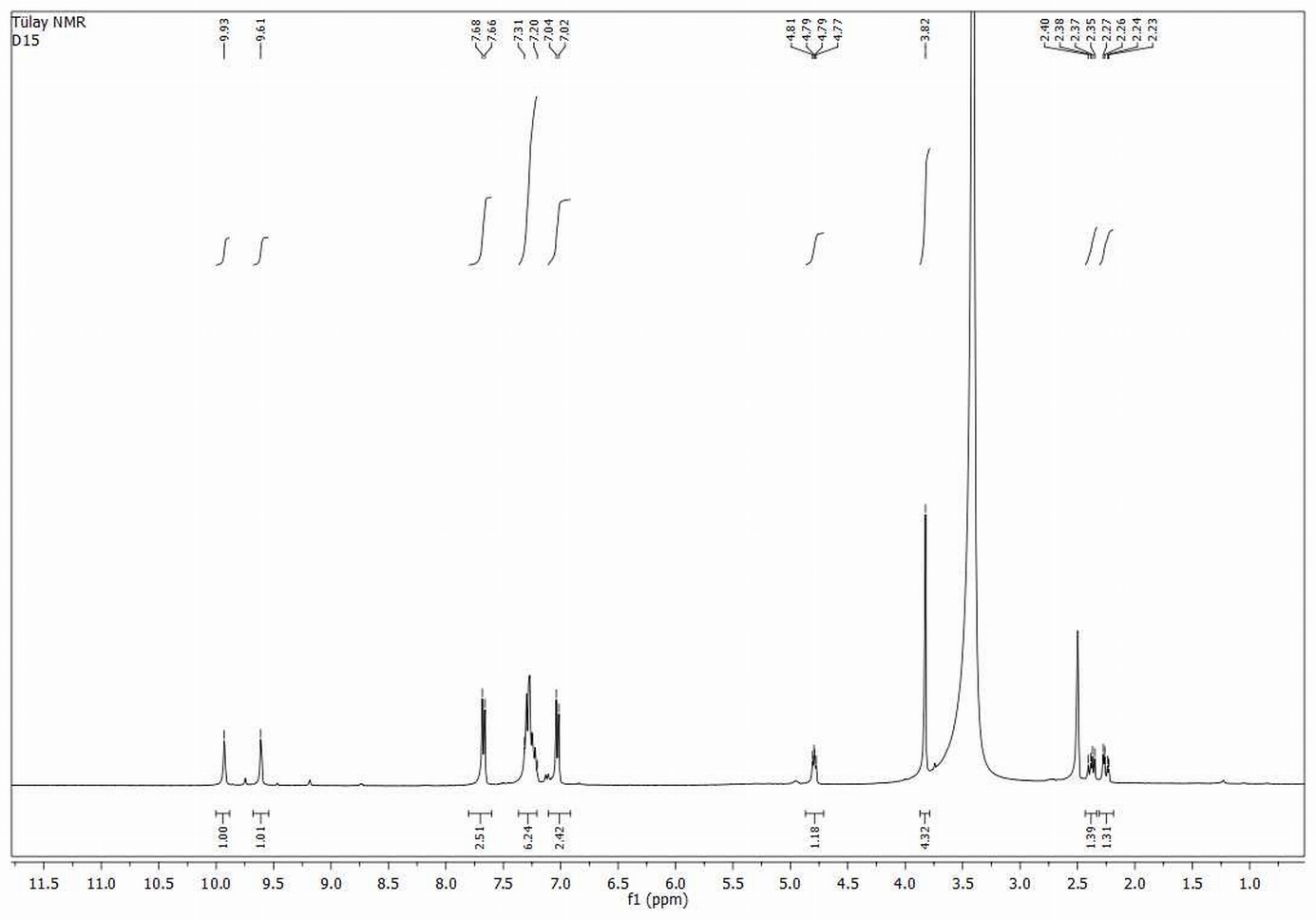


**Figure S27**. ^1^H-NMR spectrum of **6i** (400 MHz, DMSO)


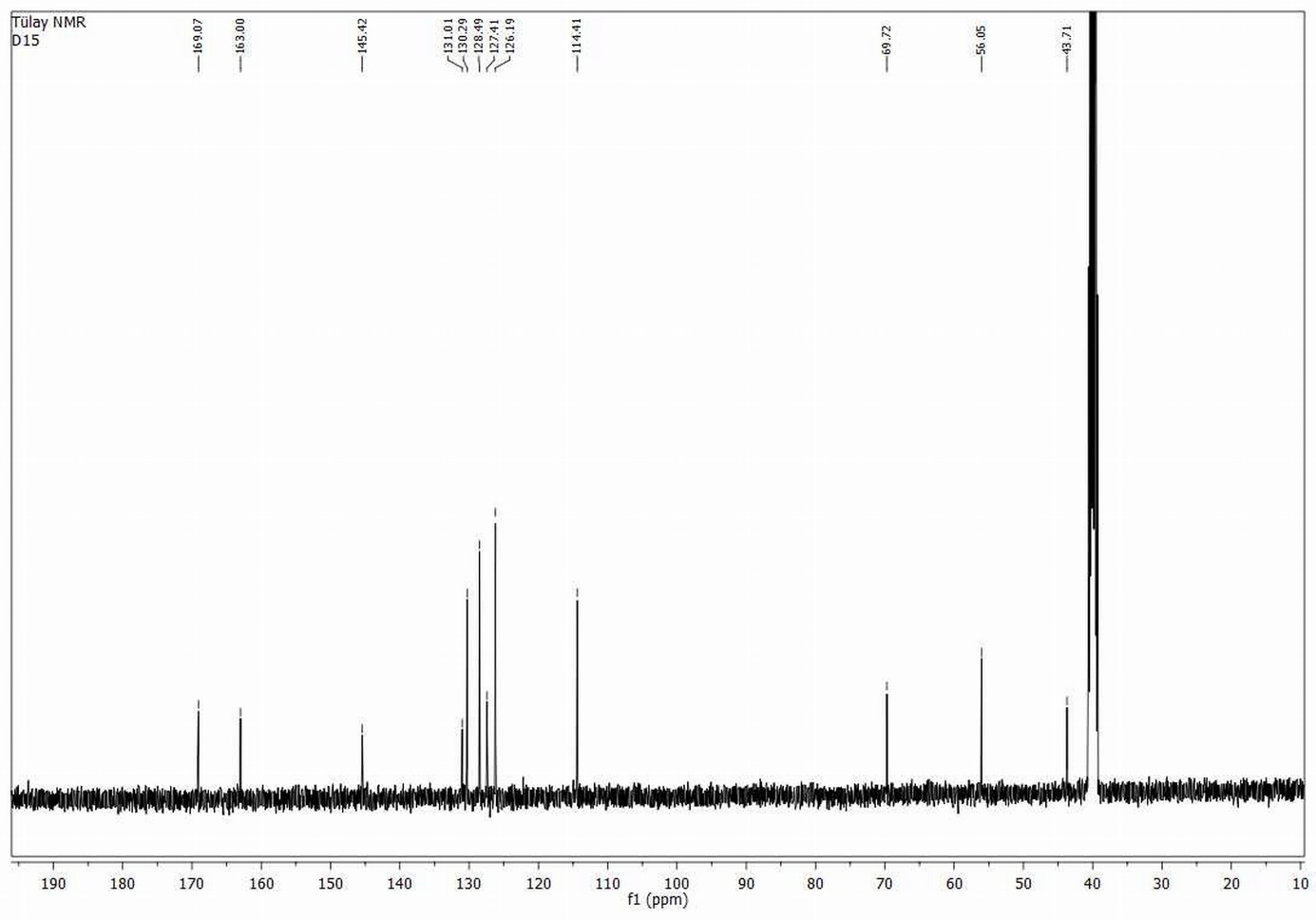


**Figure S28**. ^13^C-NMR spectrum of **6i** (101 MHz, DMSO)


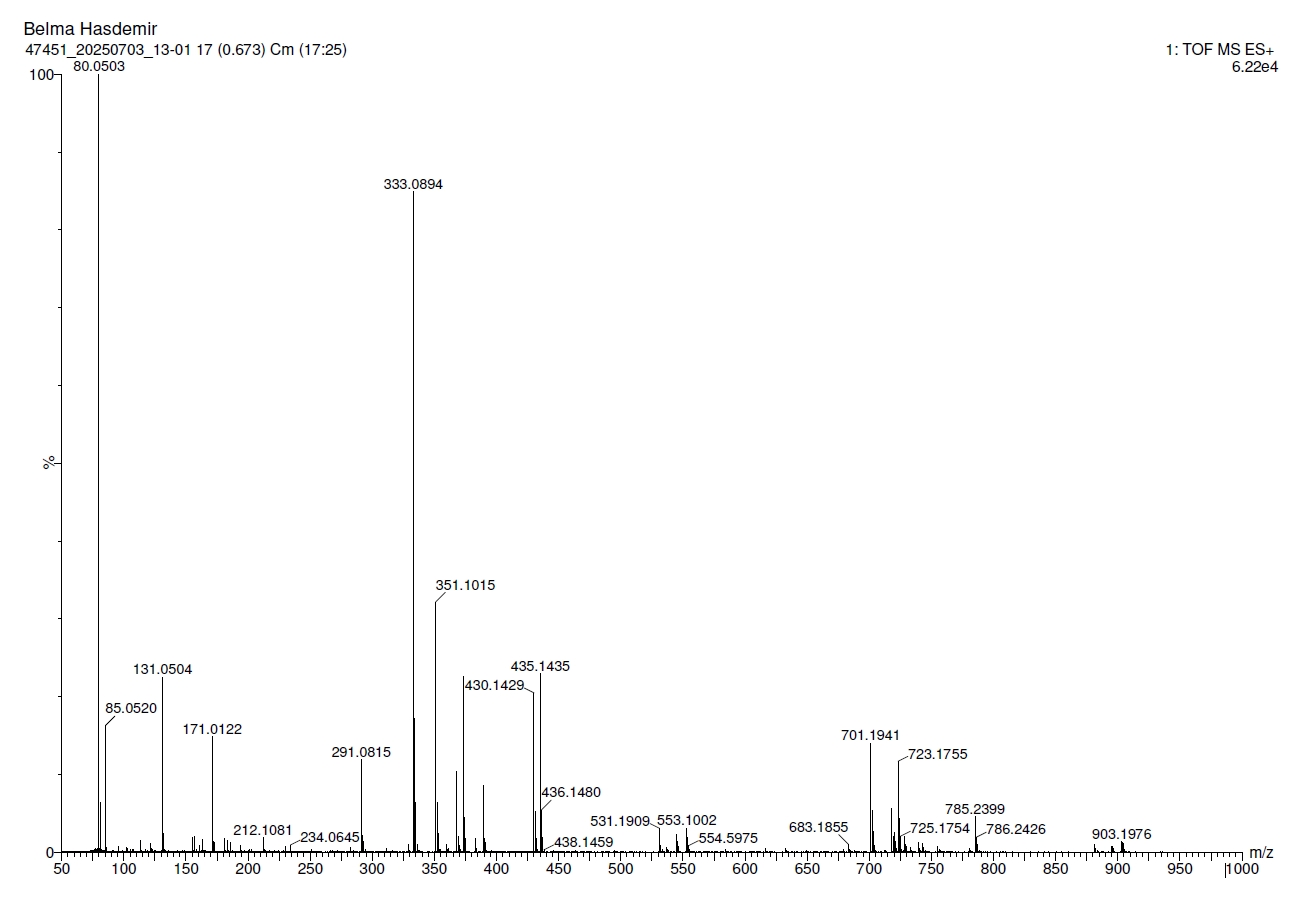


**Figure S29**. HRMS spectrum of **6i**


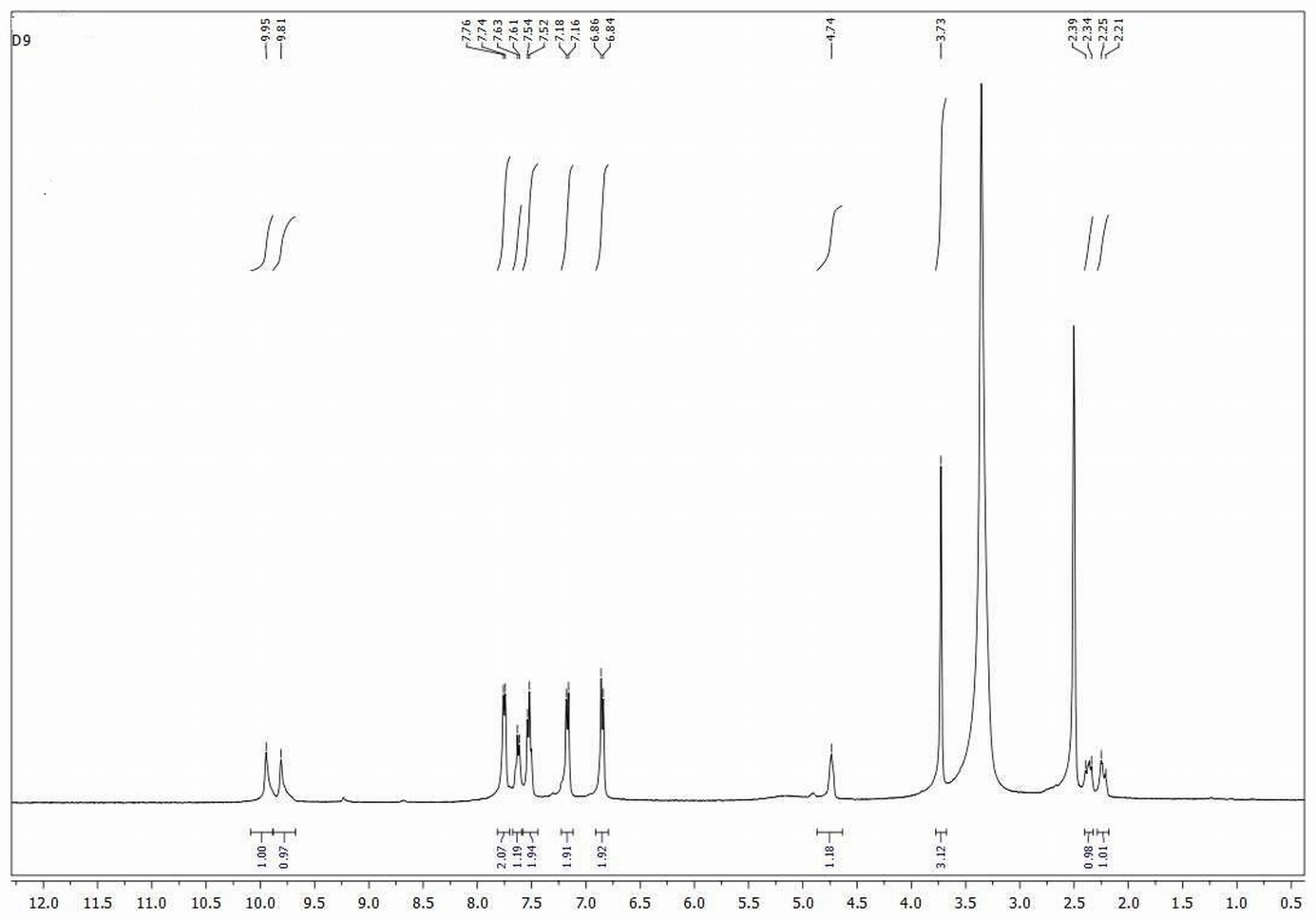


**Figure S30**. ^1^H-NMR spectrum of **6j** (400 MHz, DMSO)


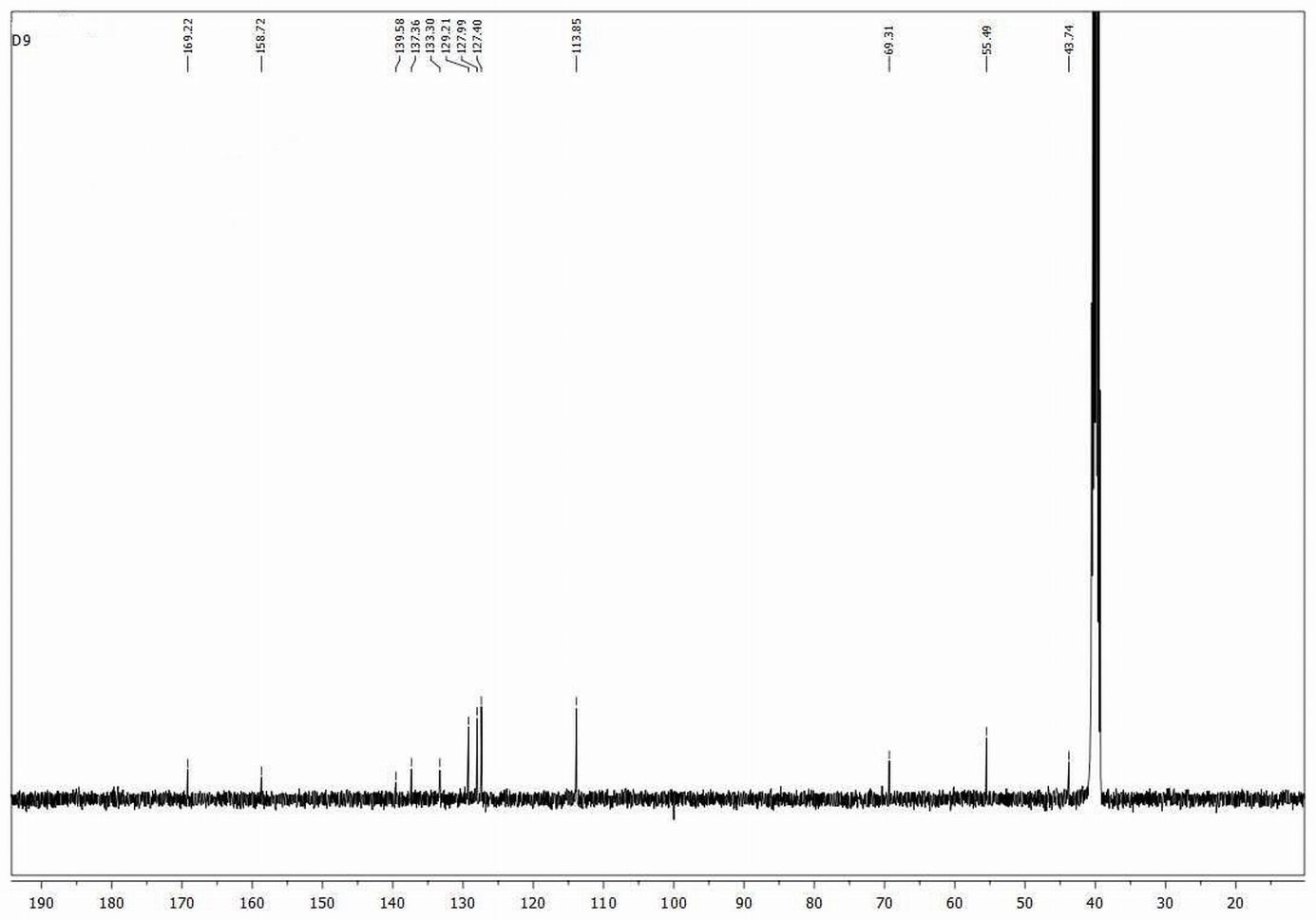


**Figure S31**. ^13^C-NMR spectrum of **6j** (101 MHz, DMSO)


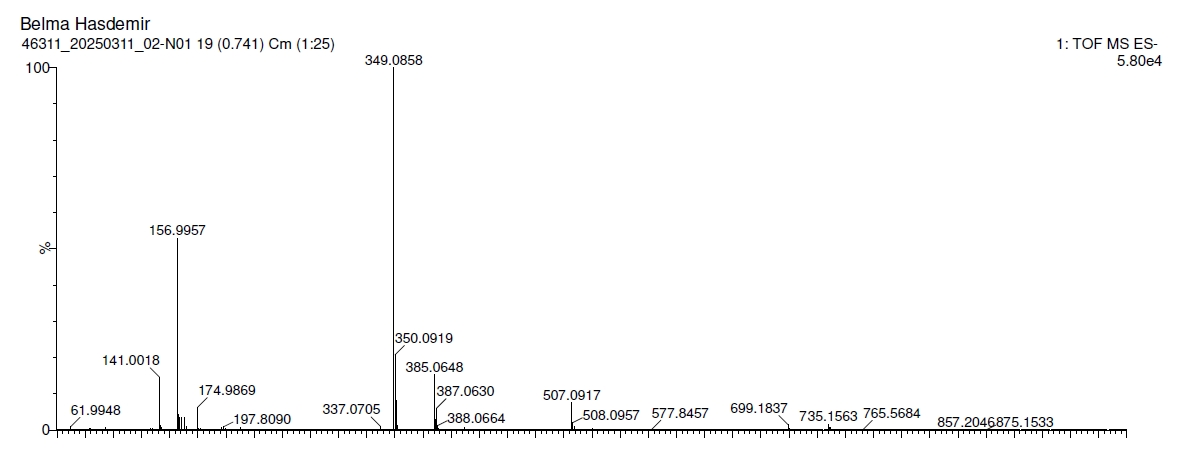


**Figure S32**. HRMS spectrum of **6j**


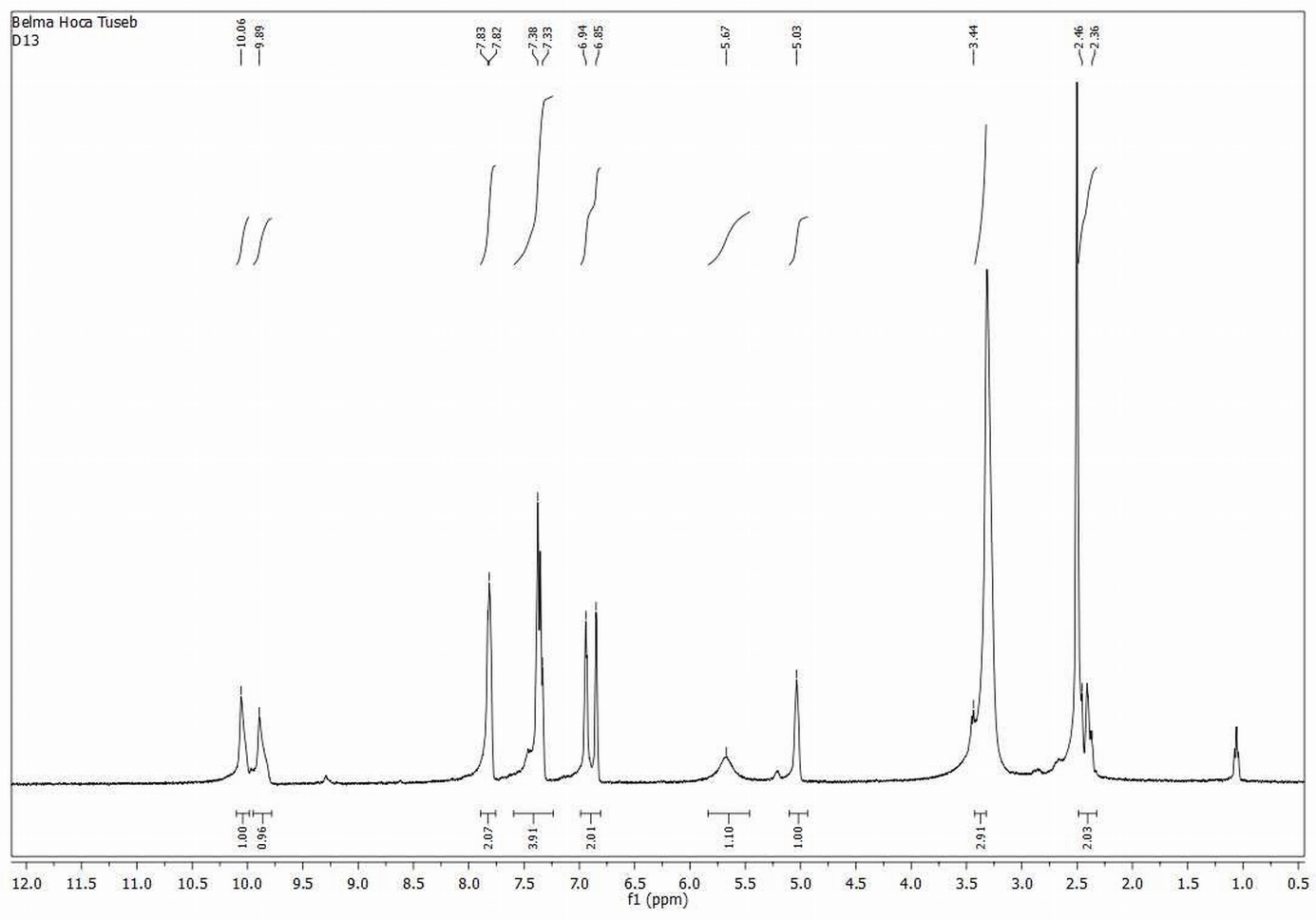


**Figure S33**. ^1^H-NMR spectrum of **6k** (400 MHz, DMSO)


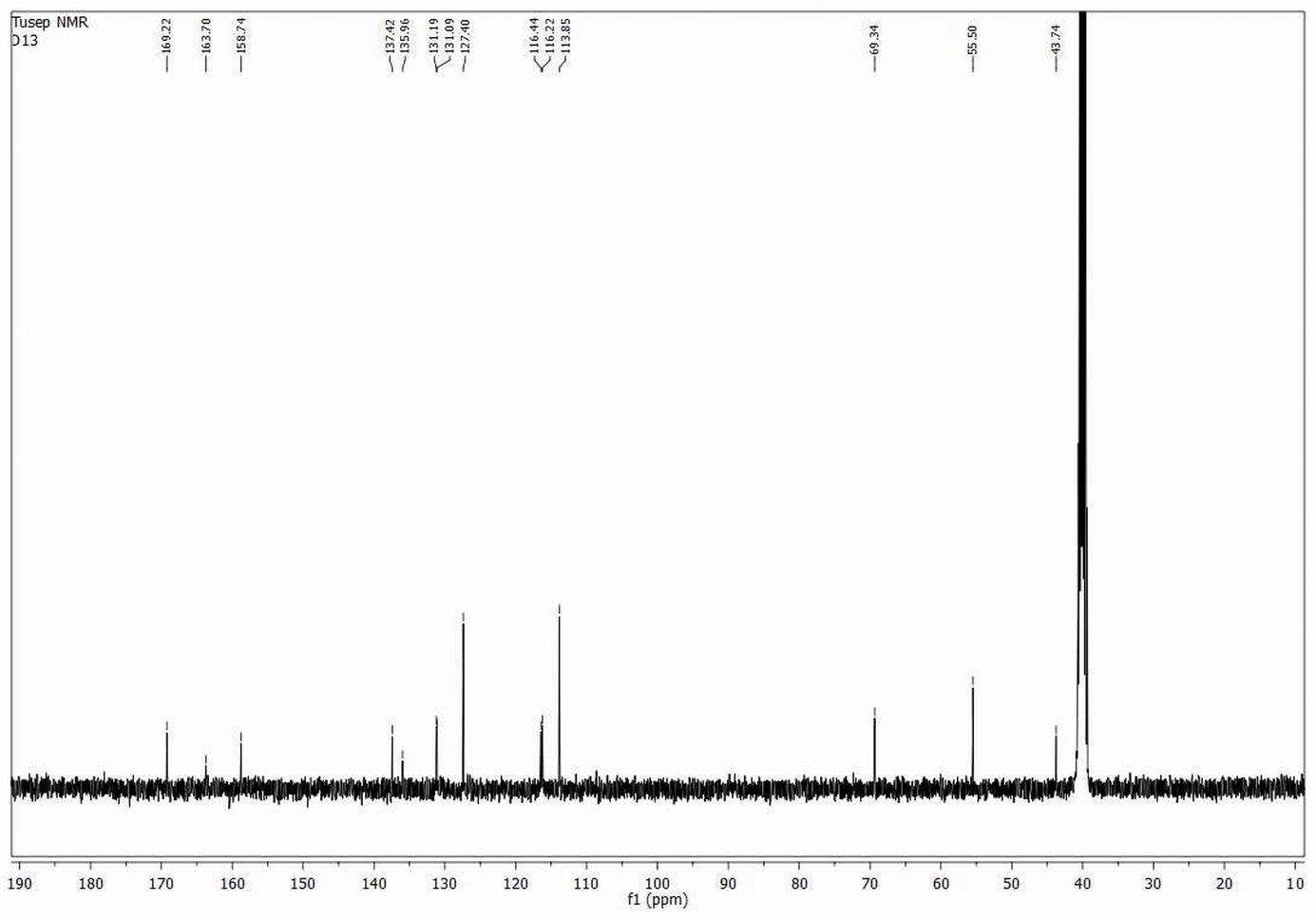


**Figure S34**. ^13^C-NMR spectrum of **6k** (101 MHz, DMSO)


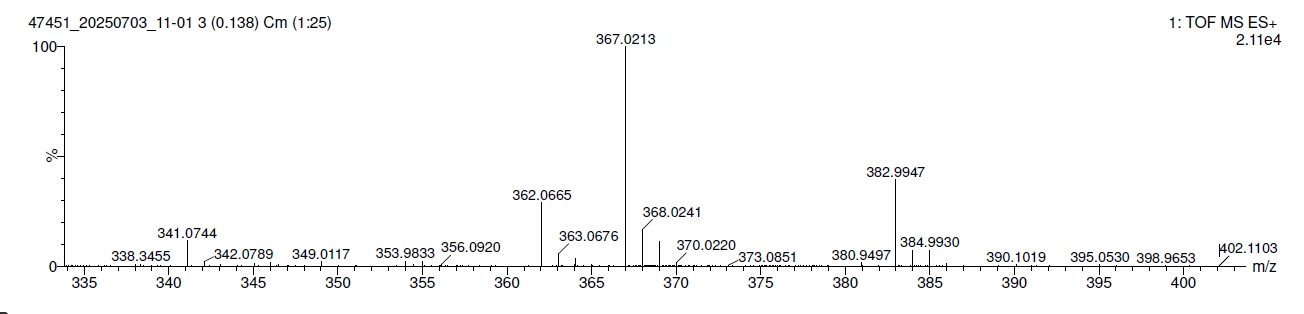


**Figure S35**. HRMS spectrum of **6k**


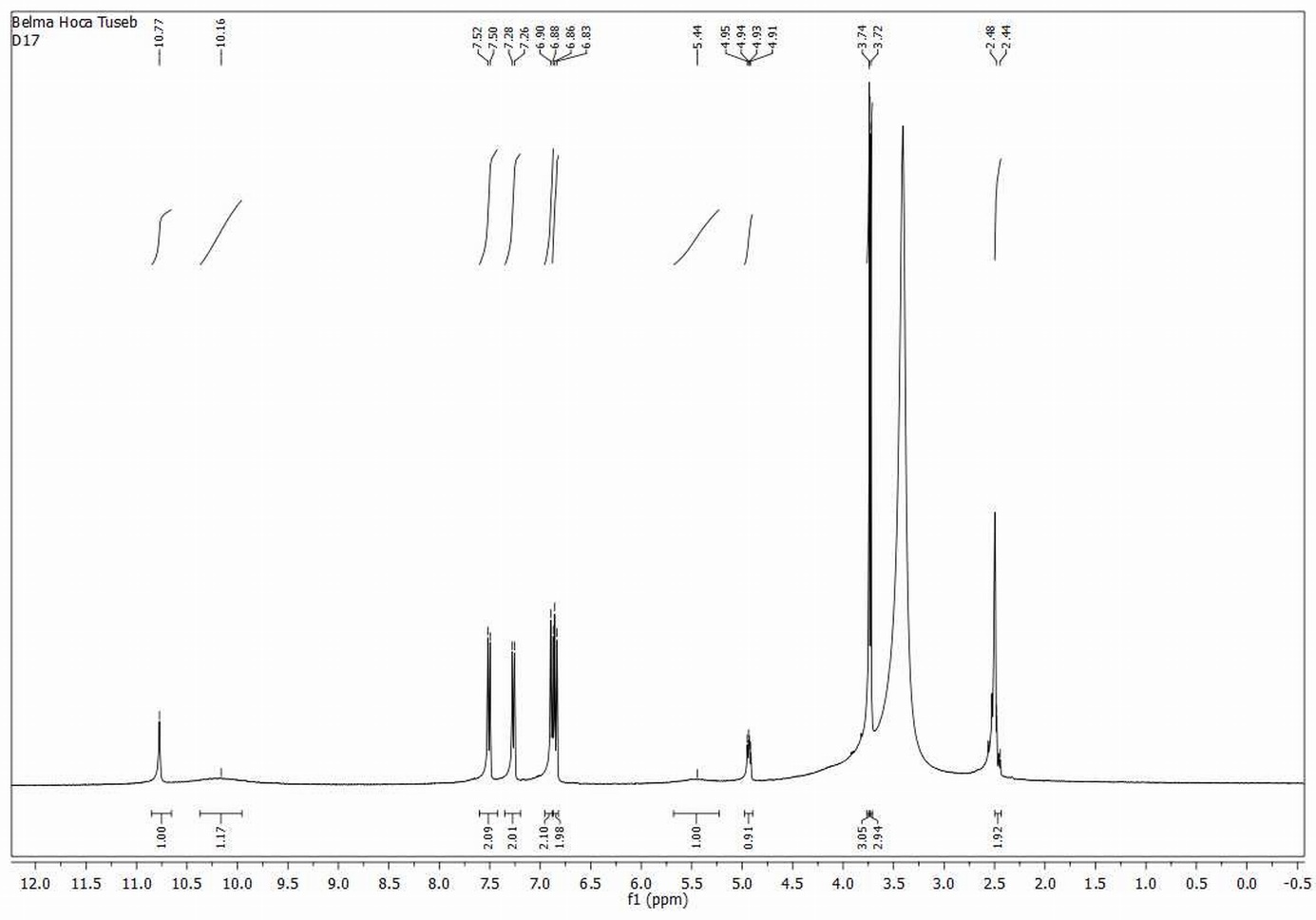


**Figure S36**. ^1^H-NMR spectrum of **6l** (400 MHz, DMSO)

**
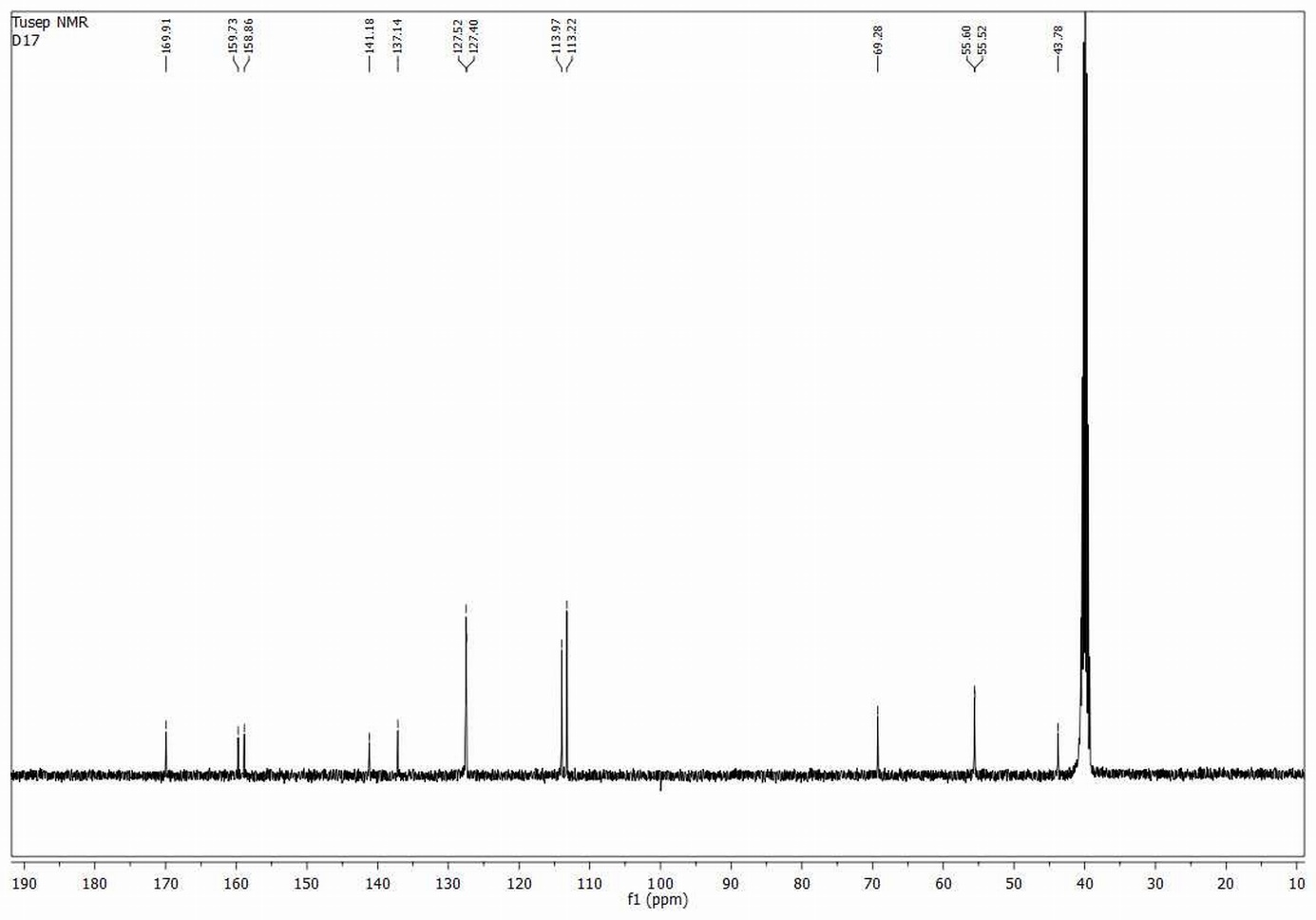
**

**Figure S37**. ^13^C-NMR spectrum of **6l** (101 MHz, DMSO)


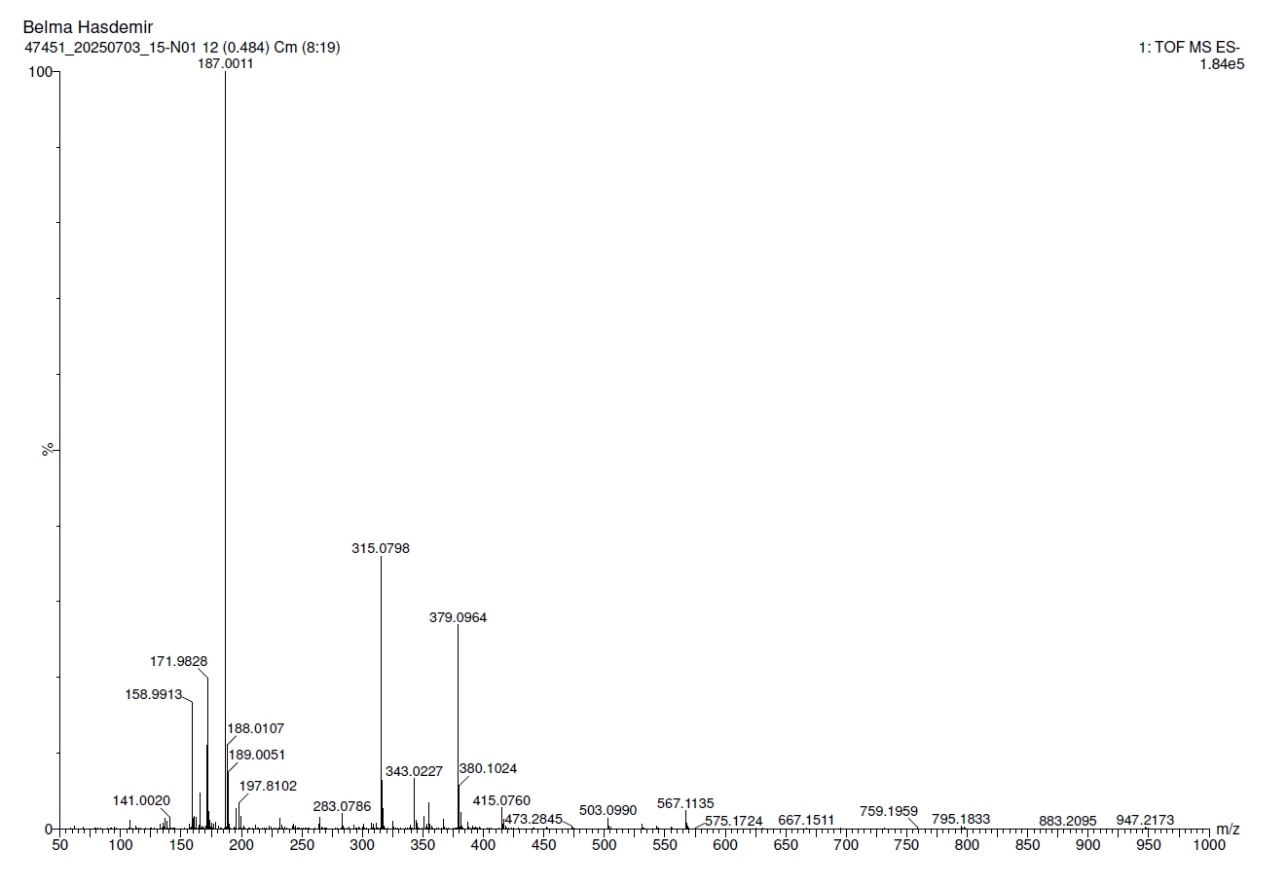


**Figure S38**. HRMS spectrum of **6l**


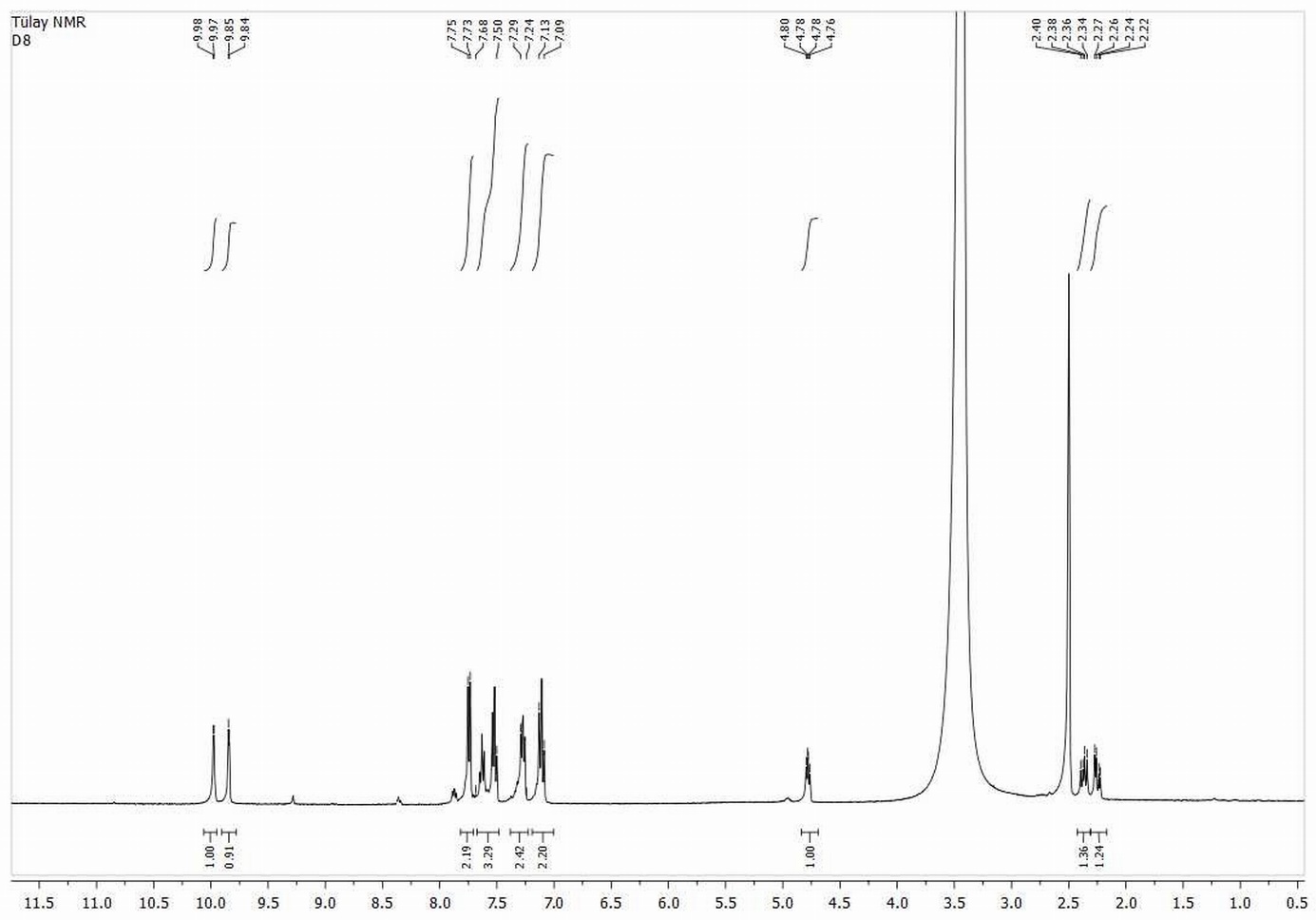


**Figure S39**. ^1^H-NMR spectrum of **6m** (400 MHz, DMSO)

**Figure S40**. ^13^C-NMR spectrum of **6m** (101 MHz, DMSO)


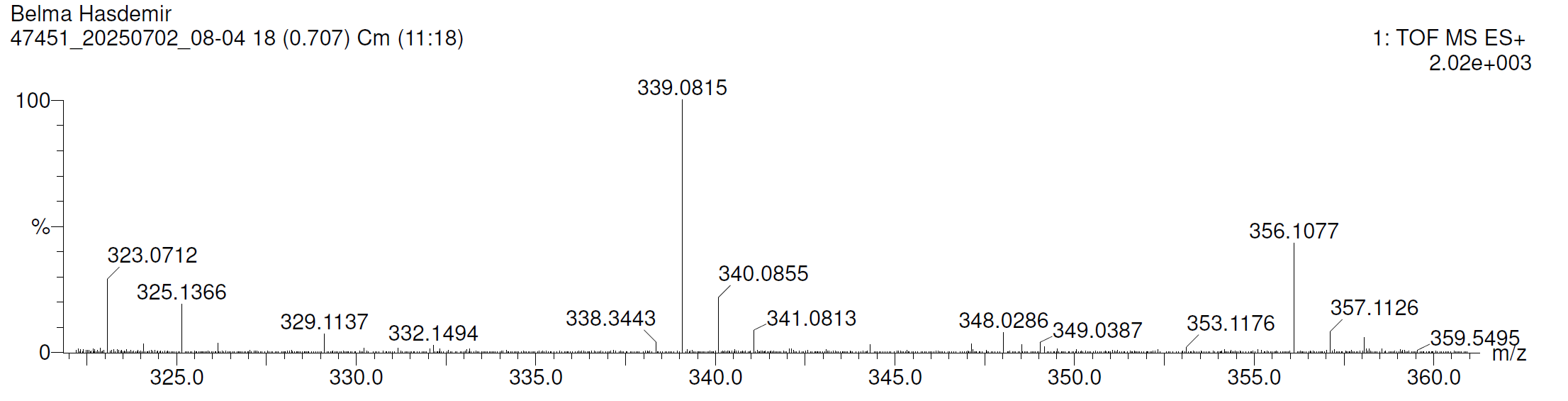


**Figure S41**. HRMS spectrum of **6m**


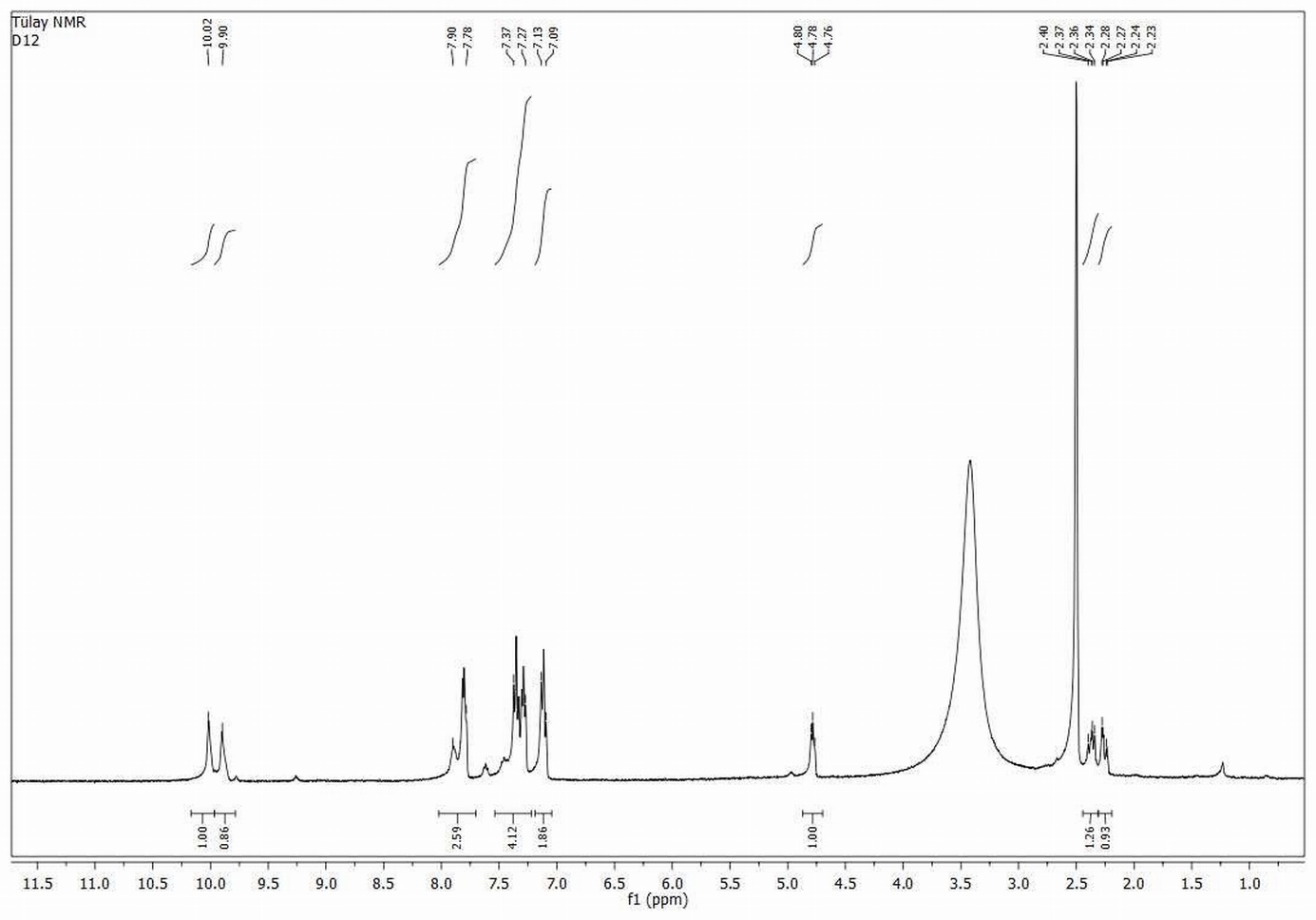


**Figure S42**. ^1^H-NMR spectrum of **6n** (400 MHz, DMSO)


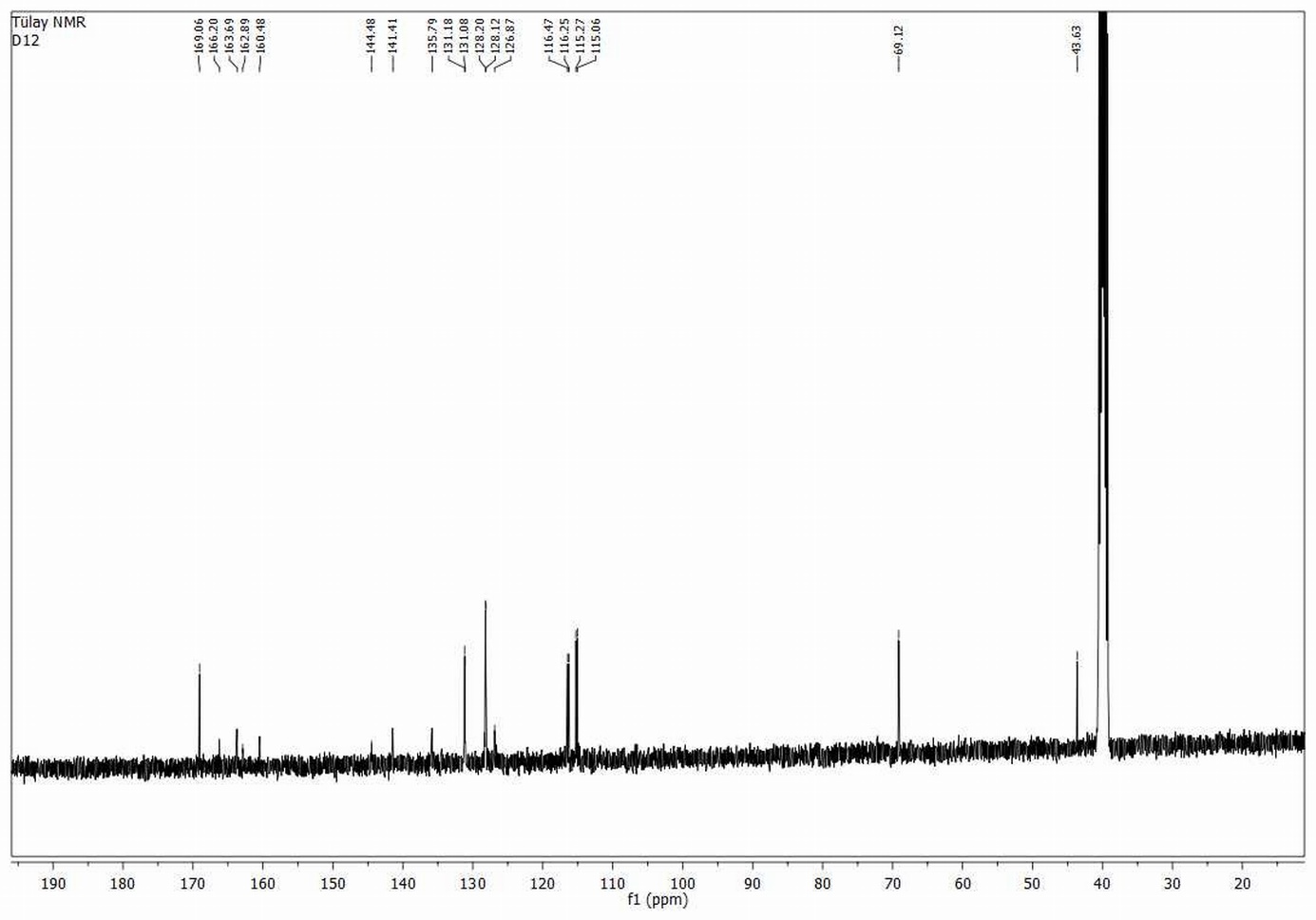


**Figure S43**. ^13^C-NMR spectrum of **6n** (101 MHz, DMSO)


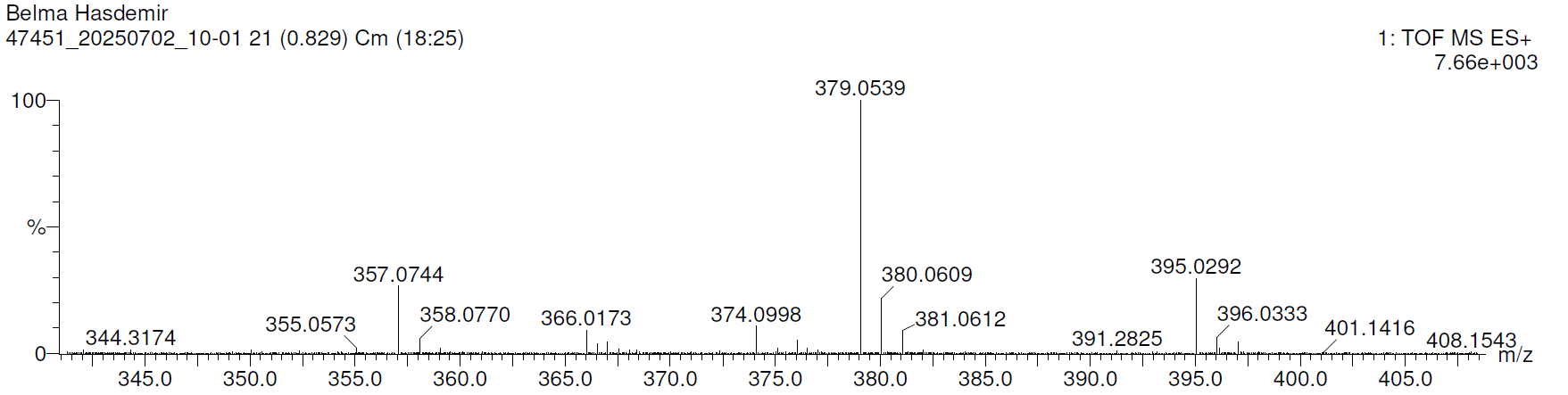


**Figure S44**. HRMS spectrum of **6n**


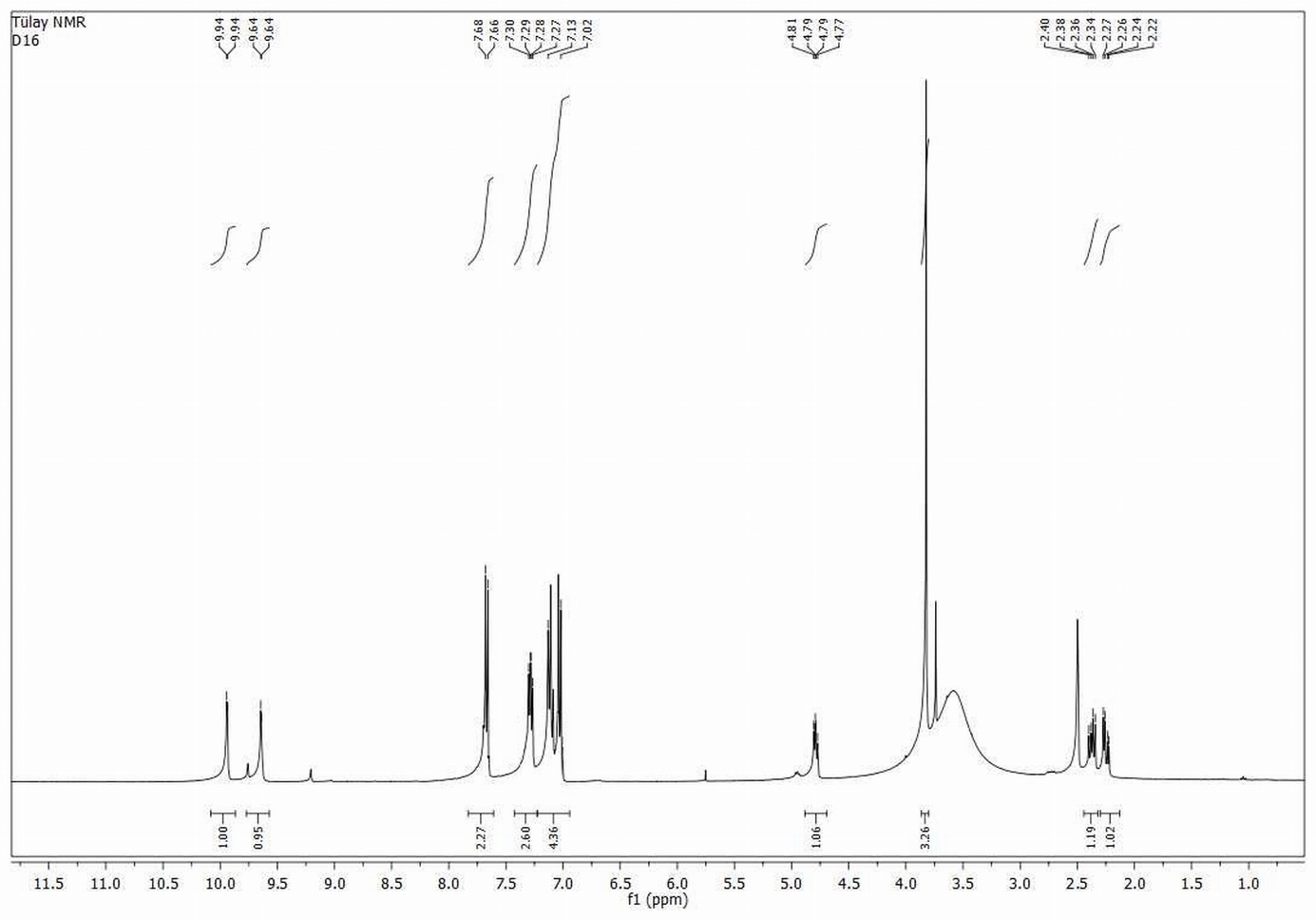


**Figure S45**. ^1^H-NMR spectrum of **6o** (400 MHz, DMSO)

**
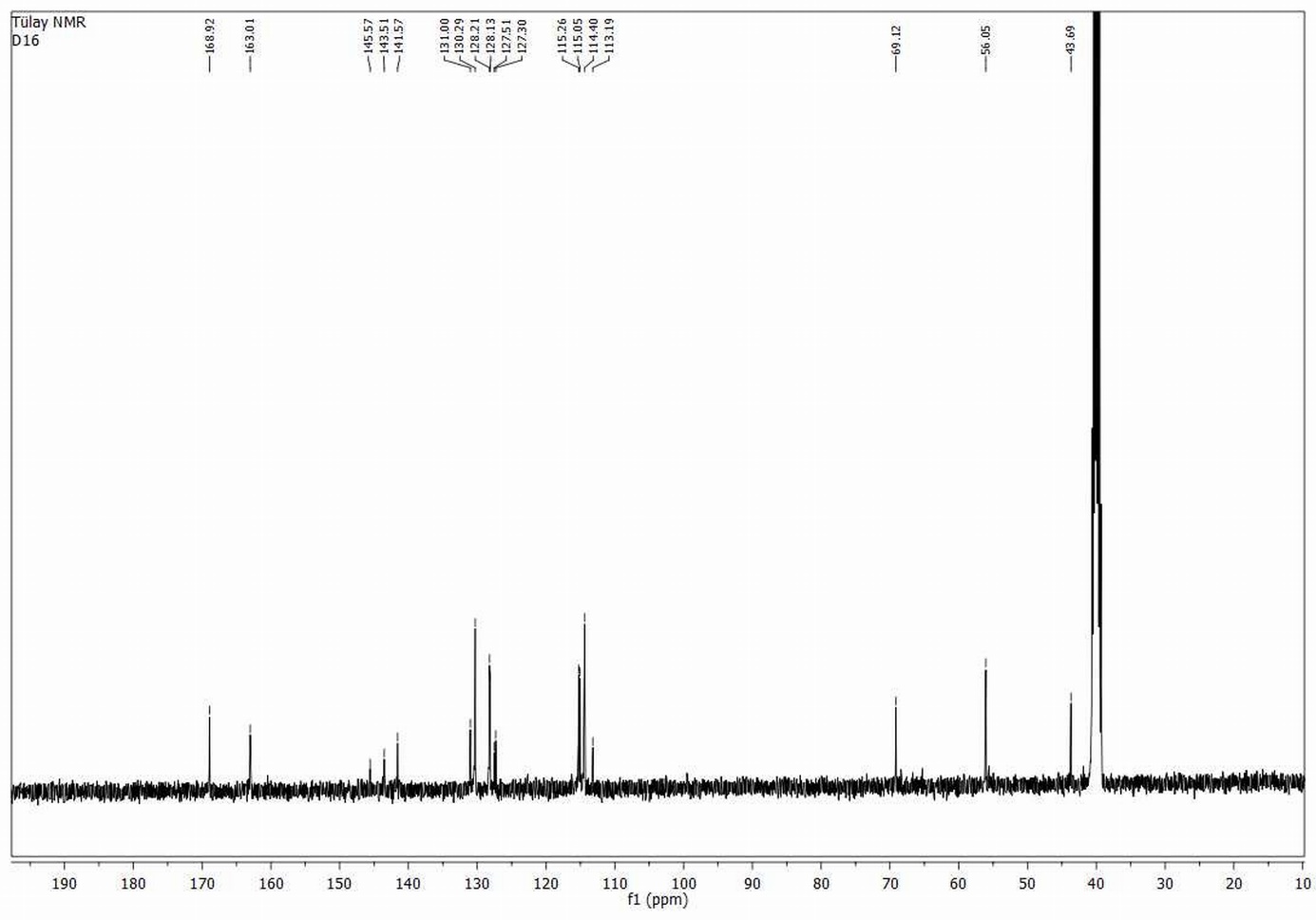
**

**Figure S46**. ^13^C-NMR spectrum of **6o** (101 MHz, DMSO)


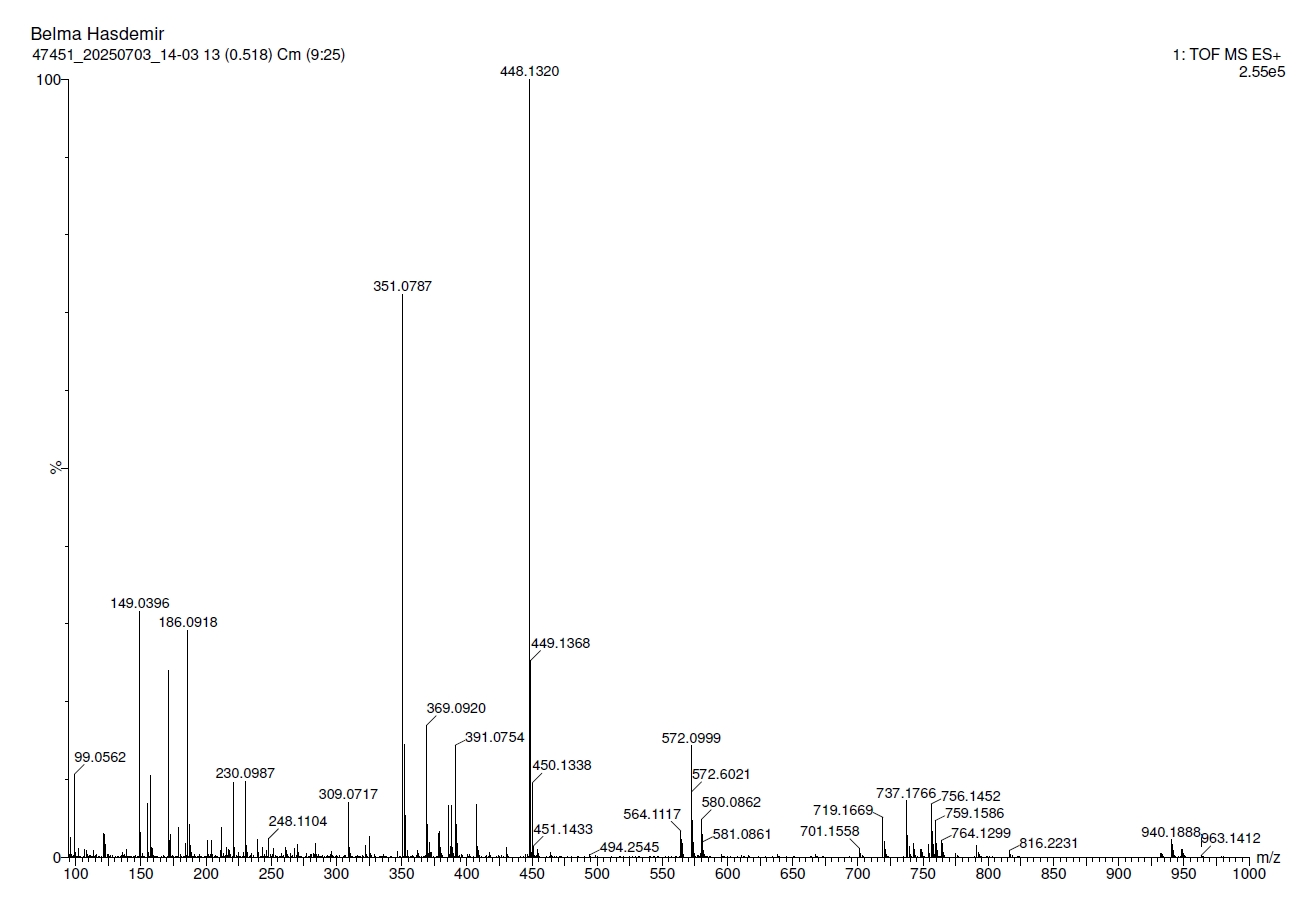


**Figure S47**. HRMS spectrum of **6o**


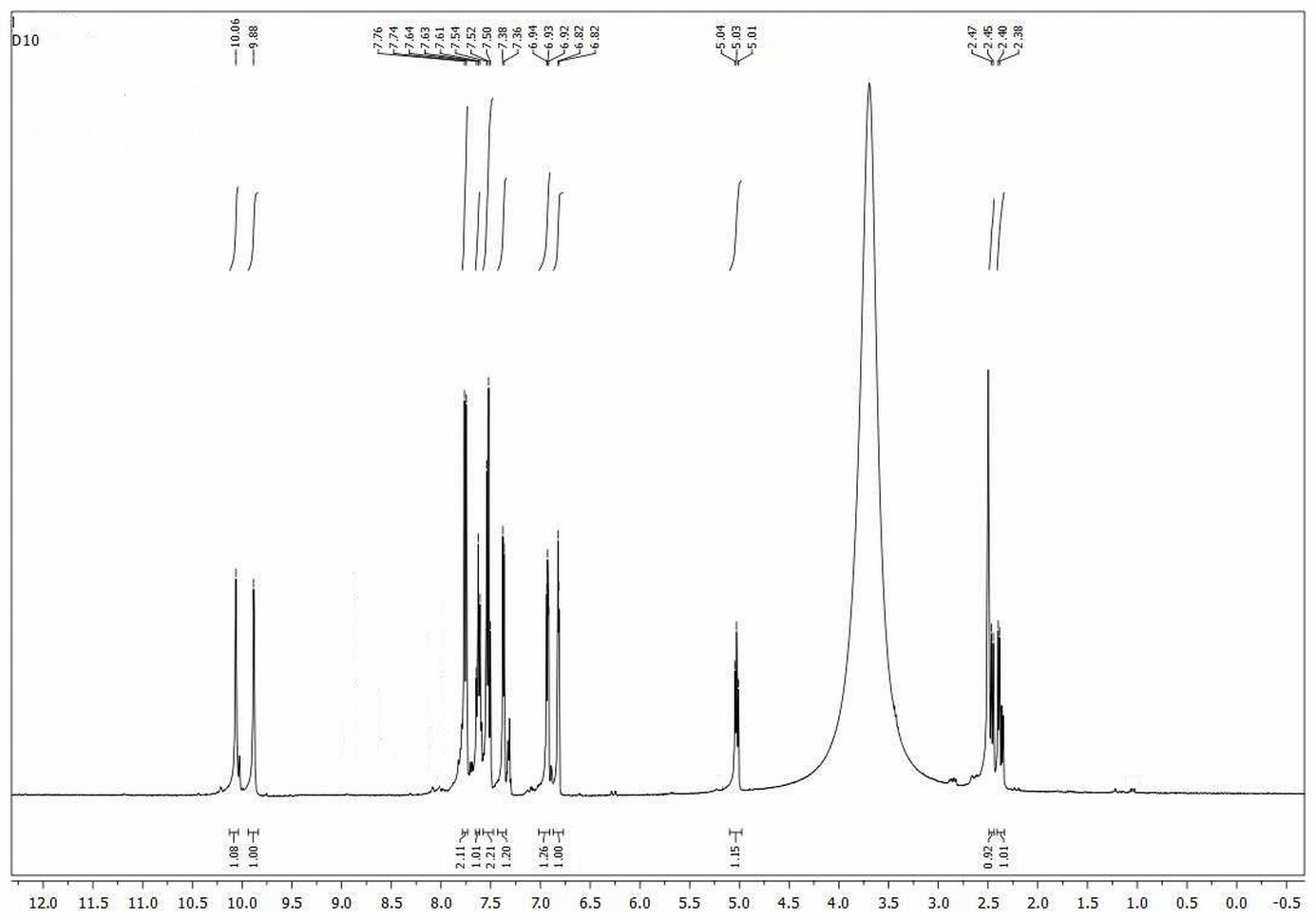


**Figure S48**. ^1^H-NMR spectrum of **6p** (400 MHz, DMSO)


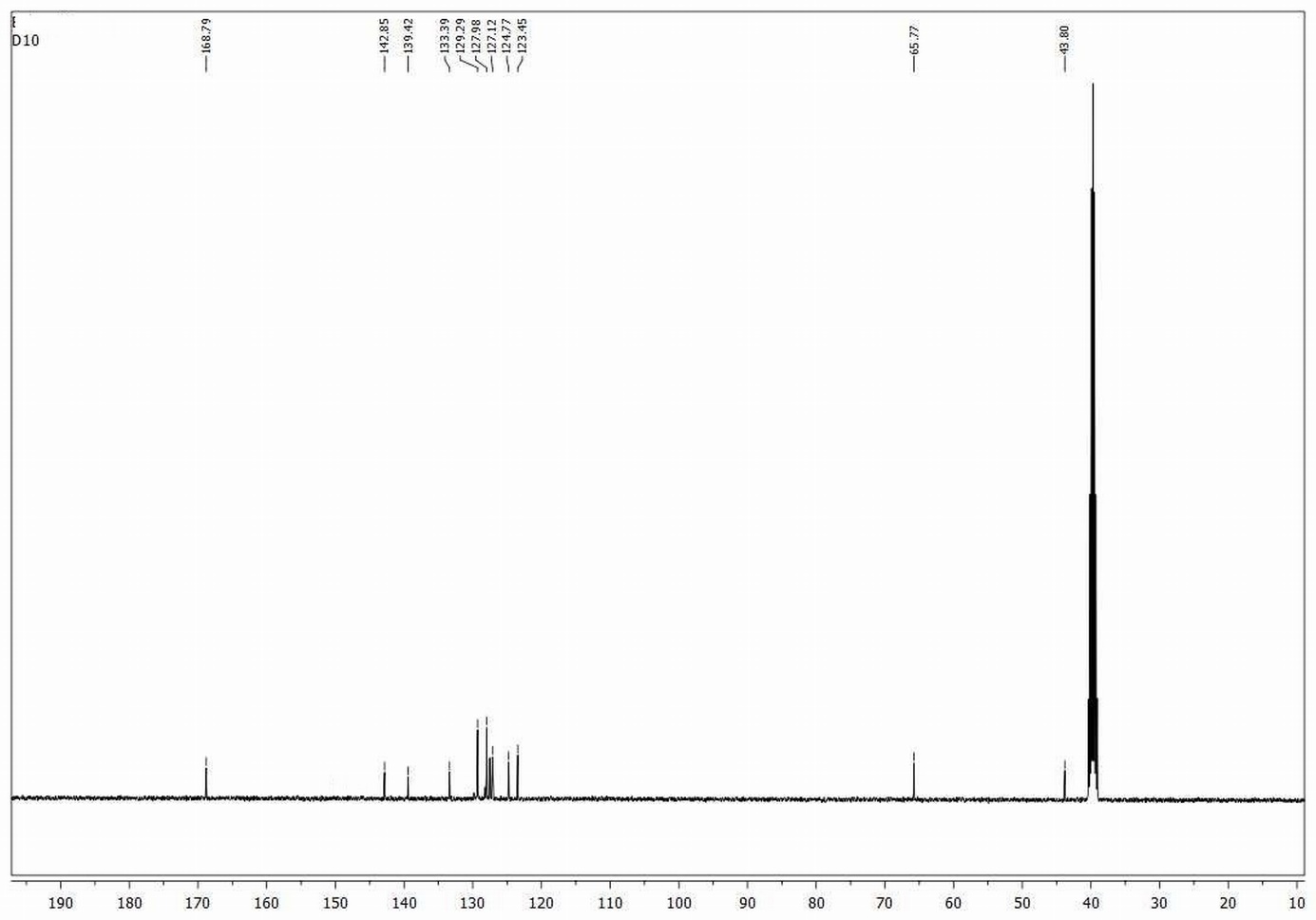


**Figure S49**. ^13^C-NMR spectrum of **6p** (101 MHz, DMSO)


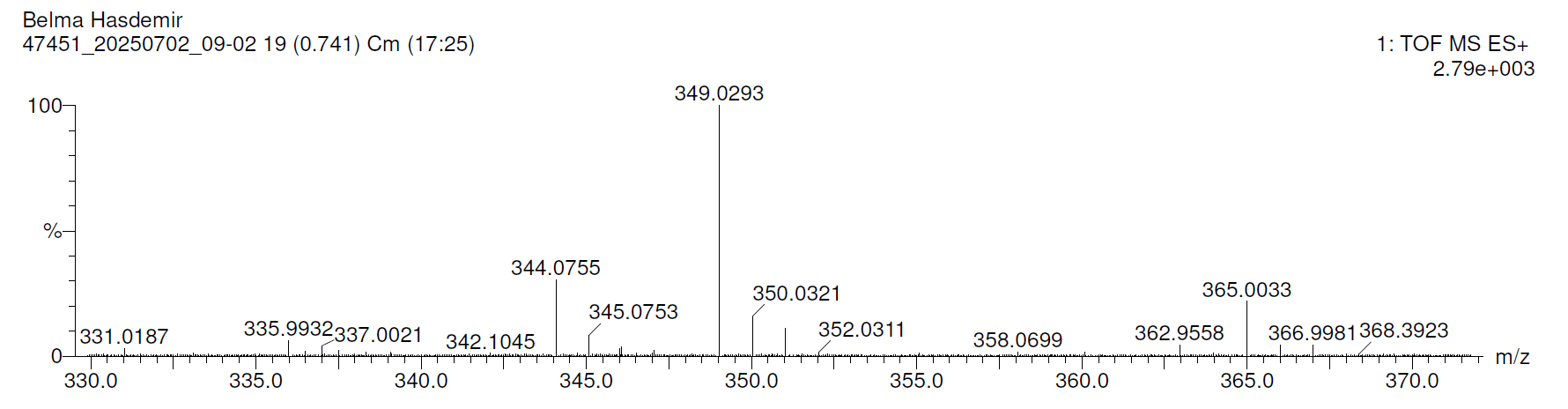


**Figure S50**. HRMS spectrum of **6p**


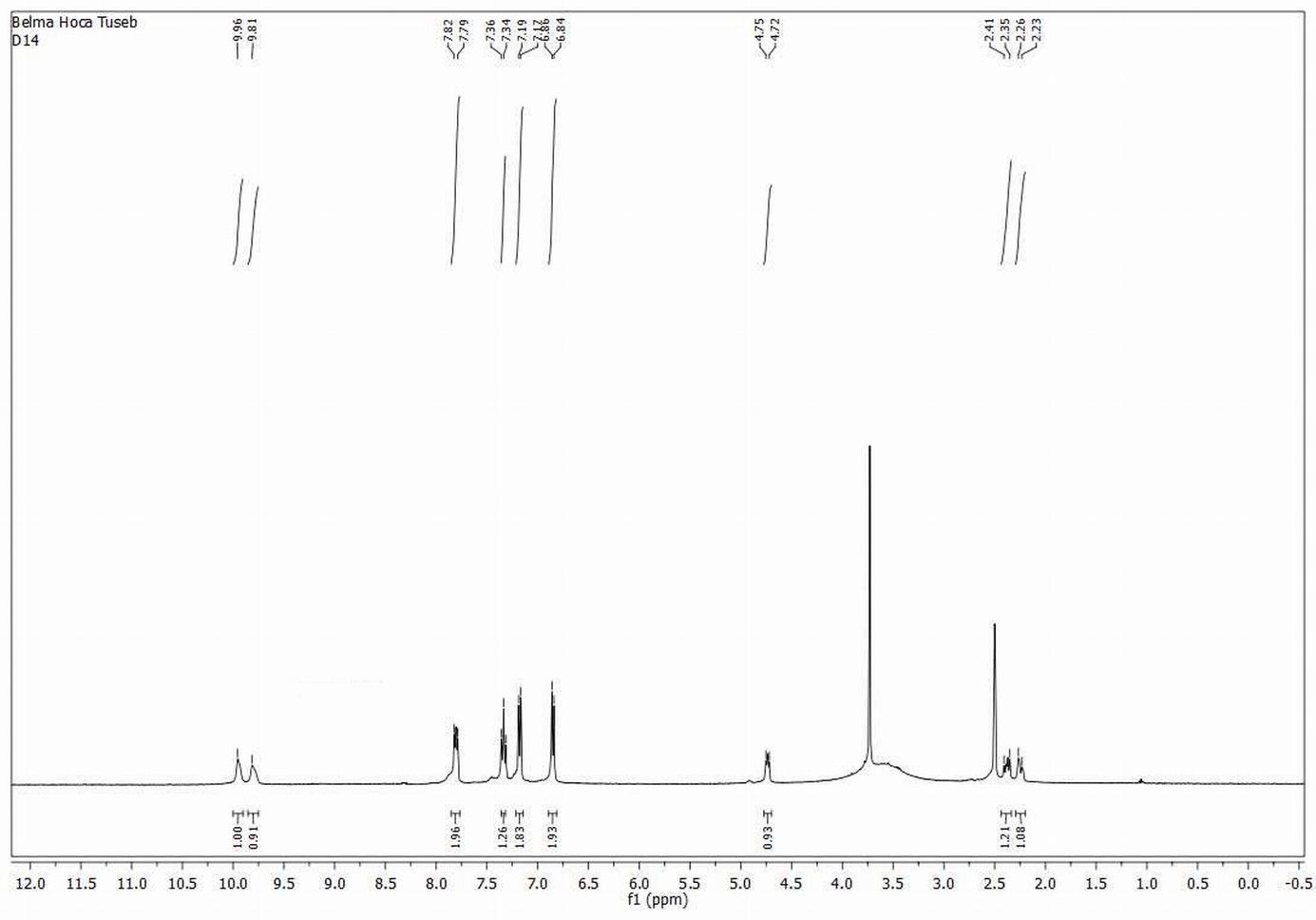


**Figure S51**. ^1^H-NMR spectrum of **6q** (400 MHz, DMSO)


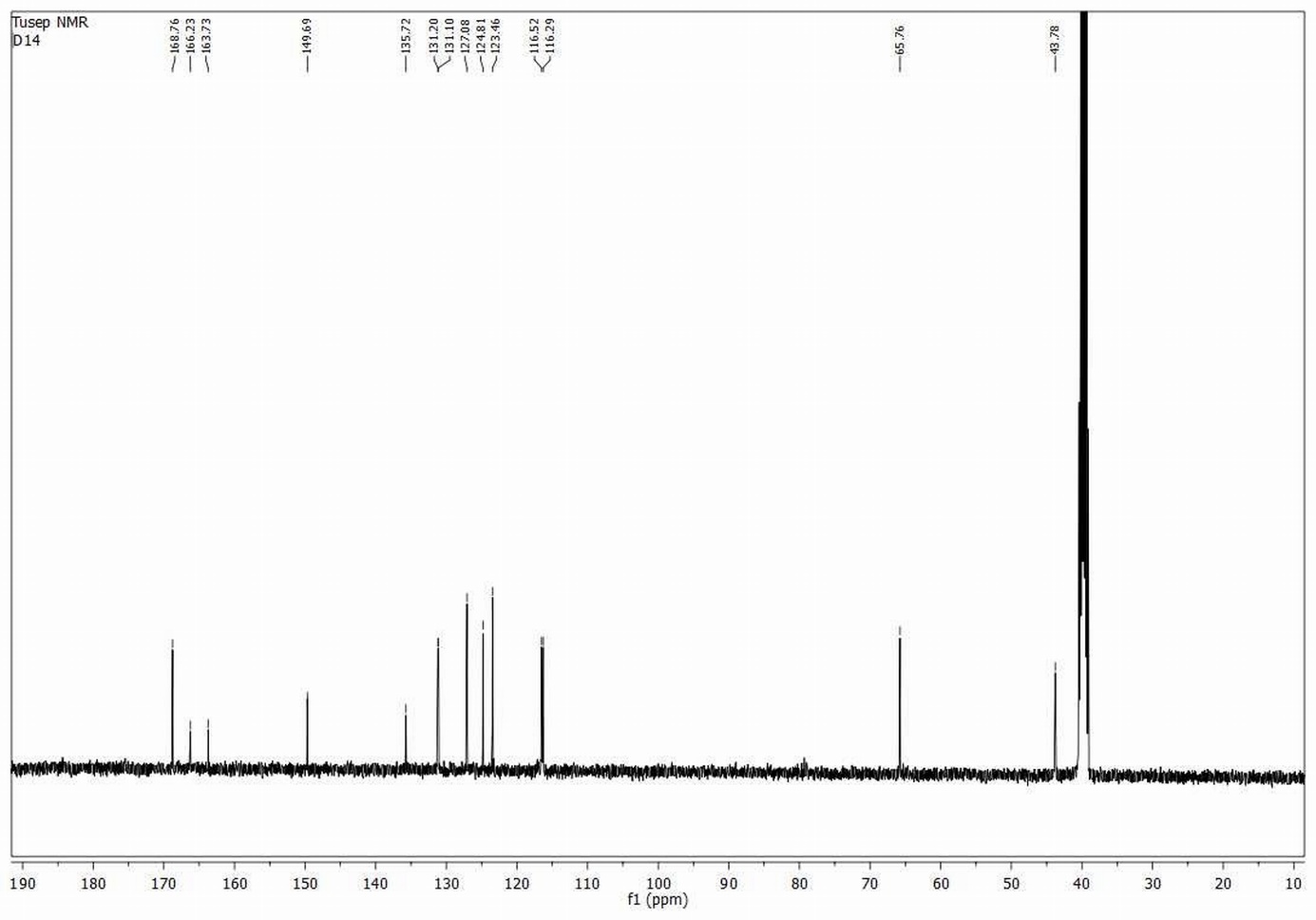


**Figure S52**. ^13^C-NMR spectrum of **6q** (101 MHz, DMSO)


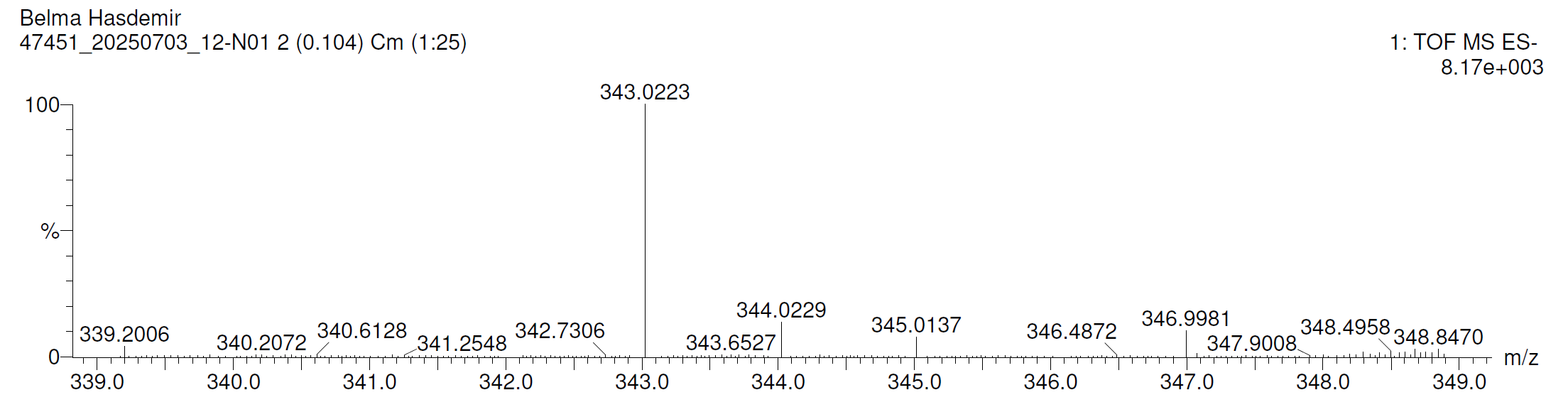


**Figure S53**. HRMS spectrum of **6q**


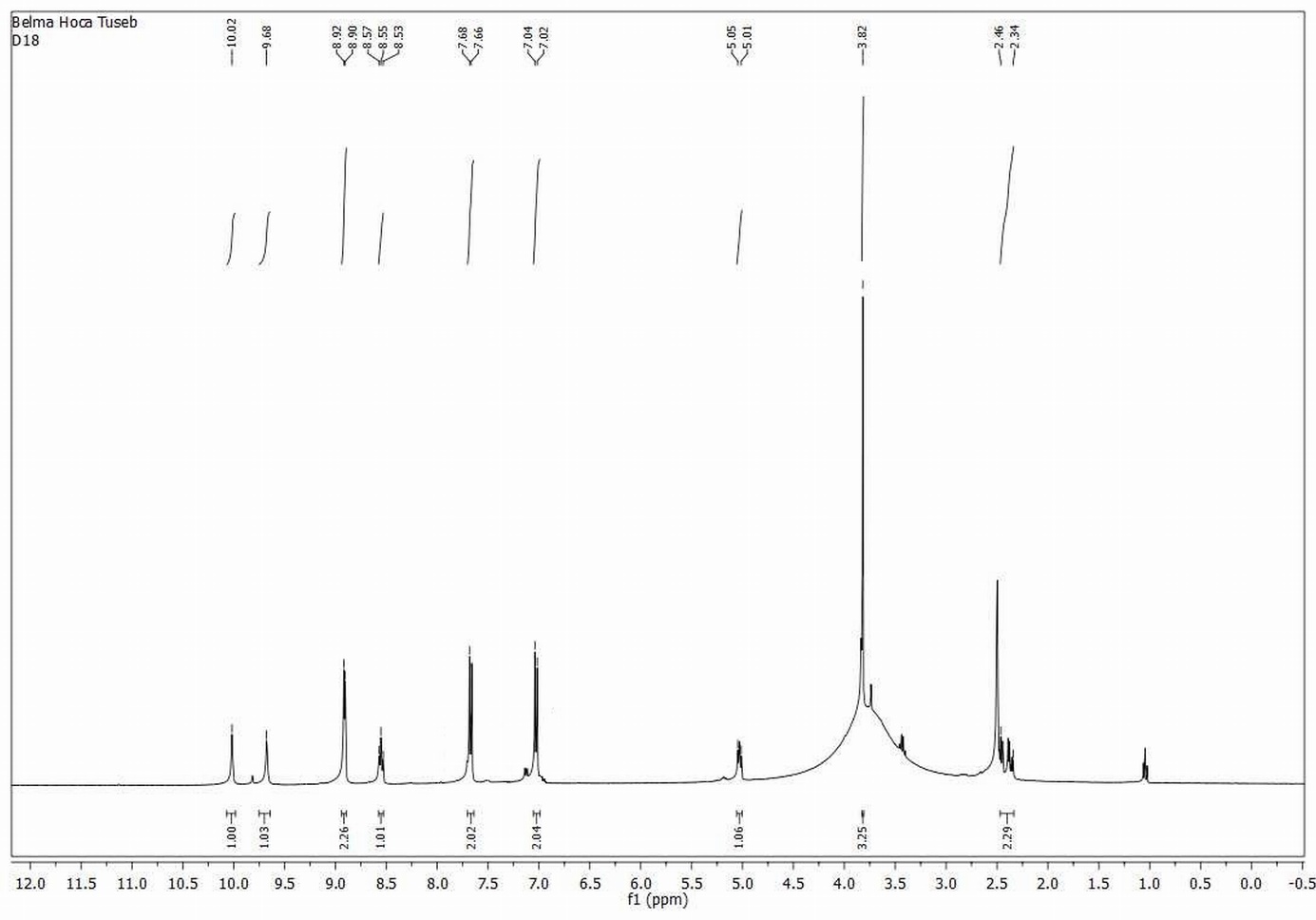


**Figure S54**. ^1^H-NMR spectrum of **6r** (400 MHz, DMSO)


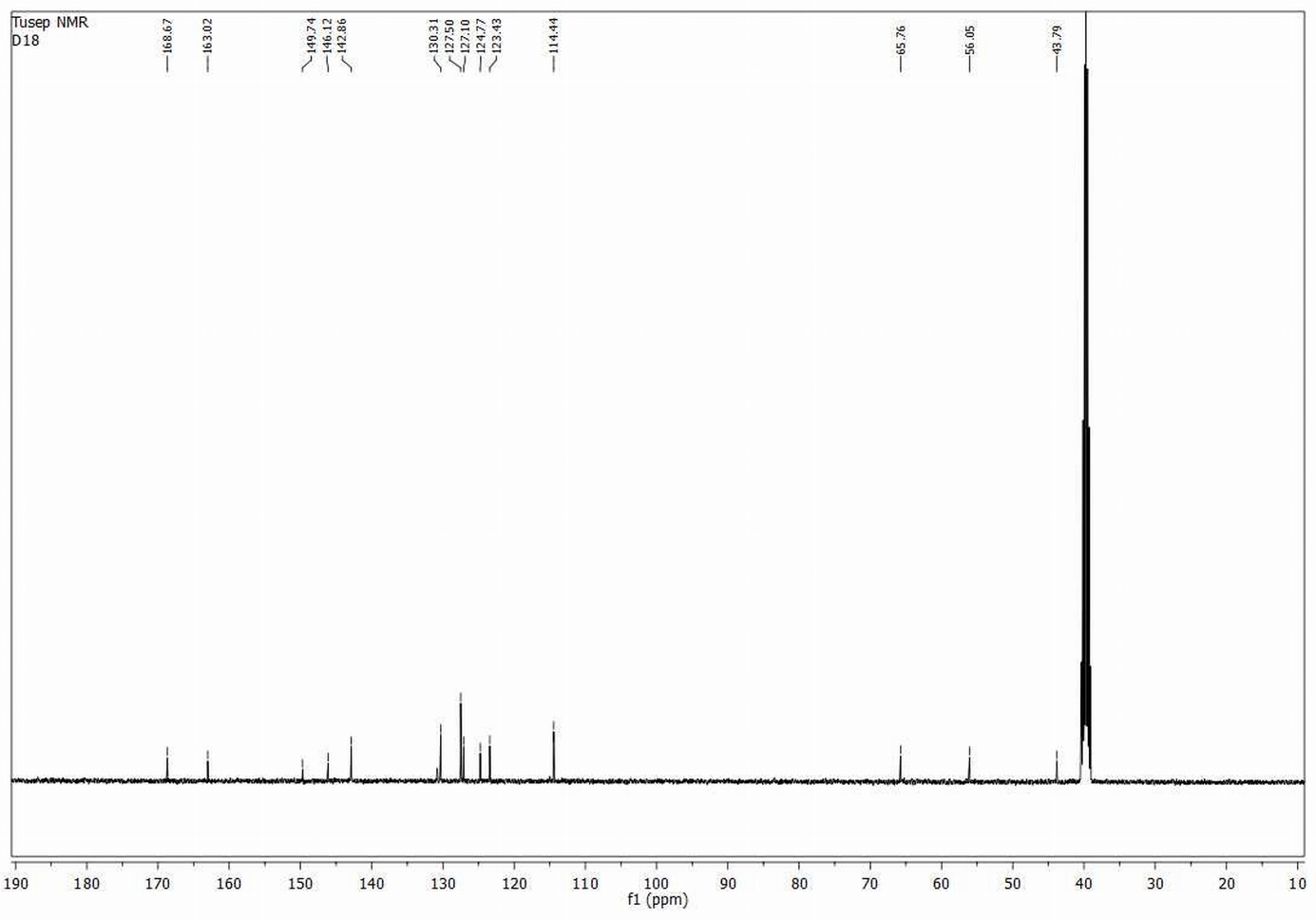


**Figure S55**. ^13^C-NMR spectrum of **6r** (101 MHz, DMSO)


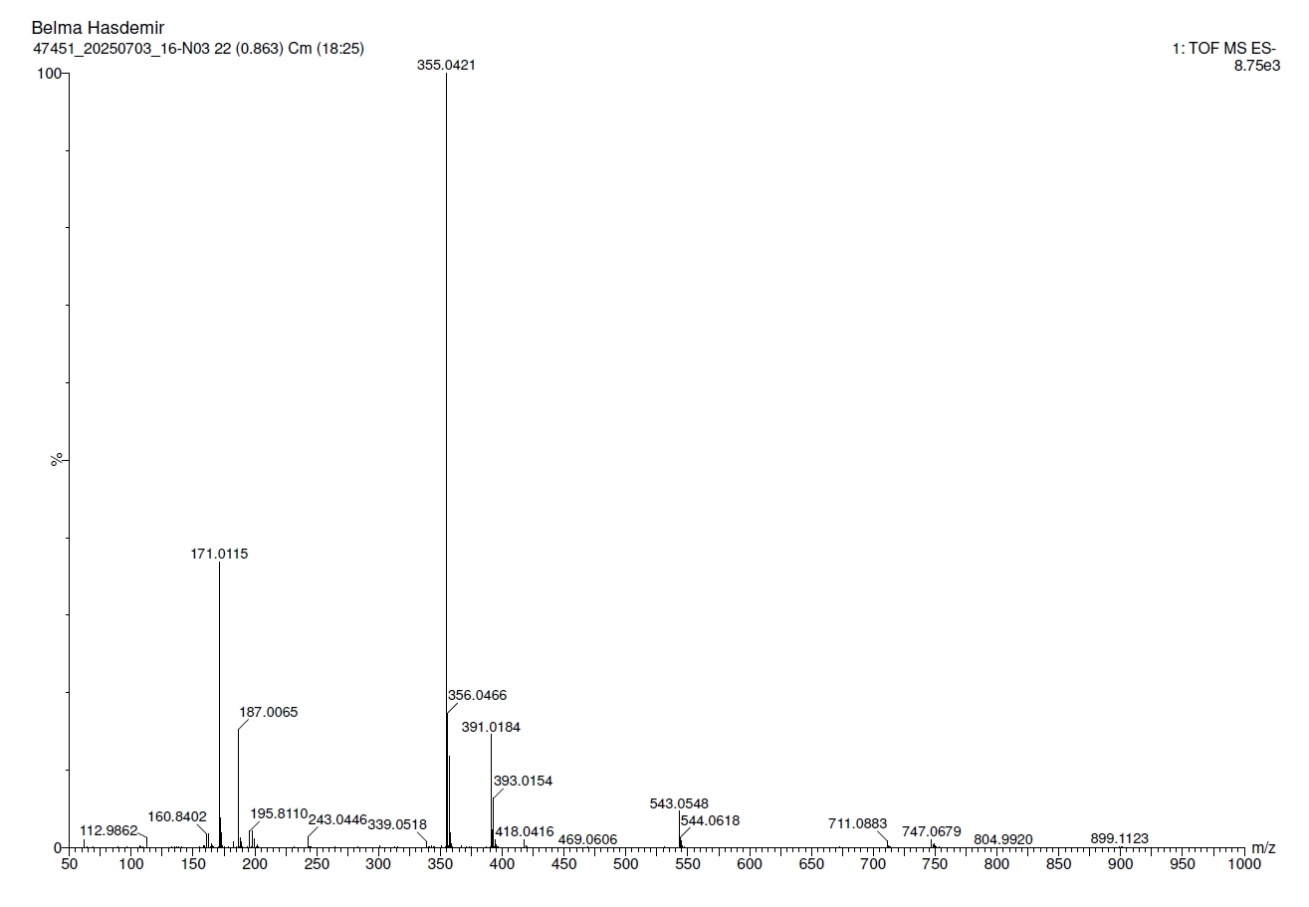


**Figure S56**. HRMS spectrum of **6r**

**3. Biological activity studies**

**3.1. Anticancer activity**

**Table S1** Assessment of Cell Viability After Treatment in Pancreatic Cancer (PANC-1 Cell

Line).

| **Treated Doses (μM) vs % Viability** | **6a** | **6b** | **6c** | **6d** | **6e** | **SKLB**  **1002** | **5-Fluorouracil** |
| --- | --- | --- | --- | --- | --- | --- | --- |
| 0,0 | 100,0 | 100,0 | 100,0 | 100,0 | 100,0 | 100,0 | 100,0 |
| 1,6 | 91,9 | 98,8 | 105,7 | 94,3 | 89,3 | 67,0 | 76,1 |
| 3,1 | 84,3 | 101,7 | 93,8 | 82,5 | 92,6 | 67,8 | 77,8 |
| 6,3 | 69,7 | 98,9 | 92,8 | 81,9 | 89,7 | 53,1 | 78,5 |
| 12,5 | 84,1 | 104,7 | 88,7 | 90,3 | 104,3 | 85,5 | 62,3 |
| 25,0 | 85,9 | 103,4 | 87,0 | 87,9 | 86,8 | 44,7 | 59,7 |
| 50,0 | 86,1 | 126,6 | 89,5 | 93,2 | 93,7 | 33,7 | 46,7 |
| 100,0 | 73,1 | 109,2 | 79,8 | 85,1 | 87,3 | 30,9 | 35,0 |
| IC_50_ (μM) | >100 μM | >100 μM | >100 μM | >100 μM | >100 μM | 10,53 | 42,85 |

**3.2. Antimicrobial activity**

**Table S2.** Antibacterial activity results of *N*-acyl sulfonohydrazides **6a-r** (MIC ​​(µg/mL))

| **Gram-negative bacteria**  (**MIC, 𝜇g/mL)**  ______________________________________________ | | | | **Gram-positive bacteria**  (**MIC, 𝜇g/mL)**  **___________________________________________________** | | | | |
| --- | --- | --- | --- | --- | --- | --- | --- | --- |
| **Sample ID** | ***P. aeruginosa***  **ATCC 27853** | ***E. coli***  **ATCC 25922** | ***K. pneumoniae***  **ATCC 4352** | ***P. mirabilis***  **ATCC 14153** | ***S. aureus***  **ATCC 29213** | ***S. epidermidis***  **ATCC 12228** | ***E. faecalis***  **ATCC 29212** | ***MRSA* ATCC 43300** |
| **6a** | no | no | no | no | 19.53 | 156.2 | no | no |
| **6b** | no | no | no | no | 19.53 | 312.5 | no | no |
| **6c** | no | no | no | no | 78.12 | 156.2 | no | 625 |
| **6d** | no | no | no | no | 19.53 | 156.2 | 625 | no |
| **6e** | no | no | no | no | 78.12 | 156.2 | no | no |
| **6f** | no | no | no | no | 78.12 | 156.2 | no | no |
| **6g** | no | no | no | no | 78.12 | 156.2 | no | no |
| **6h** | 312.5 | no | no | no | 156.2 | no | no | 625 |
| **6i** | no | no | no | no | 625 | 625 | no | no |
| **6j** | no | no | no | no | 156.2 | 156.2 | no | no |
| **6k** | no | no | no | no | 625 | 625 | no | no |
| **6l** | no | no | no | no | 625 | 312.5 | no | 625 |
| **6m** | no | no | no | no | 78.12 | 156.2 | no | no |
| **6n** | 312.5 | no | no | no | 156.2 | 625 | no | 625 |
| **6o** | 312.5 | no | no | no | 625 | 312.5 | no | no |
| **6p** | no | no | no | no | 156.2 | 625 | no | no |
| **6q** | no | no | no | no | 625 | 625 | no | no |
| **6r** | no | no | no | no | 625 | 312.5 | no | 625 |
| Ref.  Antimicrobials | 2.4  Ceftazidi-me | 4.9  Cefuroxime-Na | 4.9  Cefuroxime-Na | 1  Ciprofloxa-cin | 1.2  Cefuroxime-Na | 9.8  Cefuroxime | 128  Amikacin | 1.2  Cefuro-xime-Na |

**Table S3.** Antifungal activity results of hydrazide-sulfonamide compounds **6a-r**

(MIC ​​(𝜇g/mL))

| **Sample ID** | ***C. albicans* ATCC 10231** | ***C. tropicalis* ATCC 750** | ***C. parapsilosis* ATCC 22019** |
| --- | --- | --- | --- |
| **6a** | 625 | 156.2 | 1250 |
| **6b** | 625 | 312.5 | no |
| **6c** | 625 | no | 1250 |
| **6d** | 625 | 156.2 | 1250 |
| **6e** | 625 | 312.5 | 1250 |
| **6f** | 625 | no | 625 |
| **6g** | 625 | no | 625 |
| **6h** | 312.5 | 312.5 | 625 |
| **6i** | no | no | 1250 |
| **6j** | no | 156.2 | 625 |
| **6k** | 625 | no | 1250 |
| **6l** | no | no | 625 |
| **6m** | 625 | 312.5 | 625 |
| **6n** | 625 | no | 1250 |
| **6o** | no | no | 1250 |
| **6p** | no | 156.2 | 625 |
| **6q** | 625 | no | 1250 |
| **6r** | no | no | 625 |
| Ref.  antimicrobials | 4.9  Clotrimazole | 4  Nystatin | 0.5  Fluconazole |

**4. Molecular Docking Studies**

**4.1. Ligand Preparations**

SDF files of ligands were generated using DataWarrior, as we have done previously (Mammadova, Mermer, and Kocabaş 2021). In brief, SMILES codes were converted into 3D structures using a randomized, low-energy conformation approach based on crystallography and torsion. Energy minimization of the conformations was performed using the MMFF94s+ force field. A version 3 SD file containing 3D atomic coordinates was used. Known and effective VEGFR2 inhibitors (IC_50_ values ​​ranging from 12 nM to 40 nM, namely SKLB1002, Doxorubicin, and 5-Fluorouracil) were included as positive controls, representing compounds with established anticancer efficacy in previous studies (Table S5) (Alqahtani et al. 2022; Mettu et al. 2020; Sayed et al. 2021).

**4.2. Docking Parameters and Protein Preparation**

Docking studies were performed using the crystal structure of the human VEGFR2 ligand-binding domain in complex with a benzimidazole-urea inhibitor (PDB ID: 2OH4), and Bcl-2 crystallized with phenyl tetrahydroisoquinoline amide (PDB ID: 2W3L).(Alqahtani et al. 2022; Sayed et al. 2021) In brief, the protein crystal structure was downloaded in PDB format from RCSB PDB.(RSCB PDB Protein data bank n.d.) The receptor structure was checked for loading errors and missing atoms. Molecules, ions, and small ligands were removed to optimize the structure. A grid box of 26 Å × 28 Å × 26 Å for VEGFR2 (2W3L) as well as for VEGFR (PDB ID: 2OH4), Akt2(PDB ID: 3d0e), PDK2(PDB ID: 4mp2), GAA (PDB ID: 5NN5), PBX1(PDB ID: 1DU6), PKNOX1(PDB ID: 1X2N), TGIF1(PDB ID: 2LK2), MYC (PDB ID: 1NKP), and MEIS1(PDB ID: 3K2A) were generated using AutoDockTools 1.5.6. Automatic docking of the ligands, as shown in Table 6, was performed using PaDelADV, following previous protocols (Kocabaş and Ergin 2016; Siyah et al. 2021; Turan et al. 2020).

**4.3. Analysis of Protein-Ligand Interactions**

Protein-ligand interactions were analyzed using the docking poses obtained from AutoDock Vina. The docking output files (PDBQT) were converted to SDF format using Open Babel v3.1.1, with explicit hydrogens added (O’Boyle et al. 2011). The ligand SDF files and corresponding protein PDB structures were uploaded to the Proteins Plus platform, where two-dimensional interaction diagrams were generated using JAMDA, followed by the PoseView and PoseEdit modules (Diedrich et al. 2023; Stierand, Maaß, and Rarey 2006; Stierand and Rarey 2007, 2010). Hydrogen bonds, hydrophobic contacts, and π-π interactions were automatically identified based on geometric criteria defined in PoseEdit. All interaction diagrams were generated using default PoseEdit parameters and exported as scalable vector graphics (SVG) files.

**4.4. Computation Analysis of Drug-Likeness**

The synthesized compounds were characterized to assess their general molecular and drug-likeness properties. Parameters analyzed included molecular weight, calculated partition coefficient (cLogP), calculated solubility (cLogS), polar surface area, and fragment-based drug-likeness scores. In addition, toxicity risk assessments were performed for mutagenicity, tumorigenicity, irritant effects, and reproductive toxicity using DataWarrior software, as we have done previously (Mammadova et al. 2021).

**5. Analysis of compounds for their potent anticancer drug-like properties**

To assess the potential of the tested compounds **6a-r** as multi-target anticancer agents, a molecular docking study was conducted against VEGFR2 (PDB ID: 2OH4) and eight additional cancer-associated targets (Akt2, PDK2, GAA, PBX1, PKNOX1, TGIF1, MYC-MAX, and MEIS1). Well-characterized VEGFR2 inhibitors-SKLB1002, 5-Fluorouracil, and Doxorubicin-were included as reference compounds (Table S4).

The ligands were prepared using DataWarrior, where SMILES codes were converted into low-energy 3D conformers and optimized using the MMFF94s+ force field. Docking was performed with AutoDockTools and PaDelADV following established protocols.

Docking results revealed that many of the **6a-r** compounds exhibited comparable or better binding affinities for VEGFR than those of reference drugs. Among the **6a-r** series, **6i** (-6.3 kcal/mol), **6f** (-6.2 kcal/mol), and **6l** (-6.2 kcal/mol) showed the strongest binding to VEGFR2, exceeding the affinity of SKLB1002 (-6.0 kcal/mol). Notably, **6b**, **6e**, **6h**, **6n**, **6k**, and **6o** also displayed consistent VEGFR2 binding energies around -6.1 kcal/mol (Table 6), suggesting a strong interaction profile across multiple analogs.

In contrast, reference compounds such as 5-Fluorouracil and SKLB1002 demonstrated lower binding affinities across several targets, especially MEIS1 and MYC-MAX (e.g., -3.7 and -4.1 kcal/mol, respectively). Most compounds exhibited relatively moderate binding to these non-VEGFR targets, indicating some selectivity.

A statistical comparison (unpaired t-test) confirmed that **6a-r** compounds had significantly higher binding affinity to VEGFR2 compared to all other targets (p < 0.001 across targets), supporting their potential selectivity for VEGFR2 (Figure S57 and Table S5). Figure S57 is a bar graph summarizing the binding affinities (kcal/mol) of the synthesized compounds **6a-r** across a panel of cancer-associated targets, including VEGFR, Akt2, PDK2, GAA, PBX1, PKNOX1, TGIF1, MYC–MAX, and MEIS1. Blue bars represent the docking scores of the reference compounds SKLB1002 and 5-Fluorouracil, while red bars indicate the mean binding affinity calculated for the **6a-r** series. More negative values correspond to stronger predicted binding. The analysis highlights VEGFR, Akt2, and PDK2 as the most favorable targets for the **6a-r** compounds, with overall comparable or improved affinity profiles relative to the reference ligands across several oncogenic proteins. Figure S58 shows the representative molecular docking poses of the reference inhibitor SKLB1002 and the synthesized compounds **6a-6e** within the VEGFR2 binding pocket. The protein is shown as a ribbon/cartoon representation (red), with residues forming the binding cavity displayed as sticks. Ligands are rendered as stick models within the active site mesh surface. SKLB1002 occupies the hinge region and adjacent hydrophobic subpockets, serving as a reference binding orientation. Compounds **6a-6e** adopt comparable binding modes, positioning their aromatic cores toward the hinge region and extending substituents into the hydrophobic channel, suggesting favorable accommodation within the VEGFR2 active site.

Functional *in vitro* studies, including cell viability and apoptosis assays, demonstrated that compounds **6a-e** exhibit moderate anti-proliferative and pro-apoptotic effects, with **6c** and **6e** showing relatively increased apoptosis in HCT116 cells. While their IC_50_ values were numerically higher than those of reference drugs such as SKLB1002 and 5-fluorouracil, the lack of cytotoxicity toward healthy HDF cells indicates a potentially favorable therapeutic window. Taken together, these results suggest that further chemical modification and optimization of the **6a-r** scaffold could enhance potency while retaining selectivity and safety, supporting their potential as next-generation VEGFR-targeted anticancer therapeutics.

However, the calculated binding energies should be interpreted with caution, as the small numerical differences between compounds are unlikely to be statistically meaningful. Furthermore, no direct correlation was observed between the in silico docking scores and the biological activity measured *in vitro*.

Two-dimensional protein-ligand interaction diagrams generated using the PoseView module of the Proteins Plus platform illustrate the binding interactions of compounds **6a-6e** within the VEGFR2 active site (Figure S59). Hydrogen bonds are shown as blue dashed lines, hydrophobic contacts as green arcs, and π-π stacking interactions as cyan dashed lines.

To better understand the binding modes of the designed molecules, two-dimensional interaction analyses were performed for the best docking poses obtained from AutoDock Vina. The interaction diagrams revealed a conserved binding pattern for most of the tested compounds within the target binding pocket. The reference inhibitor SKLB1002 displayed a characteristic binding configuration stabilized by both polar and hydrophobic interactions (Figure S59). A hydrogen bond was observed between the ligand nitrogen atom and Cys917A, suggesting a stabilizing polar interaction within the pocket. In addition, the aromatic core of SKLB1002 formed a π-π stacking interaction with Phe1045A, which is consistent with the presence of aromatic residues lining the binding site. Several hydrophobic contacts were also detected with surrounding residues, including Phe1045A, Leu1033A, Phe916A, Val914A, Ala864A, and Leu838A, indicating that hydrophobic packing contributes significantly to ligand stabilization.

Compounds **6a** and **6b** showed very similar interaction profiles. Both ligands formed two hydrogen bonds involving the carbonyl oxygen of the ligand with residues Cys917A and Glu915A. In addition, their aromatic ring systems established π-π stacking interactions with Phe1045A, a residue commonly involved in stabilizing aromatic inhibitors in the VEGFR2 pocket. Hydrophobic contacts were also observed with Phe1045A, Leu1033A, and Val897A, indicating favorable packing of the ligand scaffold within the hydrophobic region of the binding site.

Compound **6c** displayed a slightly different interaction pattern. This molecule formed hydrogen bonds with Asp1044A and Cys917A, suggesting an alternative anchoring orientation compared with **6a** and **6b**. Similar to the other compounds, hydrophobic contacts with Phe1045A, Leu1033A, and Val897A were observed, indicating that the aromatic core of the ligand remains positioned within the hydrophobic pocket.

Compounds **6d** and **6e** exhibited the most extensive hydrogen-bonding networks among the tested derivatives. Both molecules formed multiple hydrogen bonds with Arg1049A and Asn921A, while also maintaining an additional interaction with Cys917A. These interactions suggest a deeper engagement with residues located toward the inner region of the binding pocket. Hydrophobic contact with Leu1033A further contributed to the stabilization of these complexes.

Overall, the interaction analysis indicates that residues Cys917A, Glu915A, Asp1044A, Arg1049A, Asn921A, and Phe1045A play important roles in ligand stabilization within the VEGFR2 binding site. The observed combination of hydrogen bonding, π-π stacking, and hydrophobic contacts supports the ability of compounds **6a-6e** to occupy the VEGFR2 active site and form stable protein-ligand complexes.

**5.1. Target compounds demonstrate potential druglike properties**

The physicochemical properties pertinent to drug development for the synthesized compounds **6a-r** were thoroughly assessed (Table S6). This evaluation further corroborates their suitability for progression into preclinical stages. All eighteen compounds satisfied a fundamental criterion by exhibiting molecular weights below 450 Da. Furthermore, favorable lipophilicity was observed across all compounds, with cLogP values consistently below 5. A substantial majority, specifically seventeen out of eighteen compounds, demonstrated acceptable aqueous solubility, indicated by cLogS values exceeding -4. Regarding polarity, none of the compounds presented polar surface area (PSA) values below 60; instead, all registered above 100, reflecting comparatively elevated polarity. Significantly, ten of the eighteen compounds, including **6c**, **6h**, **6i**, **6k**, **6l**, **6n**, **6o**, **6q**, and **6r**, achieved positive drug-likeness scores (>0), thereby underscoring their promise as prospective lead molecules. Importantly, computational toxicological assessments projected minimal risks concerning mutagenicity, tumorigenicity, irritancy, or reproductive effects, thereby bolstering the anticipated safety profile of this series. In summation, these findings illustrate that a substantial proportion of the synthesized molecules adhere to critical drug-likeness parameters and are devoid of major predicted toxicological concerns, thus substantiating their candidacy for subsequent biological investigations.

**Table S4** SMILES codes of molecules used in the docking study and for comparisons.

| **Compounds and references** | **SMILES** |
| --- | --- |
| 6a | O=C(NNS(C1=CC=CC=C1)(=O)=O)C2CCCC2O |
| 6b | O=C(NNS(C1=CC=C(F)C=C1)(=O)=O)C2CCCC2O |
| 6c | O=C(NNS(C1=CC=C(OC)C=C1)(=O)=O)C2CCCC2O |
| 6d | OC1C(C(NNS(C2=CC=CC=C2)(=O)=O)=O)CCCC1 |
| 6e | OC1C(C(NNS(C2=CC=C(F)C=C2)(=O)=O)=O)CCCC1 |
| 6f | OC1C(C(NNS(C2=CC=C(OC)C=C2)(=O)=O)=O)CCCC1 |
| 6g | O=C(NNS(C1=CC=CC=C1)(=O)=O)CC(O)C2=CC=CC=C2 |
| 6h | O=C(NNS(C1=CC=C(F)C=C1)(=O)=O)CC(O)C2=CC=CC=C2 |
| 6i | O=C(NNS(C1=CC=C(OC)C=C1)(=O)=O)CC(O)C2=CC=CC=C2 |
| 6j | O=C(NNS(C1=CC=CC=C1)(=O)=O)CC(O)C2=CC=C(OC)C=C2 |
| 6k | O=C(NNS(C1=CC=C(F)C=C1)(=O)=O)CC(O)C2=CC=C(OC)C=C2 |
| 6l | O=C(NNS(C1=CC=C(OC)C=C1)(=O)=O)CC(O)C2=CC=C(OC)C=C2 |
| 6m | O=C(NNS(C1=CC=CC=C1)(=O)=O)CC(O)C2=CC=C(F)C=C2 |
| 6n | O=C(NNS(C1=CC=C(F)C=C1)(=O)=O)CC(O)C2=CC=C(F)C=C2 |
| 6o | O=C(NNS(C1=CC=C(OC)C=C1)(=O)=O)CC(O)C2=CC=C(F)C=C2 |
| 6p | O=C(NNS(C1=CC=CC=C1)(=O)=O)CC(O)C2=CC=CS2 |
| 6r | O=C(NNS(C1=CC=C(F)C=C1)(=O)=O)CC(O)C2=CC=CS2 |
| 6s | O=C(NNS(C1=CC=C(OC)C=C1)(=O)=O)CC(O)C2=CC=CS2 |
| SKLB1002 | CC1=NN= C( S1)SC2=NC=NC3=CC(=C(C=C32)OC)OC |
| 5-Fluorouracil | C1=C( C( =O)NC(=O)N1)F |

**Table S5** Molecular docking of compounds to VEGFR and other cancer drug targets

| Ligand /  Affinity (kcal/mol ) | VEGFR  (2OH4) | Akt2  (3d0e) | PDK2  (4mp2) | GAA  (5NN5) | PBX1  (1DU6) | PKNOX1  (1X2N) | TGIF1  (2LK2) | MYC-MAX  (1NKP) | MEIS1  (3K2A) |
| --- | --- | --- | --- | --- | --- | --- | --- | --- | --- |
| SKLB1002 | -6.0 | -5.8 | -5.9 | -5.4 | -5.4 | -5.7 | -5 | -5 | -4.1 |
| 5-Fluorouracil | -5.0 | -5.1 | -4.9 | -5.3 | -4.6 | -3.5 | -4.1 | -3.9 | -3.7 |
| **6a** | -5.8 | -5.4 | -5.4 | -4.9 | -4.7 | -3.8 | -4.5 | -4.1 | -3.7 |
| **6b** | -6.1 | -5.6 | -5.7 | -5 | -4.8 | -4.1 | -4.7 | -4.3 | -3.9 |
| **6c** | -5.4 | -4.8 | -5.5 | -5.4 | -4.5 | -3.9 | -4.4 | -4 | -3.7 |
| **6d** | -5.8 | -5.4 | -5.4 | -4.8 | -4.7 | -3.9 | -4.5 | -4 | -3.7 |
| **6e** | -6.1 | -5.6 | -5.7 | -5.1 | -4.8 | -4.1 | -4.7 | -4.2 | -3.9 |
| **6f** | -6.2 | -5.6 | -5.3 | -4.9 | -4.7 | -4.1 | -4.6 | -4 | -3.8 |
| **6g** | -5.8 | -5.4 | -5.4 | -4.8 | -4.7 | -3.8 | -4.5 | -4.7 | -3.7 |
| **6h** | -6.1 | -5.6 | -5.8 | -5.1 | -4.8 | -4.1 | -4.7 | -4.2 | -3.9 |
| **6i** | -6.3 | -6 | -6.1 | -5.4 | -4.6 | -4.1 | -4.5 | -4.5 | -4.2 |
| **6j** | -5.8 | -5.3 | -5.4 | -4.8 | -4.5 | -3.8 | -4.5 | -4.7 | -3.7 |
| **6k** | -6.1 | -5.6 | -5.7 | -5.1 | -4.8 | -4 | -4.7 | -4.2 | -3.9 |
| **6l** | -6.2 | -5.5 | -5.3 | -4.9 | -4.7 | -4.1 | -4.6 | -4.2 | -3.7 |
| **6m** | -5.8 | -5.4 | -5.4 | -4.9 | -4.7 | -3.8 | -4.5 | -4.7 | -3.7 |
| **6n** | -6.1 | -5.6 | -5.7 | -5 | -4.8 | -4.1 | -4.7 | -4.2 | -3.8 |
| **6o** | -6.1 | -5.6 | -5.3 | -4.8 | -4.7 | -4.1 | -4.6 | -4.2 | -3.8 |
| **6p** | -5.8 | -5.4 | -5.4 | -4.9 | -4.7 | -3.8 | -4.5 | -4.1 | -3.7 |
| **6q** | -5.0 | -5 | -5.5 | -4.9 | -4.3 | -3.8 | -4 | -4.5 | -3.7 |
| **6r** | -5.3 | -5.1 | -5.4 | -4.9 | -4 | -3.8 | -4 | -3.8 | -3.6 |
| t-test  (VEGF2 vs Others 6a-s) | - | 1.7E-04 | 7.8E-04 | 2.4E-11 | 9.3E-15 | 1.6E-21 | 5.9E-16 | 3.3E-17 | 9.2E-23 |


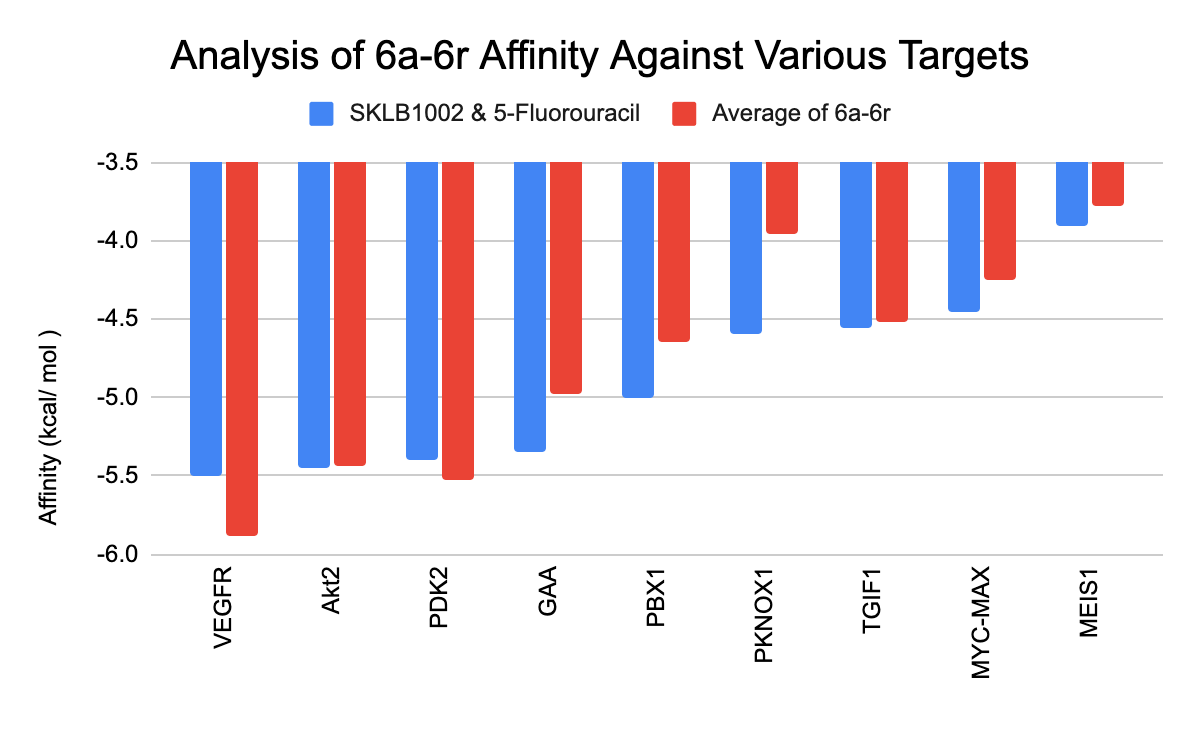


**Figure S57** Comparative binding affinity analysis of compounds **6a-r** against multiple cancer-related protein targets.


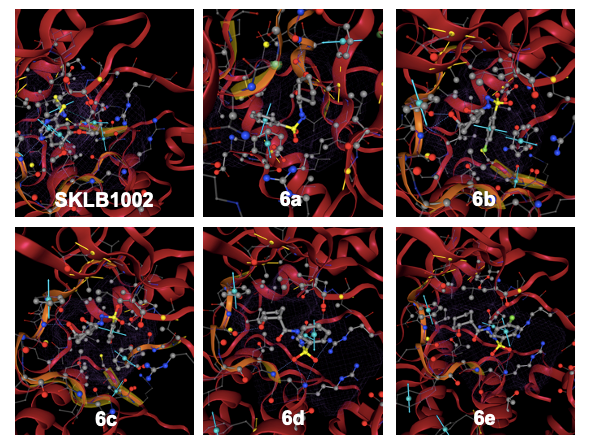


**Figure S58** Docking poses of reference ligands and compounds **6a-e** in the VEGFR2 active

site

**
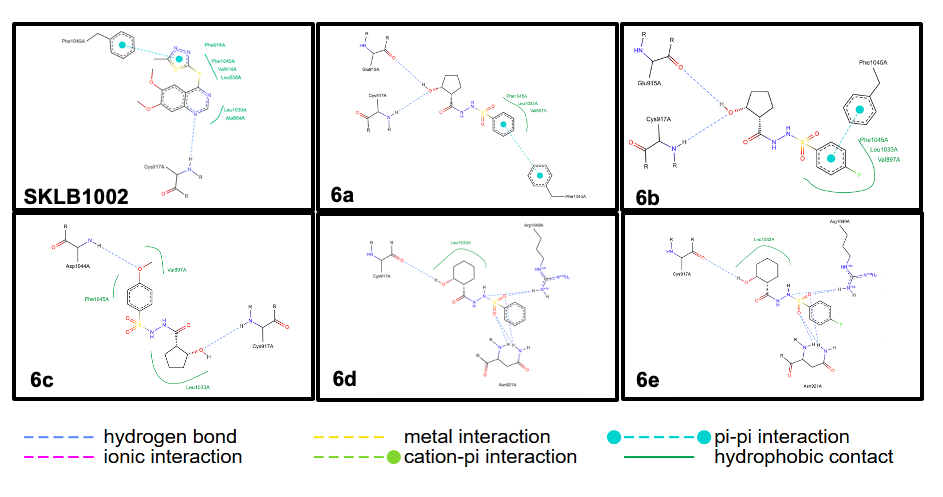
**

**Figure S59** Two-dimensional protein-ligand interaction diagrams of docked compounds

**Table S6** Drug-likeness analysis of studied compounds **6a-r**

| Comp. | Total MW* | cLogP  ** | cLogS  *** | Polar Surface Area**** | Druglikeness  ***** | Muta-  genic | Tumori-genic | Reproduct.ve Effective | Irritant | |
| --- | --- | --- | --- | --- | --- | --- | --- | --- | --- | --- |
| **6a** | 284.3 | -0.6 | -1.0 | 103.9 | -5.9 | none | none | none | none |  |
| **6b** | 302.3 | -0.5 | -1.3 | 103.9 | -0.9 | none | none | none | none |  |
| **6c** | 314.4 | -0.7 | -1.0 | 113.1 | 1.8 | none | none | none | none |  |
| **6d** | 298.4 | -0.3 | -1.3 | 103.9 | -8.3 | none | none | none | none |  |
| **6e** | 316.4 | -0.2 | -1.6 | 103.9 | -3.2 | none | none | none | none |  |
| **6f** | 328.4 | -0.3 | -1.3 | 113.1 | -0.6 | none | none | none | none |  |
| **6g** | 320.4 | 0.1 | -1.2 | 103.9 | -2.5 | none | none | none | none |  |
| **6h** | 338.4 | 0.2 | -1.5 | 103.9 | 2.5 | none | none | none | none |  |
| **6i** | 350.4 | 0.0 | -1.2 | 113.1 | 5.2 | none | none | none | none |  |
| **6j** | 350.4 | 0.0 | -1.2 | 113.1 | -2.0 | none | none | none | none |  |
| **6k** | 368.4 | 0.1 | -1.5 | 113.1 | 3.0 | none | none | none | none |  |
| **6l** | 380.4 | -0.1 | -1.2 | 122.3 | 5.5 | none | none | none | none |  |
| **6m** | 338.4 | 0.2 | -1.5 | 103.9 | -2.1 | none | none | none | none |  |
| **6n** | 356.3 | 0.3 | -1.8 | 103.9 | 4.0 | none | none | none | none |  |
| **6o** | 368.4 | 0.1 | -1.5 | 113.1 | 5.4 | none | none | none | none |  |
| **6p** | 326.4 | -0.1 | -1.2 | 132.1 | -1.7 | none | none | none | none |  |
| **6q** | 344.4 | 0.0 | -1.5 | 132.1 | 3.3 | none | none | none | none |  |
| **6r** | 356.4 | -0.1 | -1.2 | 141.4 | 5.9 | none | none | none | none |  |

*Good < 450, **Good < 5, ***Good > –4, ****Good < 60, *****Good > 0).

**Reference**

Alqahtani, Ali S., Mostafa M. Ghorab, Fahd A. Nasr, Mohammad Z. Ahmed, Abdullah A. Al-Mishari, and Sabry M. Attia. 2022. “Novel Sulphonamide-Bearing Methoxyquinazolinone Derivatives as Anticancer and Apoptosis Inducers: Synthesis, Biological Evaluation and in Silico Studies.” *Journal of Enzyme Inhibition and Medicinal Chemistry* 37(1):86–99. doi:10.1080/14756366.2021.1983807.

Diedrich, Konrad, Bennet Krause, Ole Berg, and Matthias Rarey. 2023. “PoseEdit: Enhanced Ligand Binding Mode Communication by Interactive 2D Diagrams.” *Journal of Computer-Aided Molecular Design 2023 37:10* 37(10):491–503. doi:10.1007/s10822-023-00522-4.

Fraga, Carlos A. M., Lis Helena P. Teixeira, Carla Maria de S. Menezes, Carlos Mauricio R. Sant’Anna, Maria da Conceição K. V. Ramos, Francisco R. de Aquino Neto, and Eliezer J. Barreiro. 2004. “Studies on Diastereoselective Reduction of Cyclic β-Ketoesters with Boron Hydrides. Part 4: The Reductive Profile of Functionalized Cyclohexanone Derivatives.” *Tetrahedron* 60(12):2745–55. doi:10.1016/j.tet.2004.01.079.

Hasdemir, Belma. 2015. “Asymmetric Synthesis of Some Chiral Aryl and Hetero Aryl-Substituted β -, γ -, δ -Hydroxy Esters.” *Synthetic Communications* 45(9):1082–88. doi:10.1080/00397911.2014.1003353.

Hasdemir, Belma, Hülya Çelik Onar, and Ayşe Yusufoğlu. 2012. “Asymmetric Synthesis of Long Chain β-Hydroxy Fatty Acid Methyl Esters as New Elastase Inhibitors.” *Tetrahedron: Asymmetry* 23(14):1100–1105. doi:10.1016/j.tetasy.2012.07.004.

Hasdemir, Belma, and Ayşe Yusufoğlu. 2004. “Asymmetric Synthesis of Monohydroxy Tetradecanoic Acids and Their Methyl Esters.” *Tetrahedron: Asymmetry* 15(1):65–68. doi:10.1016/j.tetasy.2003.10.020.

Kocabaş, Fatih, and Enes Kemal Ergin. 2016. “Identification of Small Molecule Binding Pocket for Inhibition of Crimean–Congo Hemorrhagic Fever Virus OTU Protease.” *Turkish Journal of Biology* 40:239–49. doi:10.3906/biy-1501-56.

Mammadova, Aynura, Arif Mermer, and Fatih Kocabaş. 2021. “Screening of the Small Molecule Library of Meinox Enables the Identification of Anticancer Compounds in Pathologically Distinct Cancers.” *Turkish Journal of Biology* 45:633–43. doi:10.3906/biy-2104-14.

Mettu, Akhila, Venu Talla, Soujanya Thumma, and Subhashini Naikal James Prameela. 2020. “Mechanistic Investigations on Substituted Benzene Sulphonamides as Apoptosis Inducing Anticancer Agents.” *Bioorganic Chemistry* 95:103539. doi:10.1016/j.bioorg.2019.103539.

O’Boyle, Noel M., Michael Banck, Craig A. James, Chris Morley, Tim Vandermeersch, and Geoffrey R. Hutchison. 2011. “Open Babel: An Open Chemical Toolbox.” *Journal of Cheminformatics 2011 3:1* 3(1):33-. doi:10.1186/1758-2946-3-33.

RSCB PDB Protein data bank. n.d. Retrieved September 24, 2025. https://www.rcsb.org.

Sayed, Asmaa M., Fatma A. Taher, Mohammad R. K. Abdel-Samad, Mohamed S. A. El-Gaby, Khaled El‐Adl, and Nashwa M. Saleh. 2021. “Design, Synthesis, Molecular Docking, in Silico ADMET Profile and Anticancer Evaluations of Sulfonamide Endowed with Hydrazone-Coupled Derivatives as VEGFR-2 Inhibitors.” *Bioorganic Chemistry* 108:104669. doi:10.1016/j.bioorg.2021.104669.

Siyah, Pinar, Sezer Akgol, Serdar Durdagi, and Fatih Kocabas. 2021. “Identification of First-in-Class Plasmodium OTU Inhibitors with Potent Anti-Malarial Activity.” *Biochemical Journal* 478(18):3445–66. doi:10.1042/BCJ20210481.

Stierand, Katrin, Patrick C. Maaß, and Matthias Rarey. 2006. “Molecular Complexes at a Glance: Automated Generation of Two-Dimensional Complex Diagrams.” *Bioinformatics* 22(14):1710–16. doi:10.1093/bioinformatics/btl150.

Stierand, Katrin, and Matthias Rarey. 2007. “From Modeling to Medicinal Chemistry: Automatic Generation Oft Wo-Dimensional Complex Diagrams.” *ChemMedChem* 2(6):853–60. doi:10.1002/cmdc.200700010.

Stierand, Katrin, and Matthias Rarey. 2010. “Drawing the PDB: Protein−Ligand Complexes in Two Dimensions.” *ACS Medicinal Chemistry Letters* 1(9):540–45. doi:10.1021/ml100164p.

Turan, Raife Dilek, Esra Albayrak, Merve Uslu, Pinar Siyah, Lamia Yazgi Alyazici, Batuhan Mert Kalkan, Galip Servet Aslan, Dogacan Yucel, Merve Aksoz, Emre Can Tuysuz, Neslihan Meric, Serdar Durdagi, Zafer Gulbas, and Fatih Kocabas. 2020. “Development of Small Molecule MEIS Inhibitors That Modulate HSC Activity.” *Scientific Reports* 10(1):7994. doi:10.1038/s41598-020-64888-3.
